# Discovery of LLC0424 as a Potent and Selective *in Vivo* NSD2 PROTAC Degrader

**DOI:** 10.1021/acs.jmedchem.3c01765

**Published:** 2024-04-30

**Authors:** Lianchao Liu, Abhijit Parolia, Yihan Liu, Caiyun Hou, Tongchen He, Yuanyuan Qiao, Sanjana Eyunni, Jie Luo, Chungen Li, Yongxing Wang, Fengtao Zhou, Weixue Huang, Xiaomei Ren, Zhen Wang, Arul M. Chinnaiyan, Ke Ding

**Affiliations:** † State Key Laboratory of Chemical Biology, Shanghai Institute of Organic Chemistry, Chinese Academy of Sciences, no. 345 Lingling Road., Shanghai 200032, People’s Republic of China; ‡ Michigan Center for Translational Pathology, University of Michigan, Ann Arbor, Michigan 48109, United States; § Department of Pathology, University of Michigan, Ann Arbor, Michigan 48109, United States; ∥ Rogel Cancer Center, University of Michigan, Ann Arbor, Michigan 48109, United States; ⊥ Department of Urology, University of Michigan, Ann Arbor, Michigan 48109, United States; # Cancer Biology Program, University of Michigan, Ann Arbor, Michigan 48109, United States; ∇ International Cooperative Laboratory of Traditional Chinese Medicine Modernization and Innovative Drug Discovery of Chinese Ministry of Education (MOE), Guangzhou City Key Laboratory of Precision Chemical Drug Development, College of Pharmacy, Jinan University, 855 Xingye Avenue East, Guangzhou 511400, People’s Republic of China; ○ Molecular and Cellular Pathology Program, University of Michigan, Ann Arbor, Michigan 48109, United States; ◆ Livzon Research Institute, Livzon Pharmaceutical Group Inc., no. 38 Chuangye North Road, Jinwan District, Zhuhai 519000, China; ¶ Howard Hughes Medical Institute, University of Michigan, Ann Arbor, Michigan 48109, United States; □ Hangzhou Institute of Medicine (HlM), Chinese Academy of Sciences, Hangzhou, Zhejiang 310022, China

## Abstract

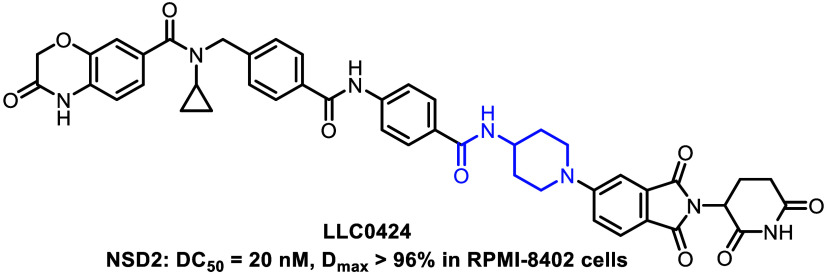

Nuclear receptor-binding SET domain-containing 2 (NSD2), a methyltransferase that primarily installs the dimethyl mark on lysine 36 of histone 3 (H3K36me2), has been recognized as a promising therapeutic target against cancer. However, existing NSD2 inhibitors suffer from low activity or inferior selectivity, and none of them can simultaneously remove the methyltransferase activity and chromatin binding function of NSD2. Herein we report the discovery of a novel NSD2 degrader **LLC0424** by leveraging the proteolysis-targeting chimera technology. **LLC0424** potently degraded NSD2 protein with a DC_50_ value of 20 nM and a *D*_max_ value of 96% in acute lymphoblastic leukemia (ALL) RPMI-8402 cells. Mechanistic studies revealed **LLC0424** to selectively induce NSD2 degradation in a cereblon- and proteasome-dependent fashion. **LLC0424** also caused continuous downregulation of H3K36me2 and growth inhibition of ALL cell lines with NSD2 mutation. Importantly, intravenous or intraperitoneal injection of **LLC0424** showed potent NSD2 degradation *in vivo*.

## Introduction

The nuclear receptor-binding SET [Su(var)3–9, Enhancerofzeste, and Trithorax] domain-containing 2 (NSD2) is a histone methyltransferase of the NSD family that also consists of NSD1 and NSD3.^1^ NSD2, also known as multiple myeloma SET domain (MMSET) or Wolf–Hirschhorn syndrome candidate 1 (WHSC1), contains the SET domain that catalyzes the dimethylation of histone 3 lysine36 (H3K36me2) and two proline–tryptophan–tryptophan–proline (PWWP) domains and five plant homeodomain (PHD) domains that interact with chromatin.^2^ The aberrant expression, somatic mutation, or translocation of NSD2 could increase H3K36me2 level, which reprograms the epigenome to facilitate tumor progression in various types of human cancer.^3^ For instance, the t(4:14) NSD2 translocation that results in NSD2 overexpression has been identified in 11–15% of patients with newly diagnosed multiple myeloma.^4^ In addition, wild-type NSD2 has been reported as a potent oncogene in prostate cancer cells.^5^ NSD2 promotes prostate cancer metastasis and is significantly up-regulated in cancer specimens with highest expression in the metastatic castration-resistant tumors.^5,6^ Furthermore, a point mutation at position 1099 in the catalytic SET domain of NSD2 (E1099K) that leads to NSD2 gain-of-function has been reported in individuals with acute lymphoblastic leukemia (ALL) and lung adenocarcinoma.^7−9^ Genetic knockdown of NSD2 was found to reduce H3K36me2 level and suppress tumor growth *in vivo*.^10^ Therefore, NSD2 emerges as a potential therapeutic target for human cancer.

**Figure 1:**
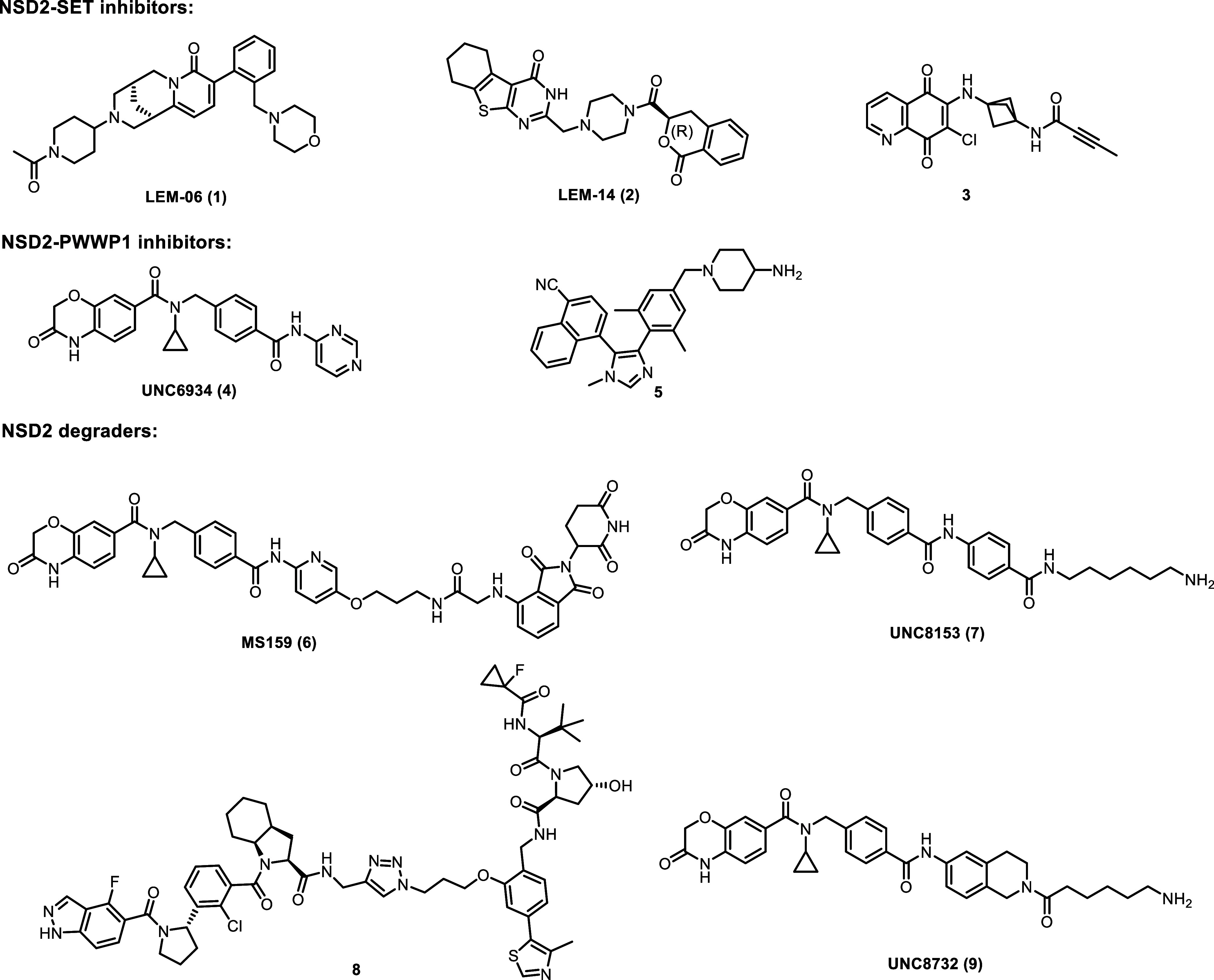
Chemical structures of representative NSD2 inhibitors and degraders.

Several small-molecule NSD2 inhibitors have been discovered to target either the SET domain (**1**, **2**, **3**) or PWWP1 domain (**4**, **5**) of NSD2 (Figure 1).^11−15^ However, these inhibitors suffer from low activity or inferior selectivity.^16−18^ While compound KTX-1001, a novel NSD2-SET inhibitor developed by K36 Therapeutics, Inc., was advanced into phase I clinical trials to treat patients with relapsed and refractory multiple myeloma in 2022 (ClinicalTrials.gov identifier: NCT05651932), its structure and preclinical data have not been disclosed. Moreover, targeting either of these domains cannot simultaneously remove the methyltransferase activity and chromatin binding function of NSD2.

Recently, NSD2 degraders, a new type of NSD2 modulator that could abolish the catalytic and noncatalytic functions of NSD2, have been reported (Figure 1). In 2022, Jin and co-workers reported a first-in-class NSD2 degrader MS159 (**6**) by employing proteolysis targeting chimera (PROTAC) approach.^19^ While compound **6** dose-dependently degraded NSD2 and was more effective in suppressing the growth of cancer cells than the parent NSD2 binder, its degradative activity on NSD2 was relatively low (DC_50_ = 5.2 μM in 293FT cells after 48 h treatment). Moreover, the global proteomics selectivity and the *in vivo* NSD2 degradative effects of compound **6** have not been evaluated. In 2023, Lindsey and co-workers discovered a new NSD2 degrader UNC8153 (**7**) that depleted NSD2 through a novel mechanism.^20^ Compound **7** degraded NSD2 with a DC_50_ value of 0.35 μM and a *D*_max_ value of 79% in U2OS cells after 24 h treatment and exhibited high selectivity in global proteomics experiments. However, the *in vivo* NSD2 degradative effects of compound **7** have not been disclosed. Very recently, the authors conducted further optimization on compound **7** that yielded a more potent analog UNC8732 (**9**), while the *in vivo* NSD2 degradative activity of this compound remains unknown.^21^ In addition, Cloos and co-workers reported the discovery of a NSD2 degrader from a novel selective DNA-encoded library (DEL) hit.^22^ Here, we report the discovery of compound **10l** (**LLC0424**) as a novel, potent, and selective *in vivo* NSD2 degrader by leveraging PROTAC technology. **LLC0424** effectively degraded NSD2 with a DC_50_ value of 20 nM and a *D*_max_ value of 96% in ALL RPMI-8402 cells, and demonstrated high selectivity in global proteomics experiments. Moreover, **LLC0424** downregulated H3K36me2 level globally and led to growth inhibition of ALL cell lines with NSD2 mutation. Importantly, **LLC0424** exhibited promising NSD2 degradative effects *in vivo*.

## Results and Discussion

### Design of NSD2 PROTAC Degraders

**Figure 2:**
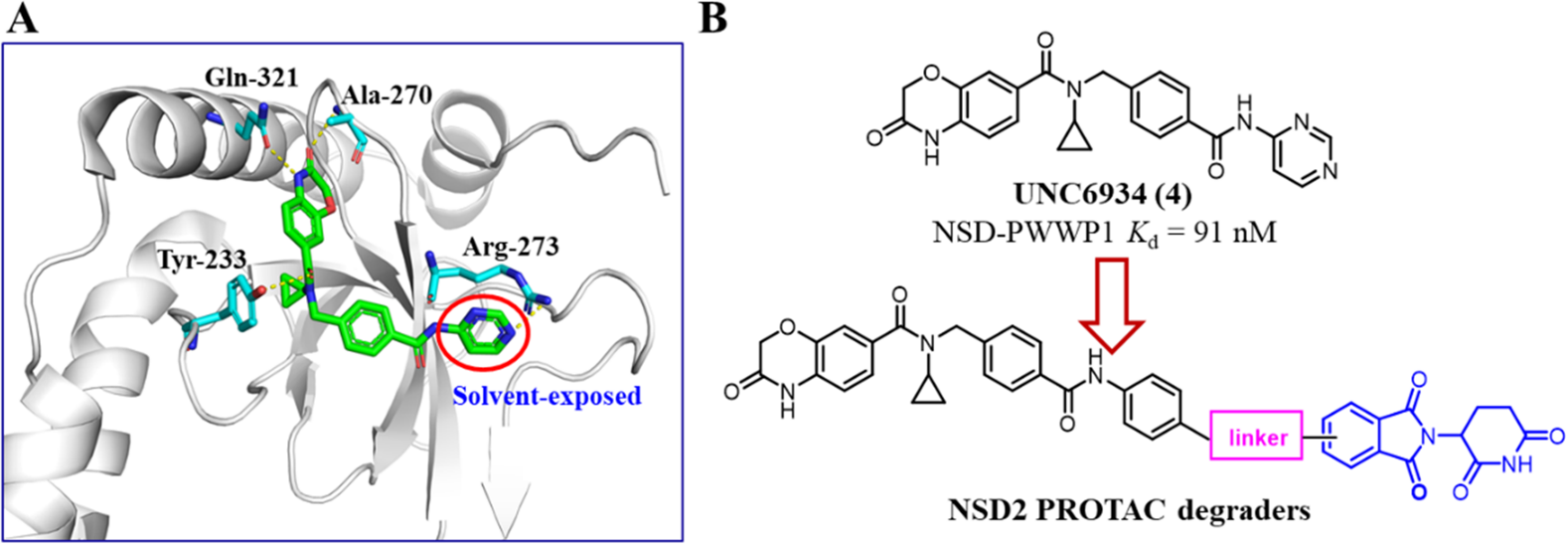
Design of NSD2 PROTACs. (A) Crystal structure of the NSD2-PWWP1 domain in complex with UNC6934 (**4**) (PDB 6XCG). (B) Chemical structures of compound **4** and the designed NSD2 PROTACs.

According to the PROTAC composition, a NSD2 PROTAC degrader can be constructed by connecting a NSD2 binder with an E3 ligase ligand through a linker.^23^ Compound **4** was reported to selectively bind to NSD2-PWWP1 domain with a *K*_d_ value of 91 nM and was thus selected as the NSD2 binder.^14^ It is worth noting that compound **4** has been successfully utilized in the design of NSD2 degraders **6** and **7**.^19−21^ The cocrystal structure of the NSD2-PWWP1 domain with compound **4** revealed the pyrimidine ring to be solvent-exposed, suggesting that this site can be employed for the E3 ligase ligand tethering (Figure 2A). Based on these observations, a series of NSD2 degraders was designed by tethering compound **4** to a cereblon (CRBN) ligand thalidomide via a diverse set of linkers (Figure 2B).

### Chemical Synthesis

**Scheme 1:**
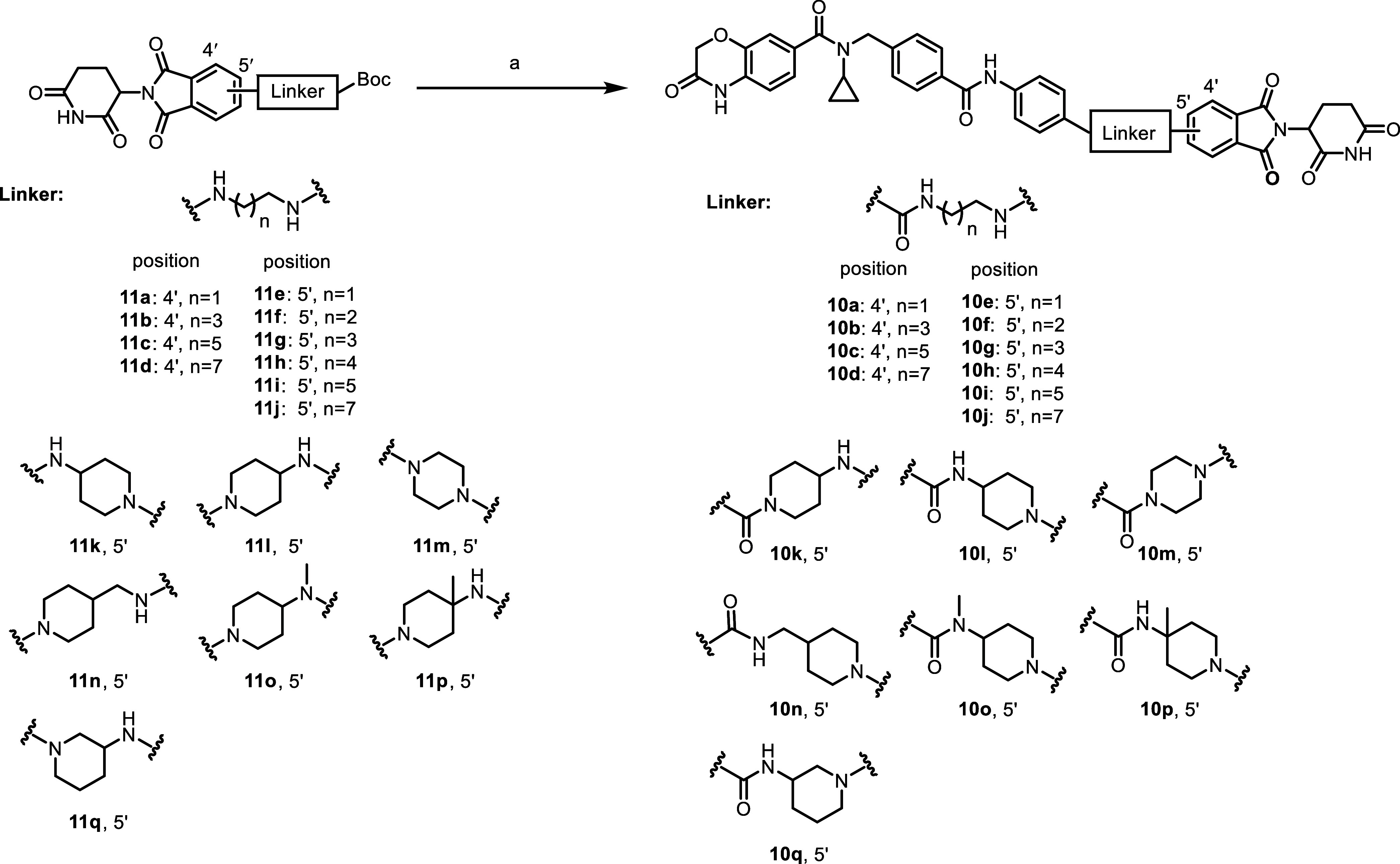
Synthesis of Compounds **10a**–**q** Reagents and conditions: (a) (1) trifluoroacetic acid (TFA), dichloromethane (CH_2_Cl_2_), room temperature (rt), 2 h; (2) 4-(4-((*N*-cyclopropyl-3-oxo-3,4-dihydro-2*H*-benzo[*b*][1,4]oxazine-7-carboxamido)methyl)benzamido)benzoic acid, *N*-[(dimethylamino)-3-oxo-1*H*-1,2,3-triazolo[4,5-*b*]pyridin-1-yl-methylene]-*N*-methylmethanaminium hexafluorophosphate (HATU), triethylamine (Et_3_N), *N*,*N*-dimethylformamide (DMF), rt, 3 h, 67–88% (two steps).

The synthesis of compounds **10a**–**q** was depicted in Scheme 1. Various in-house Boc-protected thalidomide derivatives went through Boc-deprotection reaction and then amide coupling with 4-(4-((*N*-cyclopropyl-3-oxo-3,4-dihydro-2*H*-benzo[*b*][1,4]oxazine-7-carboxamido)methyl)benzamido)benzoic acid,^14^ yielding final compounds **10a**–**q.**

### Structure–Degradation Relationship Study of NSD2 Degraders

We first designed and synthesized a series of NSD2 degraders by connecting compound **4** to 4′-position of thalidomide via linear linkers of different lengths. As NSD2 has been reported as an oncogene that leads to prostate cancer progression,^6^ we used VCaP prostate cancer cell line to assess the efficiency of our compounds. The NSD2 degradation efficiency was determined by immunoblotting after treatment with the compounds at 2 μM in VCaP cells for 24 h, and the results were summarized in Table 1. It was shown that this series of compounds (**10a**–**10d**) exhibited weak degradative effects on NSD2, among which the most potent compound **10b** and **10c** only degraded NSD2 by 25% at 2 μM. We further prepared compounds **10e**–**10j** by switching the attachment point of compound **4** from 4′-position to 5′- position of thalidomide. These compounds turned out to be much more effective than compound **10b** for NSD2 degradation, and the most potent compound **10f** depleted NSD2 by 94% (Table 1).

**Table 1:**
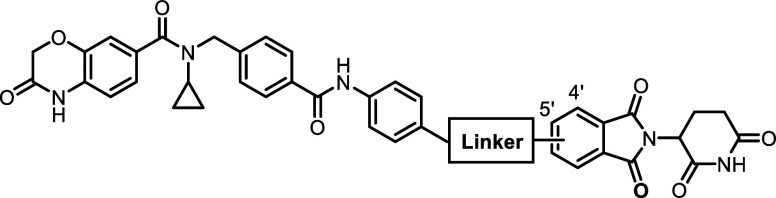

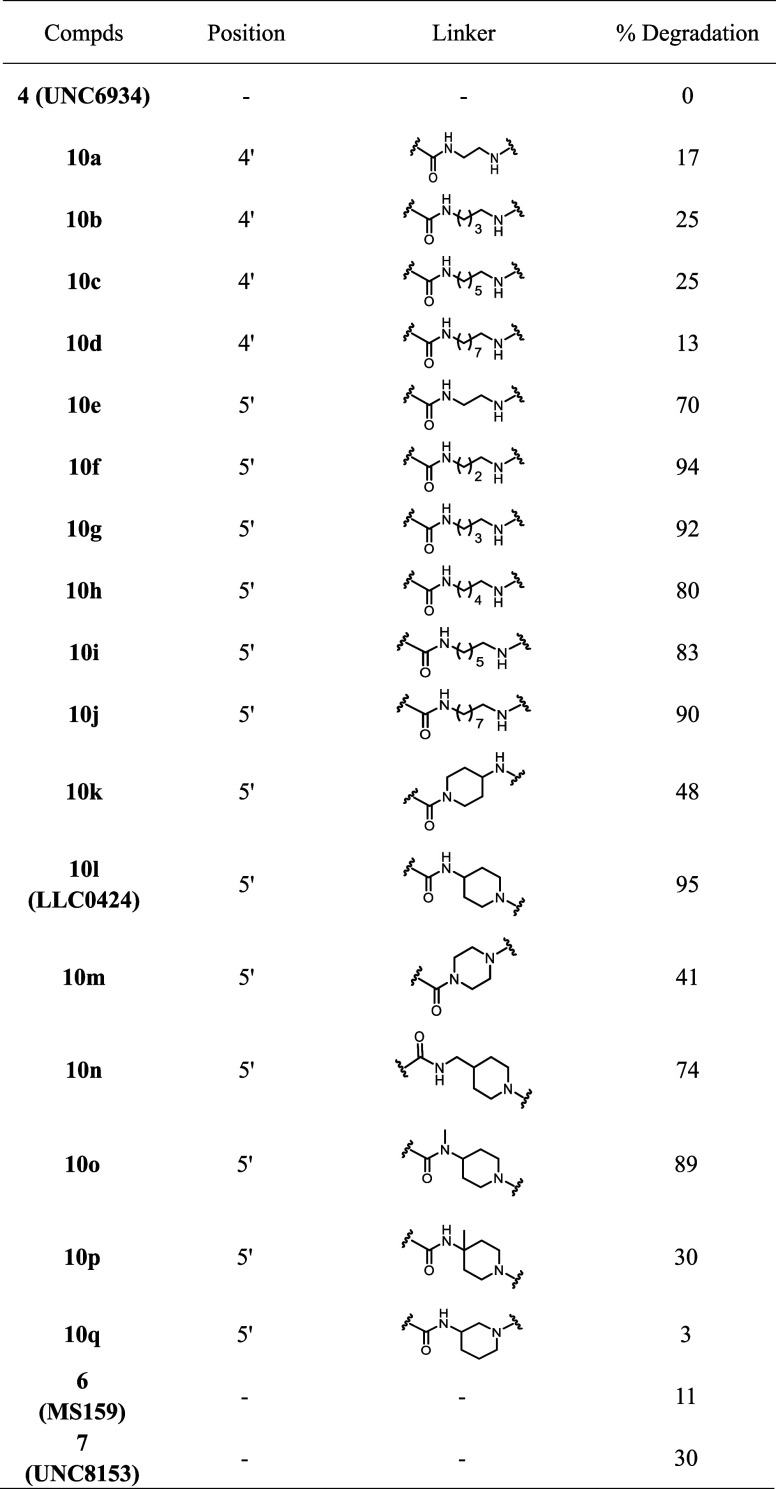
Degradation Efficiency of Compounds **10a**–**q**^a^

a The degradation efficiency was determined by immunoblotting after treatment with compounds at 2 μM in VCaP cells for 24 h.

Studies have shown that introduction of conformationally rigid linkers in PROTAC degraders could improve the degradation potency, selectivity, and pharmacokinetics properties.^24,25^ We next designed and synthesized compounds **10k** and **10l** (**LLC0424**) with rigid linkers of the length similar to that of compound **10f**. The results revealed that while compound **10k** showed significant potency loss compared to compound **10f**, **LLC0424** effectively degraded NSD2 with a degradative rate of 95% at 2 μM (Table 1). Further modification on the linker of **LLC0424** yielded compounds **10m**–**10q**, which were less potent than **LLC0424**. Therefore, **LLC0424** was advanced into further characterization. It is worth noting that the reported NSD2 degraders **6** and **7** at 2 μM showed the degradative rates of 11% and 30% for NSD2, respectively. These results indicated that the linker with the appropriate length and rigid conformation in **LLC0424** may perform better than that of degrader **6** in recruiting the NSD2 and CRBN to form the ternary complex, resulting in better NSD2 degradative activity of **LLC0424**.

### **LLC0424** Induced Degradation of NSD2 Protein in a Concentration- and Time-Dependent Manner

**Figure 3:**
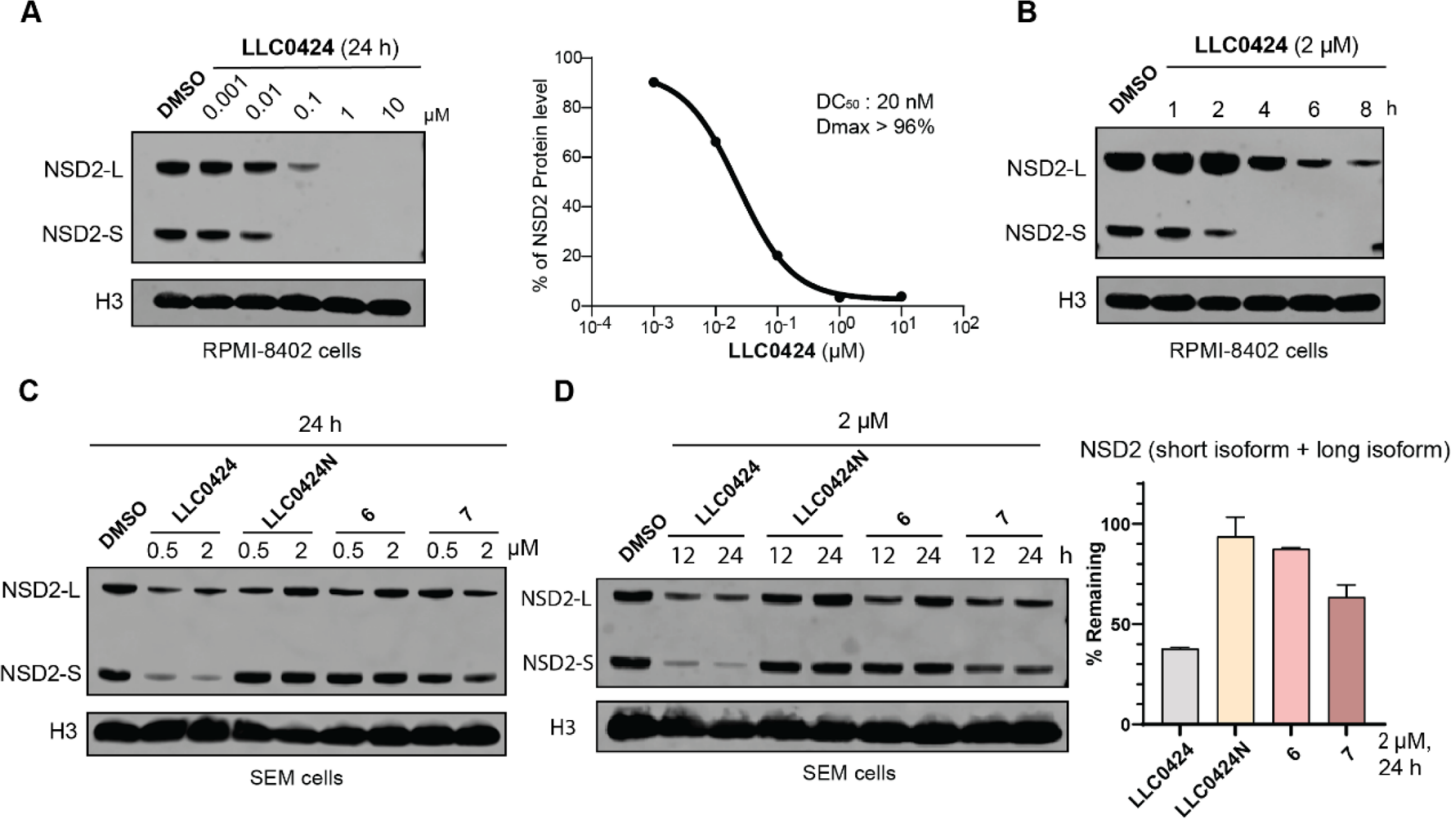
**LLC0424** reduced the protein level of both NSD2 isoforms in a concentration- and time-dependent manner. (A) Immunoblotting of NSD2 (long and short isoforms) and histone3 (H3) in RPMI-8402 cells treated with increasing concentrations of **LLC0424** for 24 h (left), percent remaining NSD2 protein was plotted for DC_50_ and *D*_max_ determination (right). (B) Immunoblotting of NSD2 and H3 in RPMI-8402 cells treated with 2 μM **LLC0424** for various time points. (C) Immunoblotting of NSD2 and H3 in SEM cells treated with 0.5 and 2 μM **LLC0424**, **LLC0424N**, **6**, and **7** for 24 h. (D) Immunoblotting of NSD2 and H3 in SEM cells treated with 2 μM **LLC0424**, **LLC0424N**, **6**, and **7** for 12 and 24 h (left), quantification of percent remaining NSD2 protein after 24 h treatment of 2 μM **LLC0424**, **LLC0424N**, **6**, and **7** (right). The quantification was determined based on two independent experiments. H3 was used as a loading control.

As previous studies have shown that ALL cell lines with NSD2 gain-of-function mutation are more dependent on NSD2 regarding H3K36me2 level and cell proliferation,^7,8^ we further characterized **LLC0424** in ALL cell lines with NSD2 E1099K gain-of-function mutation (RPMI-8402 and SEM). We first treated these cells with **LLC0424** with different concentrations for 24 h. The results revealed that **LLC0424** concentration-dependently induced NSD2 (both long and short isoforms) degradation in RPMI-8402 and SEM cells with DC_50_ values of 20 nM, 110 nM, and *D*_max_ values of >96%, >78%, respectively (Figure 3A and Supporting Information (SI), Figure S1A). We also performed a time-dependent degradation study in RPMI-8402 and SEM cells and found that **LLC0424** started to induce degradation of NSD2 as early as 4 h and led to significant NSD2 degradation within 8 h (Figure 3B and SI, Figure S1B). In addition, we compared the degradation efficiency of **LLC0424** with the two previously published NSD2 degraders **6** and **7** at different concentrations and time points. The results showed that either treatment with **LLC0424** at 0.5 and 2 μM for 24 h or treatment with **LLC0424** at 2 μM for 12 and 24 h caused significant NSD2 degradation in RPMI-8402 and SEM cells, while compounds **6** and **7** as well as the negative control **LLC0424N** (designed by methylating the thalidomide moiety of **LLC0424**, and SI, Scheme S1)^26^ were much less active at the same condition (Figure 3C,D, and SI, Figure S1C–F). These results suggested that **LLC0424** induced NSD2 degradation in a concentration- and time-dependent manner, and significantly outperformed the reported NSD2 degraders **6** and **7**.

### **LLC0424** Induced NSD2 Degradation in a CRBN- and Proteasome-Dependent Manner

**Figure 4:**
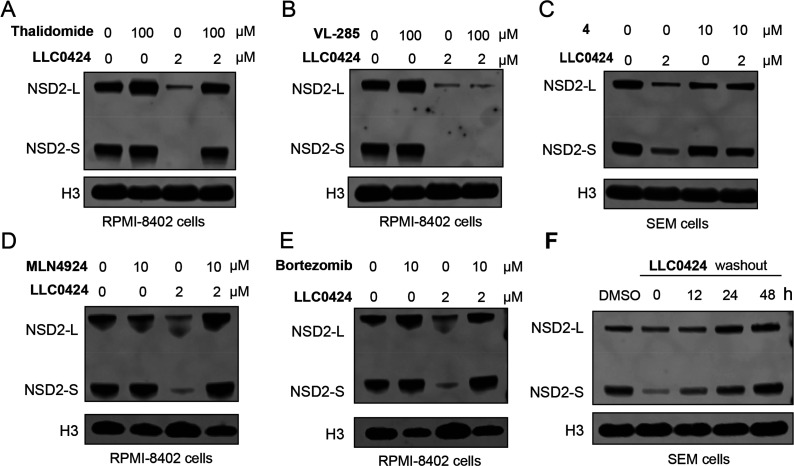
**LLC0424**-mediated NSD2 degradation was CRBN- and proteasome-dependent. Immunoblotting of NSD2 (long and short isoforms) and H3 in RPMI-8402 cells treated with 2 μM **LLC0424** with or without CRBN ligand thalidomide (A), VHL ligand VL285 (B), NEDD8-activating enzyme inhibitor MLN4924 (D), or proteasome inhibitor bortezomib (E). (C) Immunoblotting of NSD2 and H3 in SEM cells treated with 2 μM **LLC0424** with or without the warhead **4**. (F) Immunoblotting of NSD2 and H3 in SEM cells with washout in drug-free medium after 12 h treatment with 2 μM **LLC0424**.

To investigate the mechanism of action of **LLC0424**-mediated NSD2 degradation, we pretreated RPMI-8402 and SEM cells with CRBN ligand thalidomide, von Hippel–Lindau (VHL) ligand VL-285 or the warhead compound **4** for 2 h and then chased with 2 μM **LLC0424** for 12 h. The results showed that pretreatment with thalidomide and compound **4**, but not VL-285, reversed the **LLC0424**-mediated NSD2 degradation (Figure 4A–C, and SI, Figure S2A–C). In addition, pretreatment of RPMI-8402 cells with NEDD8-activating enzyme inhibitor MLN4924 or proteasome inhibitor bortezomib also rescued the NSD2 protein reduction induced by **LLC0424** (Figure 4D,E). These results demonstrated that **LLC0424** induced NSD2 degradation in a CRBN- and proteasome-dependent manner. Next, we performed a washout assay to assess the reversibility of **LLC0424**-mediated NSD2 degradation. Treatment of SEM cells with 2 μM **LLC0424** for 12 h induced significant NSD2 protein degradation. We then used fresh medium to wash out **LLC0424** and found that the NSD2 protein was gradually recovered and fully recovered at 48 h, suggesting that the NSD2 degradation induced by **LLC0424** was a reversible process (Figure 4F).

### **LLC0424** Degraded NSD2 with High Selectivity

**Figure 5:**
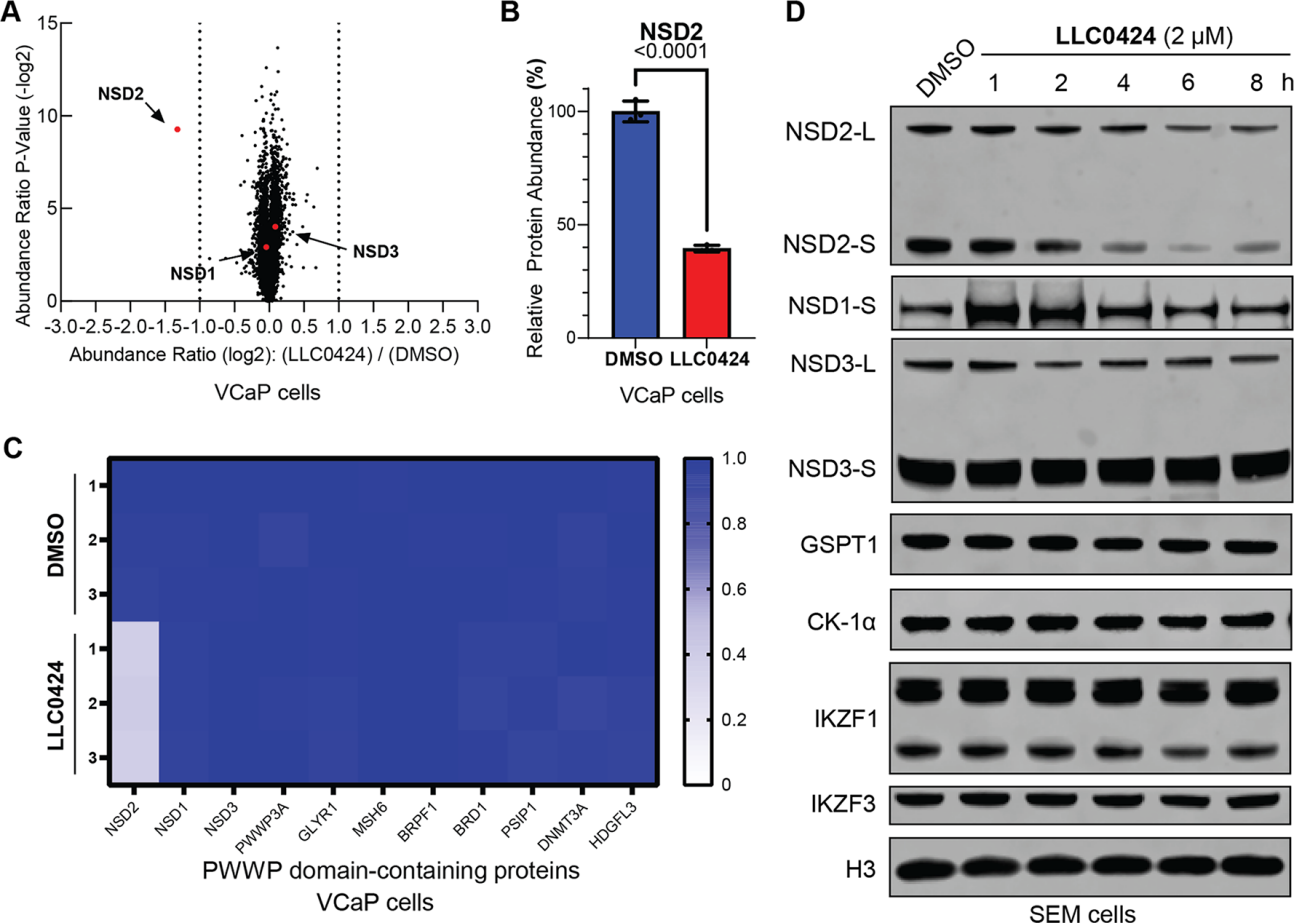
**LLC0424** was a selective NSD2 degrader. (A) Unbiased global proteomics analysis of **LLC0424** in VCaP cells after 6 h treatment of DMSO or 2 μM **LLC0424**. (B) Mass-spec quantification of NSD2 protein. (C) Heatmap for the level of PWWP-1 domain-containing proteins in VCaP cells treated with DMSO or 2 μM **LLC0424**. (D) Immunoblotting of NSD2, NSD1, NSD3, GSPT1, CK-1α, IKZF1, IKZF3, and H3 in SEM cells treated with 2 μM **LLC0424** for various time points.

We conducted a global proteomic assay using tandem mass tags (TMT) labeled mass-spectrometry to unbiasedly quantify the protein change upon **LLC0424** treatment in VCaP and RPMI-8402 cells. The results indicated that **LLC0424** was a very specific NSD2 degrader, with only NSD2 significantly decreased in VCaP cells, and only 3 proteins including NSD2 significantly decreased in RPMI-8402 cells (Figure 5A,B, and SI, Figure S3A,B). It is worth noting that **LLC0424** was selective for NSD2 over other PWWP domain-containing proteins in both cell lines (Figure 5C, and SI, Figure S3C). We further used SEM cell line to cross-validate the selectivity of **LLC0424** for NSD2 over NSD1 and NSD3 as well as CRBN neo-substrates IKZF1, IKZF3, GSPT1, and CK-1α using immunoblotting. The results showed while NSD2 protein started to reduce at 4 h after treatment of SEM cells with 2 μM **LLC0424**, NSD1, NSD3, and CRBN neo-substrates remained unchanged at 8 h (Figure 5D). Notably, although GSPT1 showed significant decrease in global proteomics analysis and immunoblotting in RPMI-8402 cells (SI, Figure S3A,D), treatment of SEM or VCaP cells with **LLC0424** did not induce GSPT1 degradation for as long as 8 h (Figure 5D, and SI, Figure S3E). When we increased the treatment time of **LLC0424** in SEM cells, we observed mild decrease of GSPT1 from 12 h (SI, Figure S3F).

### **LLC0424** Downregulated H3K36me2 Levels and Inhibited the Growth of ALL Cells with NSD2 Mutation

**Figure 6:**
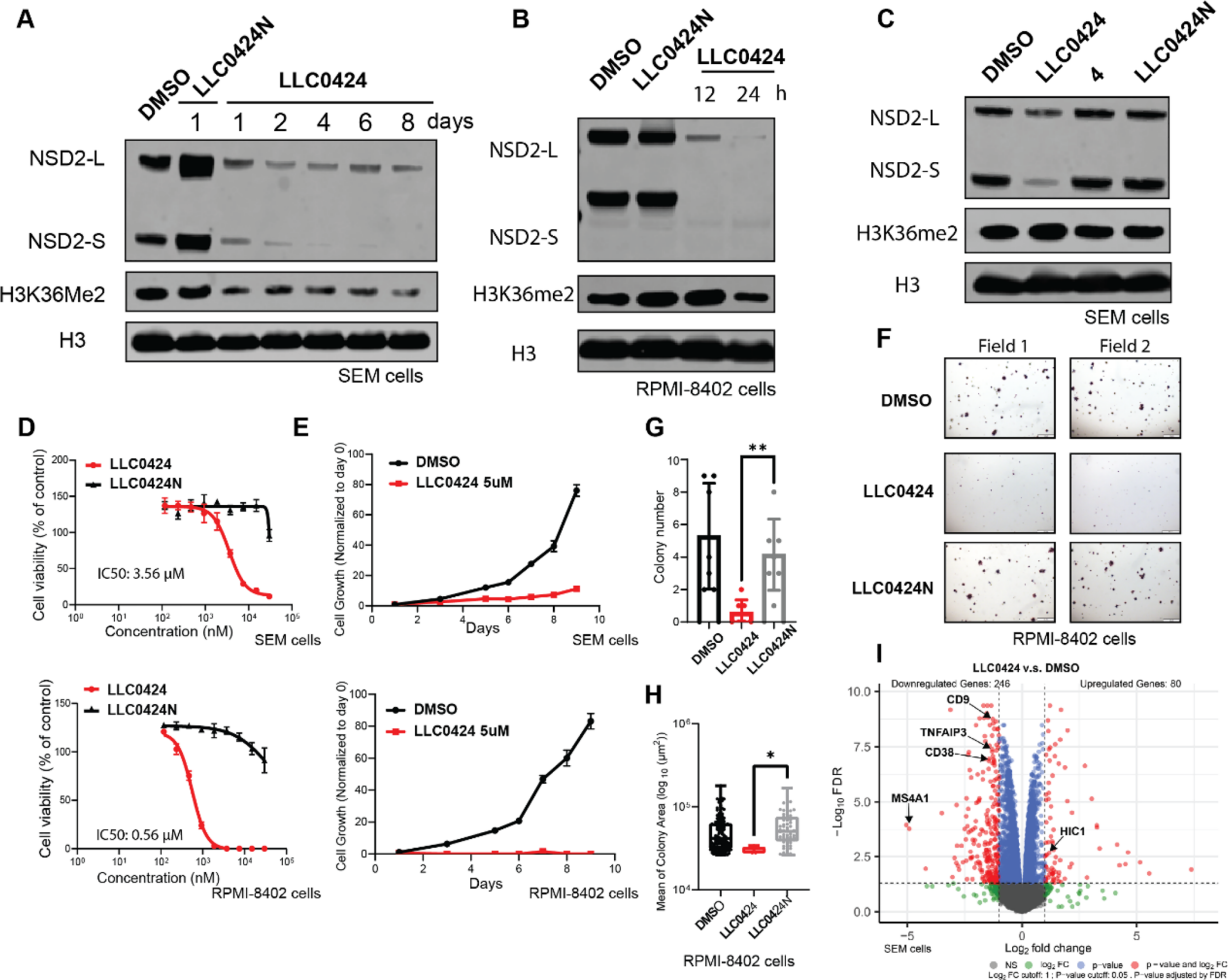
**LLC0424** downregulated H3K36Me2 levels and impeded cell proliferation. (A) Immunoblotting of NSD2, H3K36Me2, and H3 in SEM cells treated with 2 μM **LLC0424** for various time points; DMSO and **LLC0424N** are used as controls. (B) Immunoblotting of NSD2, H3K36Me2, and H3 in RPMI-8402 cells treated with 2 μM **LLC0424** for 12 and 24 h. (C) Immunoblotting of NSD2, H3K36me2 and H3 in SEM cells treated with 2 μM **LLC0424**, **4**, and **LLC0424N** for 8 h. (D) SEM or RPMI-8402 cells were treated with **LLC0424** or **LLC0424N** at varying concentrations for 7 days and quantified for cell viability by CellTiter-Glo assay. Data are reported as the mean of six independent experiments ± SD. (E) SEM or RPMI-8402 cells were treated with 5 μM **LLC0424** or DMSO for 9 days and quantified for cell viability by CellTiter-Glo assay at different days. Data are reported as the mean of five independent experiments ± SD. (F) Iodonitrotetrazolium chloride (INT) staining showing the inhibitory effect of **LLC0424** on colony formation in RPMI-8402 cells. INT staining was conducted at the 30 days of treatment. (G) Quantification of colony numbers of RPMI-8402 cells at 30 days of treatment; (H) Quantification of colony area of RPMI-8402 cells at 30 days of treatment. (I) RNA-seq volcano plot for differentially expressed genes in SEM cells with or without 14 days of **LLC0424** treatment.

NSD2 is the primary methyltransferase that transfers dimethyl to H3K36 in ALL with NSD2 gain-of-function mutation. Therefore, the effects of **LLC0424** on H3K36me2 was evaluated in SEM and RPMI-8402 cells. The results indicated that **LLC0424** treatment led to persistent downregulation of H3K36Me2 level in SEM cells for as long as 8 days (Figure 6A). Meanwhile, **LLC0424** treatment also led to downregulation of H3K36Me2 in RPMI-8402 cells within 24 h (Figure 6B). We next tested the cytotoxic effect of **LLC0424** on SEM and RPMI-8402 cells and found that **LLC0424** dose-dependently suppressed the growth of these two cell lines, with IC_50_ values of 3.56 μM and 0.56 μM respectively, while its negative control **LLC0424N** was much less active (Figure 6C,D). In addition, the growth of SEM and RPMI-8402 cells was also significantly inhibited upon treatment with **LLC0424** for 9 days, which is stronger than the effect of the reported NSD2 degrader **6** and **7** (Figure 6E, and SI, Figure S4A). We also conducted a colony formation assay to evaluate the effect of **LLC0424** on the stem cell-like property of the leukemia cells. It was shown that treatment with **LLC0424** but not **LLC0424N** significantly reduced the formation of cell colonies in RPMI-8402 cells (Figure 6F). Quantification of the colonies showed that RPMI-8402 cells not only formed significantly fewer cell colonies but also had obviously smaller colonies in **LLC0424**-treated group, compared to DMSO or **LLC0424N**-treated group (Figure 6G,H). As **LLC0424** leads to strong degradation of both NSD2-L and NSD2-S, the phenotypic changes upon **LLC0424** treatment result from complete inactivation of NSD2 activity, including adaptor-like function of NSD2-S and SET-domain dependent enzymatic function of NSD2-L. The specific contributions of NSD2 isoforms warrant future investigation. To characterize the genes affected by **LLC0424** treatment, we performed global transcriptome analysis of SEM cells treated by DMSO, **LLC0424**, or **LLC0424N**. **LLC0424** treatment led to significant changes in the transcriptome of SEM cells with over 326 differentially expressed genes. Notably, the down-regulated transcripts include several genes that are associated with increased proliferation and aggressiveness of leukemia namely CD38, MS4A1, CD9, and TNFAIP3 (Figure 6I).^27−30^ Also, CD38 expression has been shown to be activated by NSD2,^27^ and many therapeutics are being actively developed to target this cell surface receptor in both ALL and multiple myeloma.^31,32^ In comparison, **LLC0424N** treatment led to little transcription level changes compared to DMSO treatment in SEM cells (SI, Figure S4B). We further explored the contribution of NSD2 loss to the phenotype we observed in SEM cells. We generated SEM cell line that overexpresses NSD2 (SI, Figure S4C) and found that the cell viability was significantly higher in NSD2-overexpressed SEM cells compared to that in parental SEM cells upon **LLC0424** treatment for 9 days, suggesting that NSD2 degradation contributed to the cytotoxic effect of **LLC0424** in SEM cells (SI, Figure S4D). Still, whether and how **LLC0424**’s modest effect on GSPT1 contributes to the cytotoxic effects remains to be further explored. We also designed and synthesized the negative control **LLC0877** bearing the warhead that is devoid of NSD2 binding (SI, Figure S5A). The results revealed that while **LLC0877** did not degrade NSD2, it showed fast and strong degradation of GSPT1 (SI, Figure S5B). This compound also showed lower IC_50_ values in SEM and RPMI-8402 cells (SI, Figure S5C). Taken together, these data demonstrated that **LLC0424** downregulated H3K36Me2 level and showed an antiproliferative effect on ALL cell lines with NSD2 mutation.

### **LLC0424** Potently Depleted NSD2 *In Vivo*

**Figure 7:**
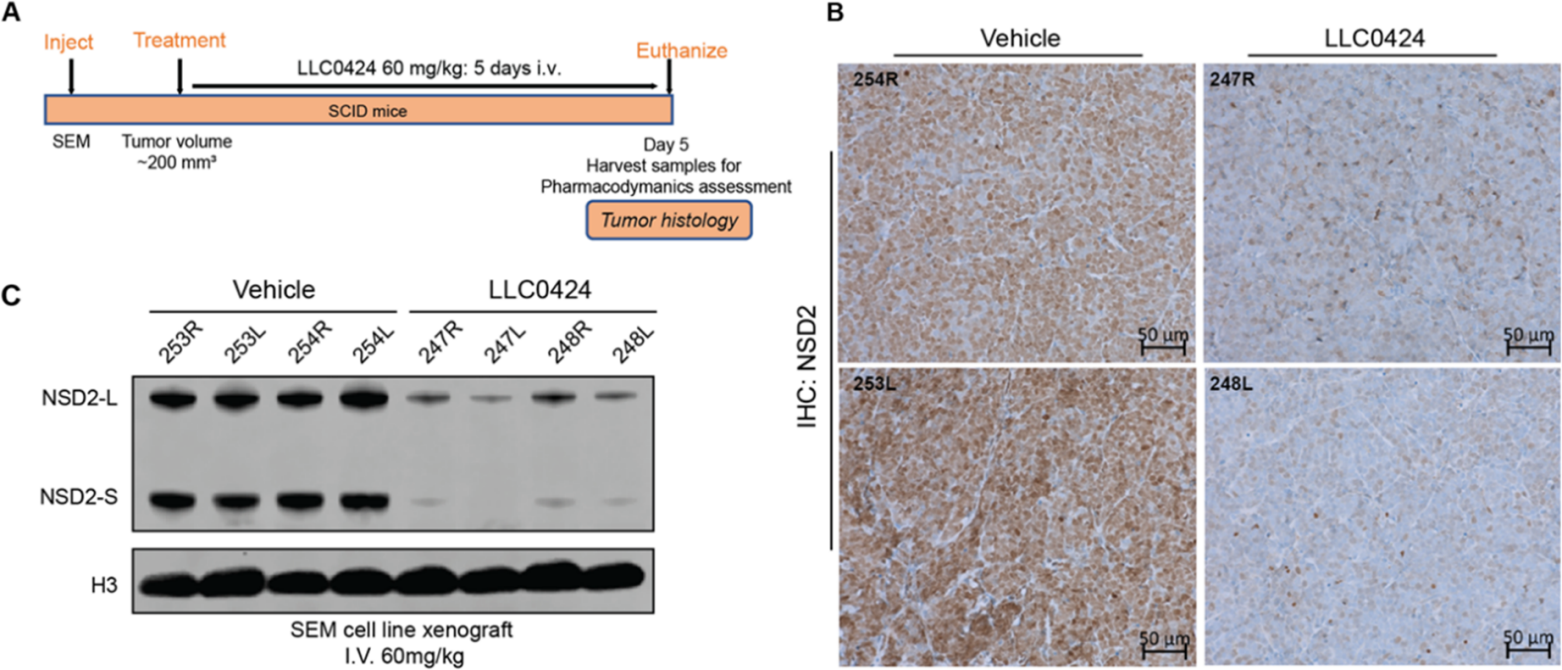
**LLC0424** degraded NSD2 protein *in vivo*. (A) Study design of pharmacodynamics assessment on target engagement of **LLC0424** in the SEM xenograft model. (B) Representative immunohistochemistry images from the SEM xenograft study for NSD2 at two magnifications. (C) Immunoblotting of NSD2 and H3 on tissue lysate of SEM xenografts model after **LLC0424** treatment.

We finally performed pharmacodynamic assessment of **LLC0424** in both the SEM and 22RV1 xenograft model. Based on the solubility of **LLC0424** and the tolerance of the mice, we chose 60 mg/kg as the dosage for the pharmacodynamic study. **LLC0424** was administrated by intravenous (*iv*) or intraperitoneal (*ip*) injection at a dose of 60 mg/kg for 5 consecutive days. The tumor tissues were harvested at day 5 and subjected to histology and Western blot analysis (Figure 7A, and SI, Figure S6A). It was shown that compared to the vehicle treatment group, NSD2 was significantly downregulated following **LLC0424** treatment in both cell line xenografts (Figure 7B,C, and SI, Figure S5B,C), demonstrating its strong NSD2 degradation efficiency *in vivo*. It is worth noting that in 22RV1 xenograft model, both *iv* administration and *ip* administration showed comparable level of NSD2 degradation (SI, Figure S6B,C).

## Conclusion

The histone methyltransferase NSD2 is associated with several types of cancers such as ALL, multiple myeloma, and prostate cancer. However, there has been limited success in potently and selectively targeting the SET or PWWP1 domain of NSD2 with small molecules. While two types of NSD2 degraders have been discovered, their NSD2 degradative activities were relatively low and none of these compounds have been demonstrated to degrade NSD2 *in vivo*. Herein, we report the discovery of a novel NSD2 PROTAC degrader **LLC0424** that potently degraded NSD2 protein with a DC_50_ value of 20 nM and a *D*_max_ value of 96% in RPMI-8402 cells. Mechanistic studies revealed **LLC0424** to selectively induce NSD2 degradation in a CRBN- and proteasome-dependent manner. **LLC0424** also effectively downregulated the level of H3K36me2 and inhibited the growth of ALL cell lines with NSD2 mutation. Importantly, **LLC0424** showed potent NSD2 degradative effects *in vivo*. Overall, **LLC0424** can serve as a valuable tool compound for exploring the roles of NSD2 in physiologic and pathologic conditions.

## Experimental Section

### General Chemistry Methods

The reagents and solvents used in chemical synthesis were obtained from commercial agents without further purification. All reactions were monitored by using thin-layer chromatography (TLC). All final compounds were purified by a column chromatography on silica gel (300–400 mesh). The NMR spectra were recorded on an Agilent DD2 500 MHz, or a Bruker Avance III HD 600 MHz NMR spectrometer in DMSO-*d*_6_. The spectra of high-resolution mass (HRMS) were monitored by Bruker MaXis 4G TOF mass spectrometer and an ESI source. The purities of all final compounds were identified by HPLC analysis with the Agilent 1200 system and were proved to be >95%. HPLC condition: Triart C18 reversed-phase column, 5 μm, 4.6 mm × 250 mm, and flow rate 1.0 mL/min, starting with a 15 min-gradient from 0.1% TFA in water and acetonitrile 1:9 mixture to 0.1% TFA in acetonitrile, then ending with 0.1% TFA in acetonitrile for 5 min.

#### *N*-Cyclopropyl-*N*-(4-((4-((2-((2-(2,6-dioxopiperidin-3-yl)-1,3-dioxoisoindolin-4-yl)amino)ethyl)carbamoyl)phenyl)carbamoyl)benzyl)-3-oxo-3,4-dihydro-2*H*-benzo[*b*][1,4]oxazine-7-carboxamide (**10a**)

*tert*-Butyl (2-((2-(2,6-dioxopiperidin-3-yl)-1,3-dioxoisoindolin-4-yl)amino)ethyl)carbamate (40.0 mg, 0.096 mmol) was dissolved in 2.0 mL of CH_2_Cl_2_. To above solution was added TFA (0.5 mL) by syringe. The mixture was stirred at room temperature for 2 h. After the reaction was complete, the solvent was removed by vacuum. The crude product was dissolved in a small amount of DMF (2.0 mL) and were added 4-(4-((*N*-cyclopropyl-3-oxo-3,4-dihydro-2*H*-benzo[*b*][1,4]oxazine-7-carboxamido)methyl)benzamido)benzoic acid (51.3 mg, 0.106 mmol), triethylamine (29.2 mg, 40 μL, 0.288 mmol), and HATU (54.8 mg, 0.144 mmol). The mixture was stirred at room temperature for 3 h. The resulting mixture was purified by column chromatography to afford the title compound as a yellow solid (66.5 mg, 88% yield). ^1^H NMR (600 MHz, DMSO-*d*_6_) δ 11.09 (s, 1H), 10.90 (s, 1H), 10.47 (s, 1H), 8.65 (t, 1H), 7.97 (d, *J* = 8.1 Hz, 2H), 7.90–7.83 (m, 4H), 7.61–7.57 (m, 1H), 7.46 (d, *J* = 7.3 Hz, 2H), 7.27 (d, *J* = 8.7 Hz, 1H), 7.19 (d, *J* = 8.1 Hz, 1H), 7.15 (s, 1H), 7.03 (d, *J* = 7.0 Hz, 1H), 6.94 (d, *J* = 8.1 Hz, 1H), 6.85 (t, *J* = 6.0 Hz, 1H), 5.05 (dd, *J* = 12.8, 5.4 Hz, 1H), 4.72 (s, 2H), 4.61 (s, 2H), 3.55–3.50 (m, 2H), 3.48–3.43 (m, 2H), 2.93–2.84 (m, 1H), 2.83–2.77 (m, 1H), 2.63–2.52 (m, 2H), 2.05–1.99 (m, 1H), 0.58–0.52 (m, 2H), 0.51–0.44 (m, 2H). ^13^C NMR (151 MHz, DMSO-*d*_6_) δ 172.8, 170.1, 168.7, 167.3, 166.3, 165.6, 164.8, 146.4, 142.5, 142.4, 141.9, 136.2, 133.4, 132.2, 131.8, 129.1, 128.4, 128.1, 127.9, 127.2, 121.9, 119.4, 117.3, 115.4, 115.2, 110.5, 109.3, 66.7, 48.5, 45.5, 41.4, 40.1, 39.0, 38.7, 31.0, 22.2, 9.5. HRMS (*m*/*z*): [M + Na]^+^ calcd for C_42_H_37_N_7_O_9_Na^+^ 806.2545, found 806.2549. HPLC purity: 99.29%.

Compounds **10b**–**10q** were prepared by a procedure similar to that used for compound **10a.**

#### *N*-Cyclopropyl-*N*-(4-((4-((4-((2-(2,6-dioxopiperidin-3-yl)-1,3-dioxoisoindolin-4-yl)amino)butyl)carbamoyl)phenyl)carbamoyl)benzyl)-3-oxo-3,4-dihydro-2*H*-benzo[*b*][1,4]oxazine-7-carboxamide (**10b**)

Yield, 83%. ^1^H NMR (600 MHz, DMSO-*d*_6_) δ 11.09 (s, 1H), 10.88 (s, 1H), 10.42 (s, 1H), 8.44–8.36 (m, 1H), 7.96 (d, *J* = 7.9 Hz, 2H), 7.88–7.82 (m, 4H), 7.60–7.54 (m, 1H), 7.46 (d, *J* = 7.2 Hz, 2H), 7.19 (d, *J* = 7.9 Hz, 1H), 7.15 (s, 1H), 7.12 (d, *J* = 8.6 Hz, 1H), 7.02 (d, *J* = 7.0 Hz, 1H), 6.93 (d, *J* = 8.1 Hz, 1H), 6.61–6.55 (m, 1H), 5.05 (dd, *J* = 12.8, 5.4 Hz, 1H), 4.72 (s, 2H), 4.62 (s, 2H), 3.37–3.29 (m, 4H), 2.92–2.84 (m, 1H), 2.83–2.76 (m, 1H), 2.62–2.52 (m, 2H), 2.06–1.99 (m, 1H), 1.67–1.56 (m, 4H), 0.59–0.52 (m, 2H), 0.50–0.44 (m, 2H). ^13^C NMR (151 MHz, DMSO-*d*_6_) δ 172.82, 170.11, 168.93, 167.31, 165.63, 165.58, 164.86, 146.42, 142.49, 142.35, 141.70, 136.27, 133.44, 132.22, 131.79, 129.48, 128.39, 128.06, 127.84, 127.22, 121.87, 119.34, 117.25, 115.36, 115.16, 110.39, 109.02, 66.74, 48.53, 45.71, 41.57, 40.06, 38.78, 30.98, 26.61, 26.28, 22.16, 9.54. HRMS (*m*/*z*): [M + Na]^+^ calcd for C_44_H_41_N_7_O_9_Na^+^ 834.2858, found 834.2853. HPLC purity: 96.36%.

#### *N*-Cyclopropyl-*N*-(4-((4-((6-((2-(2,6-dioxopiperidin-3-yl)-1,3-dioxoisoindolin-4-yl)amino)hexyl)carbamoyl)phenyl)carbamoyl)benzyl)-3-oxo-3,4-dihydro-2*H*-benzo[*b*][1,4]oxazine-7-carboxamide (**10c**)

Yield, 81%. ^1^H NMR (500 MHz, DMSO-*d*_6_) δ 11.08 (s, 1H), 10.87 (s, 1H), 10.41 (s, 1H), 8.34 (t, *J* = 5.6 Hz, 1H), 7.95 (d, *J* = 8.2 Hz, 2H), 7.88–7.81 (m, 4H), 7.60–7.56 (m, 1H), 7.46 (d, *J* = 7.9 Hz, 2H), 7.19 (d, *J* = 8.0 Hz, 1H), 7.15 (s, 1H), 7.09 (d, *J* = 8.6 Hz, 1H), 7.02 (d, *J* = 7.0 Hz, 1H), 6.93 (d, *J* = 8.1 Hz, 1H), 6.54 (t, *J* = 6.0 Hz, 1H), 5.05 (dd, *J* = 12.7, 5.4 Hz, 1H), 4.72 (s, 2H), 4.62 (s, 2H), 3.31–3.28 (m, 2H), 3.27–3.23 (m, 2H), 2.92–2.84 (m, 1H), 2.83–2.77 (m, 1H), 2.65–2.52 (m, 2H), 2.04–1.99 (m, 1H), 1.62–1.51 (m, 4H), 1.41–1.34 (m, 4H), 0.58–0.52 (m, 2H), 0.51–0.45 (m, 2H). ^13^C NMR (151 MHz, DMSO-*d*_6_) δ 172.82, 170.12, 168.95, 167.31, 165.58, 165.55, 164.85, 146.43, 142.49, 142.34, 141.66, 136.29, 133.44, 132.21, 131.79, 129.56, 128.39, 128.06, 127.82, 127.22, 121.87, 119.33, 117.19, 115.36, 115.16, 110.37, 109.02, 66.74, 48.53, 45.71, 41.80, 40.06, 30.98, 29.14, 28.66, 26.25, 26.10, 22.15, 9.54. HRMS (*m*/*z*): [M + Na]^+^ calcd for C_46_H_45_N_7_O_9_Na^+^ 862.3171, found 862.3177. HPLC purity: 96.47%.

#### *N*-Cyclopropyl-*N*-(4-((4-((8-((2-(2,6-dioxopiperidin-3-yl)-1,3-dioxoisoindolin-4-yl)amino)octyl)carbamoyl)phenyl)carbamoyl)benzyl)-3-oxo-3,4-dihydro-2*H*-benzo[**b**][1,4]oxazine-7-carboxamide (**10d**)

Yield, 82%. ^1^H NMR (500 MHz, DMSO-*d*_6_) δ 11.06 (s, 1H), 10.87 (s, 1H), 10.41 (s, 1H), 8.33 (t, *J* = 5.6 Hz, 1H), 7.95 (d, *J* = 8.2 Hz, 2H), 7.87–7.81 (m, 4H), 7.57 (dd, *J* = 8.6, 7.1 Hz, 1H), 7.46 (d, *J* = 7.9 Hz, 2H), 7.19 (d, *J* = 8.0 Hz, 1H), 7.15 (s, 1H), 7.09 (d, *J* = 8.6 Hz, 1H), 7.01 (d, *J* = 7.0 Hz, 1H), 6.93 (d, *J* = 8.0 Hz, 1H), 6.52 (t, *J* = 5.9 Hz, 1H), 5.05 (dd, *J* = 12.7, 5.4 Hz, 1H), 4.72 (s, 2H), 4.62 (s, 2H), 3.30–3.26 (m, 2H), 3.26–3.21 (m, 2H), 2.92–2.84 (m, 1H), 2.82–2.77 (m, 1H), 2.64–2.52 (m, 2H), 2.05–1.99 (m, 1H), 1.60–1.55 (m, 2H), 1.54–1.49 (m, 2H), 1.37–1.29 (m, 8H), 0.57–0.52 (m, 2H), 0.50–0.45 (m, 2H). ^13^C NMR (151 MHz, DMSO-*d*_6_) δ 172.82, 170.11, 168.96, 167.31, 165.57, 165.52, 164.85, 146.44, 142.49, 142.34, 141.65, 136.28, 133.44, 132.20, 131.79, 129.56, 128.39, 128.06, 127.81, 127.21, 121.87, 119.33, 117.19, 115.36, 115.15, 110.36, 109.00, 66.74, 48.53, 45.73, 41.83, 40.06, 30.98, 29.16, 28.75, 28.73, 28.68, 26.46, 26.30, 22.15, 9.53. HRMS (*m*/*z*): [M + Na]^+^ calcd for C_48_H_49_N_7_O_9_Na^+^ 890.3484, found 890.3480. HPLC purity: 96.50%.

#### *N*-cyclopropyl-*N*-(4-((4-((2-((2-(2,6-dioxopiperidin-3-yl)-1,3-dioxoisoindolin-5-yl)amino)ethyl)carbamoyl)phenyl)carbamoyl)benzyl)-3-oxo-3,4-dihydro-2*H*-benzo[*b*][1,4]oxazine-7-carboxamide (**10e**)

Yield, 81%. ^1^H NMR (600 MHz, DMSO-*d*_6_) δ 11.06 (s, 1H), 10.88 (s, 1H), 10.44 (s, 1H), 8.57 (t, *J* = 5.5 Hz, 1H), 7.96 (d, *J* = 8.2 Hz, 2H), 7.90–7.83 (m, 4H), 7.58 (d, *J* = 8.3 Hz, 1H), 7.46 (d, *J* = 7.5 Hz, 2H), 7.30 (t, *J* = 5.7 Hz, 1H), 7.19 (d, *J* = 7.7 Hz, 1H), 7.15 (s, 1H), 7.05 (d, *J* = 1.8 Hz, 1H), 6.93 (d, *J* = 8.1 Hz, 2H), 5.04 (dd, *J* = 12.7, 5.5 Hz, 1H), 4.72 (s, 2H), 4.62 (s, 2H), 3.48–3.43 (m, 2H), 3.41–3.37 (m, 2H), 2.91–2.84 (m, 1H), 2.83–2.77 (m, 1H), 2.62–2.51 (m, 2H), 2.03–1.96 (m, 1H), 0.60–0.52 (m, 2H), 0.51–0.43 (m, 2H). ^13^C NMR (151 MHz, DMSO-*d*_6_) δ 172.82, 170.17, 167.67, 167.14, 166.03, 165.61, 164.85, 154.38, 142.49, 142.37, 141.88, 134.28, 133.41, 131.79, 129.15, 128.39, 128.07, 127.91, 127.22, 125.12, 121.87, 119.33, 116.22, 115.36, 115.16, 66.74, 48.63, 41.83, 40.06, 38.27, 30.98, 22.24, 9.55. HRMS (*m*/*z*): [M + Na]^+^ calcd for C_42_H_37_N_7_O_9_Na^+^ 806.2545, found 806.2536. HPLC purity: 95.25%.

#### *N*-Cyclopropyl-*N*-(4-((4-((3-((2-(2,6-dioxopiperidin-3-yl)-1,3-dioxoisoindolin-5-yl)amino)propyl)carbamoyl)phenyl)carbamoyl)benzyl)-3-oxo-3,4-dihydro-2*H*-benzo[*b*][1,4]oxazine-7-carboxamide (**10f**)

Yield, 80%. ^1^H NMR (600 MHz, DMSO-*d*_6_) δ 11.06 (s, 1H), 10.88 (s, 1H), 10.43 (s, 1H), 8.44 (t, *J* = 5.6 Hz, 1H), 7.96 (d, *J* = 8.2 Hz, 2H), 7.88–7.83 (m, 4H), 7.57 (d, *J* = 8.4 Hz, 1H), 7.46 (d, *J* = 7.7 Hz, 2H), 7.22–7.12 (m, 3H), 6.97 (d, *J* = 1.6 Hz, 1H), 6.93 (d, *J* = 8.1 Hz, 1H), 6.87 (dd, *J* = 8.4, 2.0 Hz, 1H), 5.03 (dd, *J* = 12.8, 5.5 Hz, 1H), 4.72 (s, 2H), 4.62 (s, 2H), 3.40–3.36 (m, 2H), 3.27–3.23 (m, 2H), 2.91–2.84 (m, 1H), 2.83–2.75 (m, 1H), 2.62–2.51 (m, 2H), 2.02–1.96 (m, 1H), 1.88–1.82 (m, 2H), 0.58–0.52 (m, 2H), 0.50–0.44 (m, 2H). ^13^C NMR (151 MHz, DMSO-*d*_6_) δ 172.83, 170.18, 167.70, 167.17, 165.84, 165.59, 164.86, 154.41, 142.49, 142.36, 141.75, 134.24, 133.43, 131.79, 129.42, 128.39, 128.07, 127.88, 127.22, 125.10, 121.87, 119.35, 115.98, 115.36, 115.16, 66.74, 48.63, 40.35, 40.06, 37.10, 30.99, 28.35, 22.24, 9.54. HRMS (*m*/*z*): [M + Na]^+^ calcd for C_43_H_39_N_7_O_9_Na^+^ 820.2701, found 820.2699. HPLC purity: 97.03%.

#### *N*-Cyclopropyl-*N*-(4-((4-((4-((2-(2,6-dioxopiperidin-3-yl)-1,3-dioxoisoindolin-5-yl)amino)butyl)carbamoyl)phenyl)carbamoyl)benzyl)-3-oxo-3,4-dihydro-2*H*-benzo[*b*][1,4]oxazine-7-carboxamide (**10g**)

Yield, 83%. ^1^H NMR (600 MHz, DMSO-*d*_6_) δ 11.05 (s, 1H), 10.88 (s, 1H), 10.43 (s, 1H), 8.40 (t, *J* = 5.6 Hz, 1H), 7.96 (d, *J* = 8.2 Hz, 2H), 7.88–7.82 (m, 4H), 7.55 (d, *J* = 8.4 Hz, 1H), 7.46 (d, *J* = 7.7 Hz, 2H), 7.22–7.13 (m, 3H), 6.98–6.95 (m, 1H), 6.93 (d, *J* = 8.1 Hz, 1H), 6.86 (dd, *J* = 8.4, 1.9 Hz, 1H), 5.02 (dd, *J* = 12.8, 5.5 Hz, 1H), 4.72 (s, 2H), 4.62 (s, 2H), 3.30 (d, *J* = 6.2 Hz, 2H), 3.23–3.19 (m, 2H), 2.90–2.84 (m, 1H), 2.83–2.77 (m, 1H), 2.62–2.51 (m, 2H), 2.02–1.96 (m, 1H), 1.67–1.59 (m, 4H), 0.58–0.52 (m, 2H), 0.50–0.45 (m, 2H). ^13^C NMR (151 MHz, DMSO-*d*_6_) δ 172.82, 170.19, 167.71, 167.15, 165.64, 165.57, 164.85, 154.46, 142.49, 142.35, 141.70, 134.22, 133.42, 131.79, 129.49, 128.39, 128.06, 127.83, 127.21, 125.10, 121.87, 119.34, 115.81, 115.36, 115.16, 66.74, 48.61, 42.23, 40.06, 38.77, 30.98, 26.82, 25.75, 22.23, 9.55. HRMS (*m*/*z*): [M + Na]^+^ calcd for C_44_H_41_N_7_O_9_Na^+^ 834.2858, found 834.2861. HPLC purity: 97.92%.

#### *N*-Cyclopropyl-*N*-(4-((4-((5-((2-(2,6-dioxopiperidin-3-yl)-1,3-dioxoisoindolin-5-yl)amino)pentyl)carbamoyl)phenyl)carbamoyl)benzyl)-3-oxo-3,4-dihydro-2*H*-benzo[*b*][1,4]oxazine-7-carboxamide (**10h**)

Yield, 79%. ^1^H NMR (600 MHz, DMSO-*d*_6_) δ 11.05 (s, 1H), 10.88 (s, 1H), 10.42 (s, 1H), 8.37 (t, *J* = 5.6 Hz, 1H), 7.96 (d, *J* = 8.2 Hz, 2H), 7.88–7.82 (m, 4H), 7.56 (d, *J* = 8.4 Hz, 1H), 7.46 (d, *J* = 7.7 Hz, 2H), 7.19 (d, *J* = 7.9 Hz, 1H), 7.15 (s, 1H), 7.12 (t, *J* = 5.3 Hz, 1H), 6.95 (s, 1H), 6.93 (d, *J* = 8.1 Hz, 1H), 6.85 (dd, *J* = 8.4, 1.9 Hz, 1H), 5.02 (dd, *J* = 12.8, 5.5 Hz, 1H), 4.72 (s, 2H), 4.62 (s, 2H), 3.30–3.25 (m, 2H), 3.19–3.14 (m, 2H), 2.91–2.84 (m, 1H), 2.83–2.77 (m, 1H), 2.61–2.51 (m, 2H), 2.02–1.96 (m, 1H), 1.66–1.55 (m, 4H), 1.47–1.39 (m, 2H), 0.58–0.52 (m, 2H), 0.50–0.44 (m, 2H). ^13^C NMR (151 MHz, DMSO-*d*_6_) δ 172.82, 170.19, 167.71, 167.16, 165.59, 165.58, 164.85, 154.47, 142.49, 142.35, 141.67, 134.22, 133.43, 131.79, 129.54, 128.39, 128.06, 127.83, 127.22, 125.11, 121.87, 119.34, 115.79, 115.36, 115.16, 66.74, 48.61, 42.47, 40.06, 39.04, 30.98, 28.97, 27.98, 22.24, 9.56. HRMS (*m*/*z*): [M + Na]^+^ calcd for C_45_H_43_N_7_O_9_Na^+^ 848.3014, found 848.3013. HPLC purity: 96.29%.

#### *N*-Cyclopropyl-*N*-(4-((4-((6-((2-(2,6-dioxopiperidin-3-yl)-1,3-dioxoisoindolin-5-yl)amino)hexyl)carbamoyl)phenyl)carbamoyl)benzyl)-3-oxo-3,4-dihydro-2*H*-benzo[**b**][1,4]oxazine-7-carboxamide (**10i**)

Yield, 80%. ^1^H NMR (600 MHz, DMSO-*d*_6_) δ 11.05 (s, 1H), 10.88 (s, 1H), 10.42 (s, 1H), 8.36 (t, *J* = 5.6 Hz, 1H), 7.96 (d, *J* = 8.2 Hz, 2H), 7.88–7.81 (m, 4H), 7.55 (d, *J* = 8.4 Hz, 1H), 7.46 (d, *J* = 7.7 Hz, 2H), 7.19 (d, *J* = 8.0 Hz, 1H), 7.15 (s, 1H), 7.11 (t, *J* = 5.3 Hz, 1H), 6.94 (s, 1H), 6.93 (d, *J* = 8.1 Hz, 1H), 6.84 (dd, *J* = 8.5, 1.8 Hz, 1H), 5.02 (dd, *J* = 12.8, 5.5 Hz, 1H), 4.72 (s, 2H), 4.62 (s, 2H), 3.29–3.23 (m, 2H), 3.19–3.12 (m, 2H), 2.91–2.82 (m, 1H), 2.83–2.77 (m, 1H), 2.63–2.50 (m, 2H), 2.03–1.95 (m, 1H), 1.63–1.50 (m, 4H), 1.45–1.33 (m, 4H), 0.58–0.52 (m, 2H), 0.50–0.44 (m, 2H). ^13^C NMR (151 MHz, DMSO) δ 172.82, 170.19, 167.71, 167.16, 165.57, 165.56, 164.85, 154.47, 142.49, 142.35, 141.66, 134.21, 133.44, 131.79, 129.56, 128.39, 128.06, 127.82, 127.21, 125.12, 121.87, 119.34, 115.77, 115.36, 115.16, 66.74, 48.61, 42.44, 40.06, 30.98, 29.18, 28.21, 26.30, 22.23, 9.55. HRMS (*m*/*z*): [M + Na]^+^ calcd for C_46_H_45_N_7_O_9_Na^+^ 862.3171, found 862.3165. HPLC purity: 99.43%.

#### *N*-Cyclopropyl-*N*-(4-((4-((8-((2-(2,6-dioxopiperidin-3-yl)-1,3-dioxoisoindolin-5-yl)amino)octyl)carbamoyl)phenyl)carbamoyl)benzyl)-3-oxo-3,4-dihydro-2*H*-benzo[**b**][1,4]oxazine-7-carboxamide (**10j**)

Yield, 78%. ^1^H NMR (600 MHz, DMSO-*d*_6_) δ 11.05 (s, 1H), 10.88 (s, 1H), 10.42 (s, 1H), 8.34 (t, *J* = 5.6 Hz, 1H), 7.98–7.93 (m, 2H), 7.88–7.81 (m, 4H), 7.55 (d, *J* = 8.4 Hz, 1H), 7.46 (d, *J* = 7.7 Hz, 2H), 7.19 (d, *J* = 8.0 Hz, 1H), 7.15 (s, 1H), 7.10 (t, *J* = 5.3 Hz, 1H), 6.95–6.91 (m, 2H), 6.84 (dd, *J* = 8.4, 1.9 Hz, 1H), 5.02 (dd, *J* = 12.8, 5.5 Hz, 1H), 4.72 (s, 2H), 4.62 (s, 2H), 3.28–3.21 (m, 2H), 3.18–3.12 (m, 2H), 2.91–2.82 (m, 1H), 2.83–2.77 (m, 1H), 2.63–2.50 (m, 2H), 2.03–1.95 (m, 1H), 1.61–1.47 (m, 4H), 1.40–1.28 (m, 8H), 0.59–0.52 (m, 2H), 0.50–0.43 (m, 2H). ^13^C NMR (151 MHz, DMSO-*d*_6_) δ 172.82, 170.19, 167.71, 167.15, 165.57, 165.52, 164.85, 154.47, 142.48, 142.34, 141.65, 134.21, 133.44, 131.79, 129.56, 128.39, 128.06, 127.81, 127.21, 125.11, 121.87, 119.33, 115.76, 115.36, 115.15, 66.74, 48.60, 42.48, 40.06, 30.98, 29.18, 28.79, 28.77, 28.24, 26.52, 26.48, 22.23, 9.58. HRMS (*m*/*z*): [M + Na]^+^ calcd for C_48_H_49_N_7_O_9_Na^+^ 890.3484, found 890.3482. HPLC purity: 95.94%.

#### *N*-Cyclopropyl-*N*-(4-((4-(4-((2-(2,6-dioxopiperidin-3-yl)-1,3-dioxoisoindolin-5-yl)amino)piperidine-1-carbonyl)phenyl)carbamoyl)benzyl)-3-oxo-3,4-dihydro-2*H*-benzo[*b*][1,4]oxazine-7-carboxamide (**10k**)

Yield, 67%. ^1^H NMR (600 MHz, DMSO-*d*_6_) δ 11.06 (s, 1H), 10.88 (s, 1H), 10.40 (s, 1H), 7.95 (d, *J* = 8.2 Hz, 2H), 7.86 (d, *J* = 8.5 Hz, 2H), 7.57 (d, *J* = 8.4 Hz, 1H), 7.46 (d, *J* = 7.9 Hz, 2H), 7.43–7.39 (m, 2H), 7.19 (d, *J* = 8.2 Hz, 1H), 7.15 (s, 1H), 7.07 (d, *J* = 7.9 Hz, 1H), 7.04 (d, *J* = 2.1 Hz, 1H), 6.95–6.90 (m, 2H), 5.03 (dd, *J* = 12.8, 5.5 Hz, 1H), 4.72 (s, 2H), 4.62 (s, 2H), 3.89–3.75 (m, 1H), 3.29–2.97 (m, 4H), 2.92–2.83 (m, 1H), 2.83–2.75 (m, 1H), 2.63–2.52 (m, 2H), 2.03–1.93 (m, 3H), 1.49–1.33 (m, 2H), 0.61–0.51 (m, 2H), 0.51–0.41 (m, 2H). ^13^C NMR (151 MHz, DMSO-*d*_6_) δ 172.82, 170.16, 168.91, 167.68, 167.11, 165.59, 164.85, 153.31, 142.48, 142.30, 140.35, 134.33, 133.50, 131.79, 130.95, 128.38, 128.04, 127.64, 127.22, 125.16, 121.86, 119.71, 116.16, 115.35, 115.15, 66.73, 48.63, 48.60, 45.71, 30.98, 22.22, 9.53. HRMS (*m*/*z*): [M + Na]^+^ calcd for C_45_H_41_N_7_O_9_Na^+^ 846.2858, found 846.2852. HPLC purity: 98.23%.

#### *N*-Cyclopropyl-*N*-(4-((4-((1-(2-(2,6-dioxopiperidin-3-yl)-1,3-dioxoisoindolin-5-yl)piperidin-4-yl)carbamoyl)phenyl)carbamoyl)benzyl)-3-oxo-3,4-dihydro-2*H*-benzo[*b*][1,4]oxazine-7-carboxamide (**10l**)

Yield, 74%. ^1^H NMR (600 MHz, DMSO-*d*_6_) δ 11.08 (s, 1H), 10.88 (s, 1H), 10.42 (s, 1H), 8.19 (d, *J* = 7.7 Hz, 1H), 7.95 (d, *J* = 8.2 Hz, 2H), 7.86 (s, 4H), 7.68 (d, *J* = 8.5 Hz, 1H), 7.46 (d, *J* = 7.8 Hz, 2H), 7.38 (d, *J* = 2.3 Hz, 1H), 7.30 (dd, *J* = 8.7, 2.3 Hz, 1H), 7.19 (d, *J* = 8.1 Hz, 1H), 7.15 (s, 1H), 6.93 (d, *J* = 8.1 Hz, 1H), 5.08 (dd, *J* = 12.8, 5.4 Hz, 1H), 4.72 (s, 2H), 4.61 (s, 2H), 4.14–4.11 (m, 1H), 3.19–3.08 (m, 4H), 2.93–2.85 (m, 1H), 2.84–2.75 (m, 1H), 2.63–2.53 (m, 2H), 2.07–1.97 (m, 1H), 1.96–1.84 (m, 2H), 1.67–1.57 (m, 2H), 0.61–0.52 (m, 2H), 0.52–0.43 (m, 2H). ^13^C NMR (151 MHz, DMSO-*d*_6_) δ 172.82, 170.13, 167.64, 166.98, 165.57, 165.04, 164.86, 154.77, 142.49, 142.35, 141.78, 134.11, 133.41, 131.79, 129.38, 128.39, 128.06, 127.99, 127.21, 125.07, 121.87, 119.28, 117.85, 117.62, 115.36, 115.15, 107.99, 66.74, 48.77, 48.60, 46.57, 46.50, 30.99, 30.47, 22.20, 9.54. HRMS (*m*/*z*): [M + Na]^+^ calcd for C_45_H_41_N_7_O_9_Na^+^ 846.2858, found 846.2860. HPLC purity: 99.41%.

#### *N*-Cyclopropyl-*N*-(4-((4-(4-(2-(2,6-dioxopiperidin-3-yl)-1,3-dioxoisoindolin-5-yl)piperazine-1-carbonyl)phenyl)carbamoyl)benzyl)-3-oxo-3,4-dihydro-2*H*-benzo[*b*][1,4]oxazine-7-carboxamide (**10m**)

Yield, 71%. ^1^H NMR (600 MHz, DMSO-*d*_6_) δ 11.08 (s, 1H), 10.88 (s, 1H), 10.43 (s, 1H), 7.96 (d, *J* = 8.2 Hz, 2H), 7.91–7.86 (m, 2H), 7.71 (d, *J* = 8.5 Hz, 1H), 7.50–7.45 (m, 4H), 7.37 (d, *J* = 2.3 Hz, 1H), 7.27 (dd, *J* = 8.7, 2.3 Hz, 1H), 7.19 (d, *J* = 8.0 Hz, 1H), 7.15 (s, 1H), 6.93 (d, *J* = 8.1 Hz, 1H), 5.08 (dd, *J* = 12.8, 5.5 Hz, 1H), 4.72 (s, 2H), 4.62 (s, 2H), 3.82–3.46 (m, 8H), 2.92–2.85 (m, 1H), 2.84–2.74 (m, 1H), 2.62–2.53 (m, 2H), 2.07–1.98 (m, 1H), 0.61–0.52 (m, 2H), 0.52–0.42 (m, 2H). ^13^C NMR (151 MHz, DMSO-*d*_6_) δ 172.80, 170.07, 169.00, 167.51, 166.97, 165.63, 164.85, 154.91, 142.49, 142.33, 140.61, 133.87, 133.49, 131.79, 130.34, 128.39, 128.06, 127.24, 124.97, 121.87, 119.66, 118.63, 117.94, 115.36, 115.16, 108.10, 66.74, 48.79, 46.77, 30.98, 22.17, 9.54. HRMS (*m*/*z*): [M + Na]^+^ calcd for C_44_H_39_N_7_O_9_Na^+^ 832.2701, found 832.2700. HPLC purity: 97.99%.

#### *N*-Cyclopropyl-*N*-(4-((4-(((1-(2-(2,6-dioxopiperidin-3-yl)-1,3-dioxoisoindolin-5-yl)piperidin-4-yl)methyl)carbamoyl)phenyl)carbamoyl)benzyl)-3-oxo-3,4-dihydro-2*H*-benzo[*b*][1,4]oxazine-7-carboxamide (**10n**)

Yield, 69%. ^1^H NMR (600 MHz, DMSO-*d*_6_) δ 11.07 (s, 1H), 10.88 (s, 1H), 10.43 (s, 1H), 8.44 (t, *J* = 5.7 Hz, 1H), 7.96 (d, *J* = 8.2 Hz, 2H), 7.87 (s, 4H), 7.65 (d, *J* = 8.6 Hz, 1H), 7.46 (d, *J* = 7.7 Hz, 2H), 7.32 (d, *J* = 1.9 Hz, 1H), 7.24 (dd, *J* = 8.7, 2.2 Hz, 1H), 7.19 (d, *J* = 7.9 Hz, 1H), 7.15 (s, 1H), 6.93 (d, *J* = 8.1 Hz, 1H), 5.06 (dd, *J* = 12.8, 5.5 Hz, 1H), 4.72 (s, 2H), 4.62 (s, 2H), 4.14–3.99 (m, 2H), 3.21–3.16 (m, 2H), 3.00–2.93 (m, 2H), 2.92–2.84 (m, 1H), 2.84–2.76 (m, 1H), 2.62–2.52 (m, 2H), 2.05–1.98 (m, 1H), 1.93–1.84 (m, 1H), 1.83–1.74 (m, 2H), 1.30–1.19 (m, 2H), 0.62–0.52 (m, 2H), 0.51–0.41 (m, 2H). ^13^C NMR (151 MHz, DMSO-*d*_6_) δ 172.81, 170.12, 167.63, 166.97, 165.82, 165.58, 164.85, 154.98, 142.49, 142.36, 141.73, 134.05, 133.42, 131.79, 129.45, 128.39, 128.06, 127.89, 127.22, 125.00, 121.87, 119.34, 117.66, 117.42, 115.36, 115.15, 107.80, 66.74, 48.73, 47.19, 44.47, 35.76, 30.98, 28.94, 22.19, 9.54. HRMS (*m*/*z*): [M + H]^+^ calcd for C_46_H_44_N_7_O_9_^+^ 838.3195, found 838.3192. HPLC purity: 95.95%.

#### *N*-Cyclopropyl-*N*-(4-((4-((1-(2-(2,6-dioxopiperidin-3-yl)-1,3-dioxoisoindolin-5-yl)piperidin-4-yl)(methyl)carbamoyl)phenyl)carbamoyl)benzyl)-3-oxo-3,4-dihydro-2*H*-benzo[*b*][1,4]oxazine-7-carboxamide (**10o**)

Yield, 73%. ^1^H NMR (600 MHz, DMSO-*d*_6_) δ 11.08 (s, 1H), 10.88 (s, 1H), 10.40 (s, 1H), 7.95 (d, *J* = 8.2 Hz, 2H), 7.86 (d, *J* = 8.3 Hz, 2H), 7.67 (d, *J* = 8.5 Hz, 1H), 7.46 (d, *J* = 7.7 Hz, 2H), 7.42 (d, *J* = 8.5 Hz, 2H), 7.35 (s, 1H), 7.31–7.24 (m, 1H), 7.19 (d, *J* = 8.0 Hz, 1H), 7.15 (s, 1H), 6.93 (d, *J* = 8.1 Hz, 1H), 5.07 (dd, *J* = 12.8, 5.4 Hz, 1H), 4.72 (s, 2H), 4.62 (s, 2H), 4.27–4.06 (m, 2H), 3.35–3.28 (m, 2H), 3.14–3.06 (m, 1H), 2.93–2.85 (m, 1H), 2.84–2.74 (m, 4H), 2.62–2.52 (m, 2H), 2.06–1.98 (m, 1H), 1.90–1.79 (m, 2H), 1.79–1.70 (m, 2H), 0.61–0.52 (m, 2H), 0.51–0.42 (m, 2H). ^13^C NMR (151 MHz, DMSO-*d*_6_) δ 172.81, 170.11, 167.59, 166.94, 165.61, 164.85, 142.48, 142.28, 140.18, 134.07, 133.55, 131.84, 131.79, 128.38, 128.04, 127.22, 125.03, 121.87, 119.66, 117.82, 115.36, 115.15, 107.97, 66.74, 64.92, 54.92, 48.74, 48.60, 46.73, 30.98, 22.18, 15.17, 9.55. HRMS (*m*/*z*): [M + H]^+^ calcd for C_46_H_44_N_7_O_9_^+^ 838.3195, found 838.3181. HPLC purity: 96.06%.

#### *N*-Cyclopropyl-*N*-(4-((4-((1-(2-(2,6-dioxopiperidin-3-yl)-1,3-dioxoisoindolin-5-yl)-4-methylpiperidin-4-yl)carbamoyl)phenyl)carbamoyl)benzyl)-3-oxo-3,4-dihydro-2*H*-benzo[*b*][1,4]oxazine-7-carboxamide (**10p**)

Yield, 77%. ^1^H NMR (600 MHz, DMSO-*d*_6_) δ 11.07 (s, 1H), 10.88 (s, 1H), 10.41 (s, 1H), 7.96 (d, *J* = 8.3 Hz, 2H), 7.85 (s, 4H), 7.73 (s, 1H), 7.66 (d, *J* = 8.6 Hz, 1H), 7.46 (d, *J* = 7.7 Hz, 2H), 7.37 (d, *J* = 2.0 Hz, 1H), 7.27 (dd, *J* = 8.7, 2.2 Hz, 1H), 7.19 (d, *J* = 8.0 Hz, 1H), 7.15 (s, 1H), 6.93 (d, *J* = 8.1 Hz, 1H), 5.07 (dd, *J* = 12.8, 5.5 Hz, 1H), 4.72 (s, 2H), 4.62 (s, 2H), 3.81–3.73 (m, 2H), 3.32–3.28 (m, 2H), 2.92–2.84 (m, 1H), 2.84–2.74 (m, 1H), 2.62–2.51 (m, 2H), 2.46–2.40 (m, 2H), 2.05–1.98 (m, 1H), 1.66–1.58 (m, 2H), 1.42 (s, 3H), 0.59–0.51 (m, 2H), 0.51–0.43 (m, 2H). ^13^C NMR (151 MHz, DMSO-*d*_6_) δ 172.82, 170.12, 167.66, 166.99, 166.40, 165.54, 164.85, 154.89, 142.49, 142.34, 141.63, 134.02, 133.42, 131.79, 130.61, 128.39, 128.15, 128.07, 127.21, 124.98, 121.87, 119.20, 117.50, 117.44, 115.36, 115.16, 107.77, 66.74, 51.39, 48.74, 43.62, 34.51, 30.99, 25.81, 22.20, 9.49. HRMS (*m*/*z*): [M + H]^+^ calcd for C_46_H_44_N_7_O_9_^+^ 838.3195, found 838.3197. HPLC purity: 96.69%.

#### *N*-Cyclopropyl-*N*-(4-((4-((1-(2-(2,6-dioxopiperidin-3-yl)-1,3-dioxoisoindolin-5-yl)piperidin-3-yl)carbamoyl)phenyl)carbamoyl)benzyl)-3-oxo-3,4-dihydro-2*H*-benzo[*b*][1,4]oxazine-7-carboxamide (**10q**)

Yield, 81%. ^1^H NMR (600 MHz, DMSO-*d*_6_) δ 11.08 (s, 1H), 10.88 (s, 1H), 10.44 (s, 1H), 8.31 (d, *J* = 7.2 Hz, 1H), 7.96 (d, *J* = 8.2 Hz, 2H), 7.91–7.83 (m, 4H), 7.68 (d, *J* = 8.5 Hz, 1H), 7.46 (d, *J* = 7.5 Hz, 2H), 7.37 (d, *J* = 2.1 Hz, 1H), 7.32–7.27 (m, 1H), 7.19 (d, *J* = 7.9 Hz, 1H), 7.15 (s, 1H), 6.93 (d, *J* = 8.1 Hz, 1H), 5.07 (dd, *J* = 12.8, 5.4 Hz, 1H), 4.72 (s, 2H), 4.62 (s, 2H), 4.14–4.07 (m, 1H), 4.06–3.99 (m, 1H), 3.96–3.87 (m, 1H), 3.09–2.97 (m, 2H), 2.93–2.84 (m, 1H), 2.84–2.76 (m, 1H), 2.62–2.51 (m, 2H), 2.05–1.94 (m, 2H), 1.89–1.83 (m, 1H), 1.77–1.69 (m, 1H), 1.63–1.54 (m, 1H), 0.60–0.52 (m, 2H), 0.51–0.43 (m, 2H). ^13^C NMR (151 MHz, DMSO-*d*_6_) δ 174.08, 173.27, 172.82, 170.11, 167.57, 166.93, 165.70, 165.60, 164.86, 154.58, 142.49, 141.90, 134.14, 133.41, 131.79, 129.14, 128.39, 128.14, 128.07, 127.22, 125.14, 121.87, 119.25, 117.61, 115.36, 115.16, 107.82, 91.00, 83.35, 66.74, 52.33, 28.29, 26.22, 23.28, 9.51. HRMS (*m*/*z*): [M + H]^+^ calcd for C_45_H_42_N_7_O_9_^+^ 824.3039, found 824.3020. HPLC purity: 96.45%.

### Cell Lines, Antibodies, and Compounds

Most cell lines were originally obtained from ATCC or internal stock. All cells were genotyped to confirm their identity at the University of Michigan Sequencing Core and tested routinely for Mycoplasma contamination. SEM was grown in Gibco RPMI-1640 Glutamax + 10% FBS (ThermoFisher) + 1% Gibco MEM NEAA (100 × ) + 1% Gibco sodium pyruvate (100 mM) + 0.1% 2-mercaptoethanol (Sigma-Aldrich). RPMI-8402 was grown in Gibco RPMI-1640 + 10% FBS (ThermoFisher). VCaP was grown in Gibco DMEM + 10% FBS (ThermoFisher). 22RV1 was grown in Gibco RPMI-1640 + 10% FBS (ThermoFisher). Human peripheral blood mononuclear cells were obtained from Lonza. Sources of all antibodies and compounds are listed in SI, Tables S1 and S2.

### Western Blot

After treatment, the cultured cells were collected and rinsed with phosphate-buffered saline (PBS) in room temperature. The cells were treated with RIPA buffers (ThermoFisher Scientific) supplemented with Halt Protease and Phosphatase Inhibitor Cocktail (ThermoFisher Scientific). After sonication and centrifugation, the total amount of protein in the cell lysates was measured by Pierce Bovine Serum Albumin Standard Pre-Diluted Set (ThermoFisher Scientific). After mixed with LDS-sample buffer (Invitrogen) supplemented with Sample Reducing Agent (Invitrogen) and heated in 70 °C for 10 min, equal amount of protein was loaded into NuPAGE 4–12%, Bis-Tris Protein Gel (ThermoFisher Scientific) or NuPAGE 3–8%, Tris-Acetate Protein Gel (ThermoFisher Scientific), and blotted with primary antibodies. After incubated with HRP-conjugated secondary antibodies, the membranes were developed with ECL Prime Western Blotting Detection Reagent (cytiva) and imaged on Odyssey Fc Imager (LiCOR Biosciences).

### Compound Screening

After the compounds were synthesized, they were diluted in DMSO. 1.5 million VCaP cells per well were plated into 6-well plates and we separately treated each well with each compound at 2 μM for 24 h. Western blot was then performed as previously described to detect NSD2 degradation.

### Validation of Mechanism of Action of **LLC0424**

Three million SEM or RPMI-8402 cells per well were plated into 6-well plates and were separately pretreated with thalidomide, VL-285, bortezomib, MLN4924, or UNC-6934 for 2 h. The cells were then treated with LLC0424 12 or 6 h. Western blot was then conducted as previously described to probe NSD2 degradation.

### TMT Proteomics Assay

RPMI-8402 cells were plated at 10 × 10^6^ cells per plate on 10 cm tissue culture plates overnight before treating with DMSO (control) or 2 μM **LLC0424** with 3 replicates for each treatment. After 8 h, whole cell lysates were collected using RIPA buffer (ThermoFisher Scientific) and quantified using Pierce Bovine Serum Albumin Standard Pre-Diluted Set (ThermoFisher Scientific). Cell lysates were proteolyzed and labeled with TMT 6-plex Isobaric Label Reagent (ThermoFisher Scientific) according to the protocol from the manufacturer and subjected to 8 fractions of liquid chromatography–mass spectrometry (LC-MS)/MS analysis as described.^33^ We plotted the results using graphpad prism. To gain protein changes with high confidence, we screened the results of (LC-MS)/MS analysis with the criteria of peptides number >4 and unique peptides number >1.

### Cell Viability Assay

Cells were plated in 50 μL of the corresponding media in 96-well plates. A 2-fold dilution series of 2* concentrated **LLC0424** was prepared in a medium with the highest concentration being 60 μM. Then 50 μL of the 2× compound-containing mixture was added to each well with 6 replicates for each concentration, reaching 1× concentration, with the highest concentration being 30 μM. The plate was incubated for 7 days. At the end point, the CellTiter-Glo assay (Promega) was then performed according to the manufacturer’s instruction to determine cell proliferation. The luminescence signal intensity of each well was acquired on an Infinite M1000Pro microplate reader and the data was analyzed using GraphPad Prism software (GraphPad Software).

### Proliferation Assay

2 × 10^3^ cells per well were seeded in 5 replicates into 96-well plates and treated with 5 μM of the compound. Every 4 days, we added the compound with 5 μL of the 20* compound-containing mixture, reaching 1*concentration. Cell proliferation was evaluated by CellTiter-Glo assay (Promega).

### Colony Formation

Colony formation in soft agar was conducted in 12-well plates, with the bottom layer containing 750 μL of 0.8% agarose in 10% FBS/cell media and the top layer containing 0.4% agarose (SEM cells or RPMI-8402 cells). **LLC0424** was added every 3 days to the liquid layer on top agarose to reach a final concentration of 2 μM. At the 30 days of treatment, the cells were stained with iodonitrotetrazolium chloride (INT) by adding 500 μL 1× INT PBS solution into each well. After the staining, images were taken using brightfield microscope and plate scan was conducted using Epson Perfection V700 Photo. Colony number counting was performed using ImageJ.

### RNA-seq and Analysis

Total RNA was isolated from cells using the miRNeasy Mini Kit (Qiagen). RiboErase: we generated RNA-seq libraries with 200–1000 ng of total RNA. We depleted Ribosomal RNA by enzymatic digestion using the specific probe-bound duplex rRNA (KAPA RNA Hyper+RiboErase HMR, Roche), followed by fragmentation to approximately 200–300 bp by heating in the fragmentation buffer. Next, double-stranded cDNA was prepared, and end-repair and ligation was conducted with New England Biolabs (NEB) adapters. Final library was prepared by amplification with the NEB dual barcode and 2× KAPA HiFi HotStart mix following the manufacturers’ protocols. We used an Agilent 2100 Bioanalyzer to assess library quality in terms of product size and concentration. Paired-end libraries were sequenced with the Illumina HiSeq 2500 (2 × 100 nucleotide read length) with sequence coverage to 15–20 M paired reads. We first processed the RNA data using Kallisto (version 0.46.1). Then we performed the analysis in R. First, EdgeR (edgeR_3.39.6) was used to perform read counts normalization and filtering (counts >10) and then Limma-Voom (limma_3.53.10) was used to analyze differential expression.

### Lentivirus Transduction of SEM Cell Line

The plasmid used in this study is pMSCV-HA-NSD2-FL. Lentivirus particles were generated in collaboration with the Vector Core at the University of Michigan. Following lentiviral production, SEM cells were seeded in a 6-well plate at the density of 1,000,000 cells per well and infected with the virus particles followed by spinoculation at 2500 rpm for 30 min and incubation for 24 h. This was followed by 14 days of blasticidin selection at 5 μg/mL. Overexpression of proteins was validated by immunoblotting.

### **LLC0424** Formula for *In Vivo* Studies

**LLC0424** was first dissolved in DMSO. Then the solution was mixed with 40% of 2-hydroxypropyl-β-cyclodextrin (HPβCD) dissolved with 5% dextrose in water (D5W) and sonicated until completely dissolved. **LLC0424** was freshly prepared right before administration to mice. **LLC0424** was delivered to mice by intravenous injection through the retro-orbital vein.

### Human Tumor Xenograft Models

First, 6–8 weeks old male CB17 severe combined immunodeficiency (SCID) mice were procured from the University of Michigan breeding colony. Subcutaneous tumors were established at both sides of the dorsal flank of mice. Tumors were measured at least biweekly using digital calipers following the formula (π/6) (*L* × *W*2), where *L* is length and *W* is width of the tumor. At the end of the studies, mice were sacrificed and tumors extracted. For each tumor, a part is paraffin fixed followed by embedding in paraffin to make tissue blocks. The blocks were sectioned at 4 μM for immunohistochemistry analysis. Another part is made into tissue lysates for Western blotting analysis. The University of Michigan Institutional Animal Care and Use Committee (IACUC) approved all *in vivo* studies.

For the SEM tumor model, 10 × 10^6^ SEM cells were injected subcutaneously into the dorsal flank on both sides of the mice in a serum-free medium with 50% Matrigel (BD Biosciences). Once tumors reached a palpable stage (∼200 mm^3^), mice were treated with either 60 mg/kg **LLC0424** or vehicle through intravenous injection for five consecutive days.

For the 22RV1 tumor model, 2 × 10^6^ 22RV1 cells were injected subcutaneously into the dorsal flank on both sides of the mice in a serum-free medium with 50% Matrigel (BD Biosciences). Once tumors reached a palpable stage (∼100 mm^3^), mice were treated with either 60 mg/kg **LLC0424** or vehicle through intravenous injection or intraperitoneal injection for five consecutive days. Following the IACUC guidelines, in all treatment arms the maximal tumor size did not exceed the 2.0 cm limit in any dimension.

### Immunohistochemistry

Immunohistochemistry was performed on formalin-fixed paraffin-embedded 4 μm sections of mouse or xenograft tissues. Slides with tissue sections were deparaffinized in xylene, followed by serial hydration steps in ethanol (100%, 70%) and water for 5 min each. Endogenous tissue peroxidase activity was blocked by placing slides in 3% H_2_O_2_–methanol solution for 1 h at room temperature. Antigen retrieval was performed by microwaving slides in a solution of citrate buffer (pH 6) for 15 min, followed by blocking in 2.5% normal horse serum (Vector Laboratories, cat. no. S-2012-50) for 2 h. The slides were then incubated in the following primary antibodies overnight at 4 °C: NSD2 (Abcam cat. no. 75359, 1:300). The slides were washed with PBST on the next day and incubated with secondary antibody (ImmPRESS HRP Universal Antibody Anti-Mouse IgG/Anti-Rabbit IgG, Vector Laboratories, cat. no. MP-7500-50) under room temperature for 1 h. Visualization of staining was done with the DAB Substrate Kit (Vector Laboratories, cat. no. SK-4100) following the manufacturer’s protocol. Following DAB staining, slides were dehydrated in ethanol, xylene (5 min each), and mounted using EcoMount (Thermo Fisher, cat. no. EM897L).

## Supporting Information Available

Degradation profiles of NSD2 by **LLC0424** in various cancer cells; additional mechanism of action and selectivity data of **LLC0424**; Cell antiproliferation data of **LLC0424**, **6** and **7**; degradation and cytotoxicity profile of **LLC0877**; *in vivo* NSD2 degradation effects of **LLC0424** in 22RV1 subcutaneous xenograft model; sources of antibodies and compounds; primer sequences of real-time PCR; PK parameters of **LLC0424** in rats; synthesis of **LLC0424N** and **LLC0877**; ^1^H NMR and ^13^C NMR spectra and HPLC traces for all degraders (PDF)
Molecular formula strings (CSV) (CSV)
The data set of TMT proteomics in RPMI-8402 and VCaP cells (XLSX)

## Abbreviations Used

ALL: acute lymphoblastic leukemia
ATCC: American Type Culture Collection
Boc: *t*-butyloxy carbonyl
CRBN: cereblon
CH_2_Cl_2_: dichloromethane
CK-1α: casein kinase 1α
DC_50_: the half-maximal degradation concentration
*D*_max_: the maximal degradation rate
DMF: *N*,*N*-dimethylformamide
DMSO: dimethyl sulfoxide
DEL: DNA-encoded library
Et_3_N: triethylamine
FBS: fetal bovine serum
GSPT1: G1 To S phase transition 1
H3K36me2: dimethylation of histone 3 lysine36
HRMS: high-resolution mass
HPLC: high performance liquid chromatography
HATU: *N*-[(dimethylamino)-3-oxo-1*H*-1,2,3-triazolo[4,5-*b*]pyridin-1-yl-methylene]-*N*-methylmethanaminium hexafluorophosphate
H3: histone 3
HPβCD: 2-hydroxypropyl-β-cyclodextrin
IKZF: Ikaros family zinc finger
INT: iodonitrotetrazolium chloride
LC-MS: liquid chromatography–mass spectrometry
MMSET: multiple myeloma SET domain
NSD: nuclear receptor-binding SET domain-containing
NMR: nuclear magnetic resonance
PWWP: proline–tryptophan–tryptophan–proline
PHD: plant homeodomain
PROTAC: proteolysis targeting chimera
PDB: Protein Data Bank
PBS: phosphate-buffered saline
rt: room temperature
SET: Su(var.)3–9,enhancerofzeste, and Trithorax
TFA: trifluoroacetic acid
TMT: tandem mass tags
TLC: thin-layer chromatography
VHL: von Hippel–Lindau
WHSC1: Wolf–Hirschhorn syndrome candidate 1
