# Supplementary material for: Discovery of LLC0424 as a Potent and Selective in Vivo NSD2 PROTAC Degrader
Source: J Med Chem. 2024 Apr 30;67(9):6938–51. doi: 10.1021/acs.jmedchem.3c01765 (PMC11094793; doi:10.1021/acs.jmedchem.3c01765)
Supplement: Supplementary file 1 — jm3c01765_si_001.pdf [file jm3c01765_si_001.pdf]

Supporting Information

Discovery of LLC0424 as a Potent and Selective *In Vivo* NSD2 PROTAC Degrader

Lianchao Liu[1,#], Abhijit Parolia[2,3,4,5,#], Yihan Liu[2,6,#], Caiyun Hou[7], Tongchen He[2], Yuanyuan Qiao[2,3], Sanjana Eyunni[2,3,8], Jie Luo[2,3], Chungen Li[1], Yongxing Wang[9], Fengtao Zhou[7], Weixue Huang[1], Xiaomei Ren[1], Zhen Wang[1,*], Arul M. Chinnaiyan[2,3,4,5,10,*], Ke Ding[1,7,11,*]

[1]State Key Laboratory of Chemical Biology, Shanghai Institute of Organic Chemistry, Chinese Academy of Sciences, #345 Lingling Rd., Shanghai 200032, People's Republic of China

[2]Michigan Center for Translational Pathology, University of Michigan, Ann Arbor, MI 48109, USA.

[3]Department of Pathology, University of Michigan, Ann Arbor, MI 48109, USA.

[4]Rogel Cancer Center, University of Michigan, Ann Arbor, MI 48109, USA.

[5]Department of Urology, University of Michigan, Ann Arbor, MI 48109, USA.

[6]Cancer Biology Program, University of Michigan, Ann Arbor, MI 48109, USA.

[7]International Cooperative Laboratory of Traditional Chinese Medicine Modernization and Innovative Drug Discovery of Chinese Ministry of Education (MOE), Guangzhou City Key Laboratory of Precision Chemical Drug Development, College of Pharmacy, Jinan University, 855 Xingye Avenue East, Guangzhou 511400, People's Republic of China

[8]Molecular and Cellular Pathology Program, University of Michigan, Ann Arbor, MI 48109, USA.

[9]Livzon Research Institute, Livzon Pharmaceutical Group Inc., #38 Chuangye North Road, Jinwan District, Zhuhai 519000, China

[10]Howard Hughes Medical Institute, University of Michigan, Ann Arbor, MI 48109, USA.

[11]Hangzhou Institute of Medicine (HlM), Chinese Academy of Sciences, Hangzhou, Zhejiang 310022, China

[#]L. L., A. P. and Y. L. contributed equally to this work.

*Email: wangz@sioc.ac.cn (Z.W.); arul@med.umich.edu (A.M.C.); dingk@sioc.ac.cn; Tel: +86-21-5492 5100 (K.D.)

Table of Contents

1. Degradation profiles of NSD2 by **LLC0424** in various cancer cells....................... S4

2. Mechanism of action and selectivity of **LLC0424**...S5

3. LLC0424 impeded cell proliferation………..………………………………………. S6

4. Distinct degradation and cytotoxicity profile of **LLC0877**…………………………. S7

5. LLC0424 degraded NSD2 in 22RV1 subcutaneous xenograft model.....................S8

6. Sources of antibodies and compounds……………………………………………… S9

7. PK parameters of **LLC0424** in rats………...……………………………………… S10

8. Synthesis of **LLC0424N** (**10lN**) and **LLC0877**…... S11-13

9. ^{1}H and ^{13}C NMR spectra for final compounds **10a-q** S14-30

10. HPLC spectra of final compounds **10a-q**.. S31-47

11. ^{1}H and ^{13}C NMR spectra and HPLC spectra of **LLC0424N** (**10lN**) and **LLC0877**.. S48-51

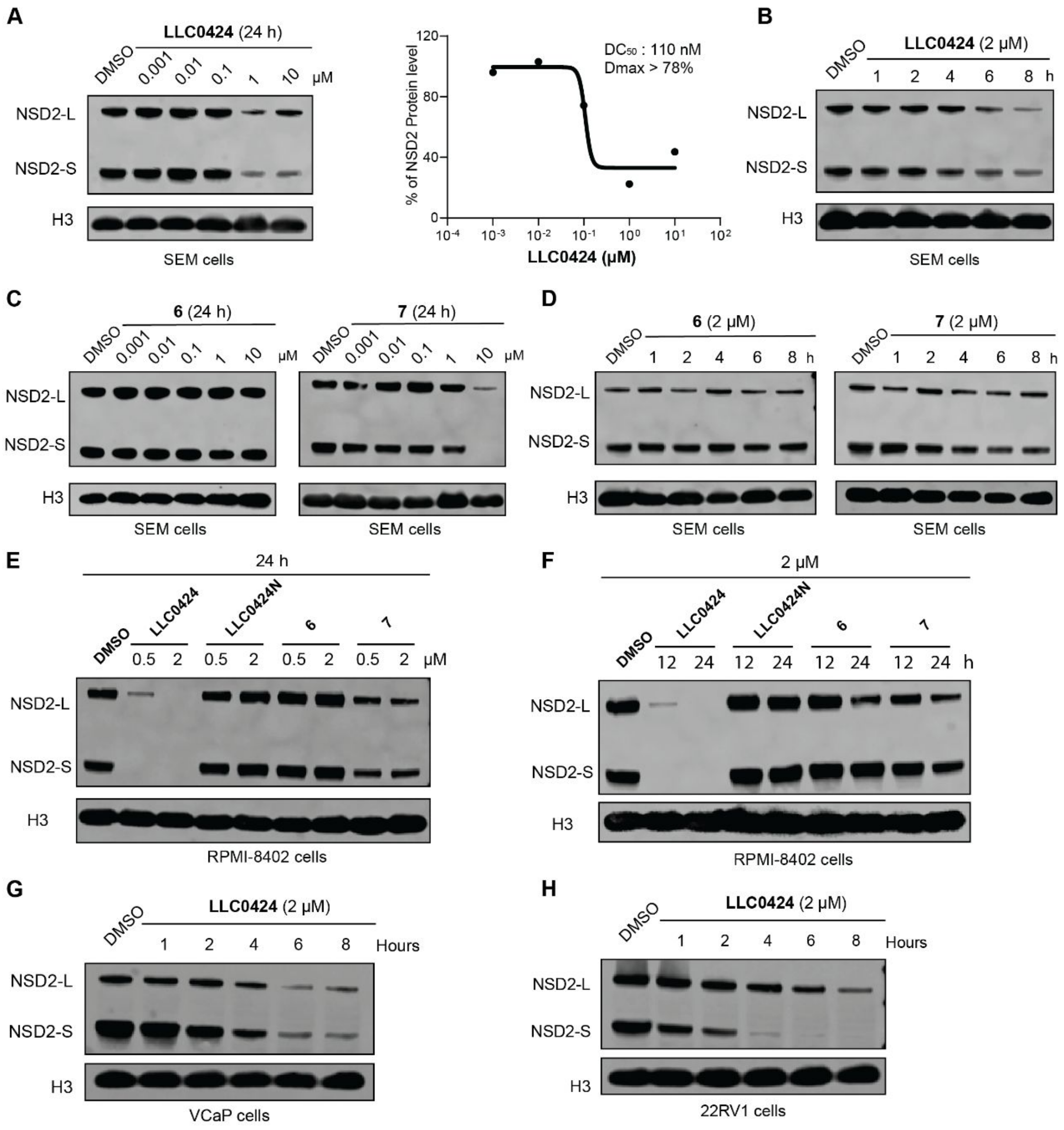

Figure S1. Compound **LLC0424** reduced protein level of both NSD2 isoforms in a concentration- and time-dependent pattern. (A) Immunoblotting of NSD2 (long and short isoforms) and histone3 (H3) in SEM cells treated with increasing concentrations of **LLC0424** for 24 h (left), percent remaining NSD2 protein was plotted for DC_{50} and D_{max} determination (right); (B) Immunoblotting of NSD2 and H3 in SEM cells treated with 2 μM **LLC0424** for various timepoints; (C) Immunoblotting of NSD2 and H3 in SEM cells treated with increasing concentrations of compounds **6** and **7** for 24 h; (D) Immunoblotting of NSD2 and H3 in SEM cells treated with 2 μM compounds **6** and **7** for various timepoints. H3 as the loading control; (E) Immunoblotting of NSD2 and H3 in RPMI-8402 cells treated with 0.5 and 2 μM **LLC0424**, **LLC0424N**, **6** and **7** for 24 h; (F) Immunoblotting of NSD2 and H3 in RPMI-8402 cells treated with 2 μM **LLC0424**, **LLC0424N**, **6** and **7** for 12 and 24 h; Immunoblotting of NSD2 and H3 in VCaP (G) and 22RV1 (H) cells treated with 2 μM **LLC0424** for various timepoints.

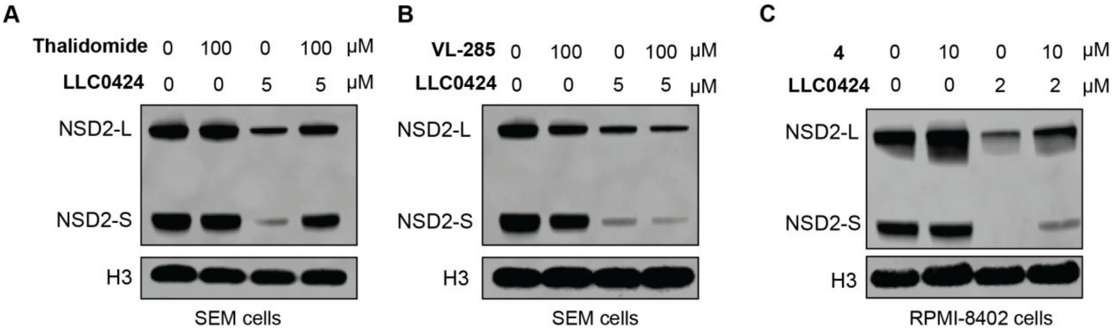

Figure S2. **LLC0424**-mediated NSD2 degradation was CRBN- and proteasome-dependent. Immunoblotting of NSD2 (long and short isoforms) and H3 in SEM cells treated with 2 μM **LLC0424** with or without CRBN ligand thalidomide (A) or VHL ligand VL-285 (B); (C) Immunoblotting of NSD2 and H3 in RPMI-8402 cells treated with 2 μM **LLC0424** with or without the warhead **4**;

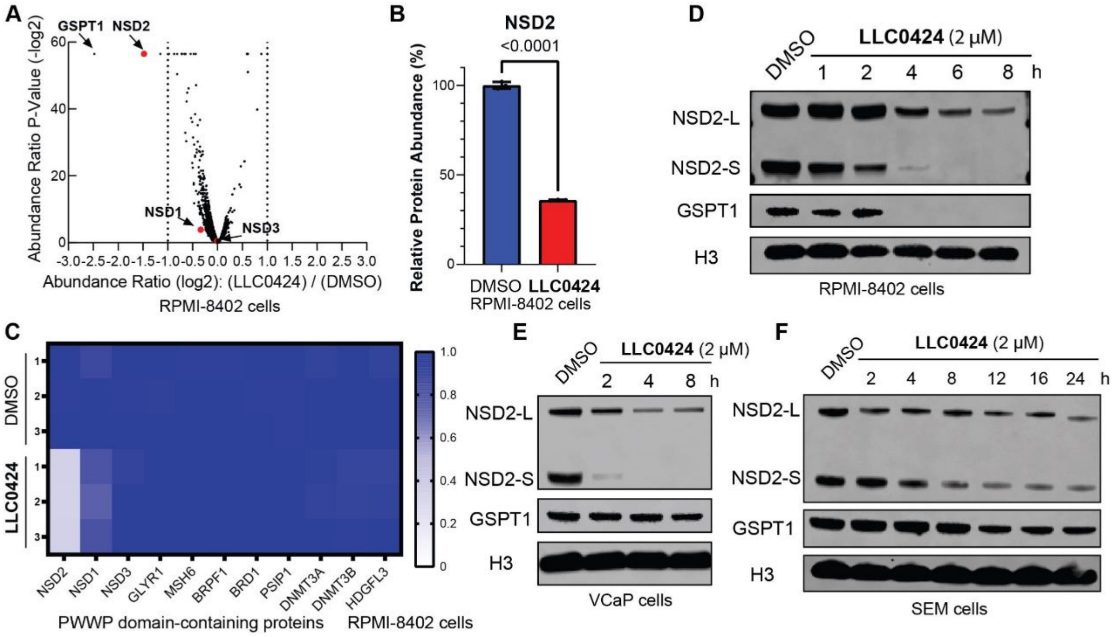

Figure S3. **LLC0424** was a selective NSD2 degrader. (A) Unbiased global proteomics analysis of **LLC0424** in RPMI-8402 cells after 12 h treatment of DMSO or 2 μM **LLC0424**; (B) Mass-spec quantification of NSD2 protein; (C) Heatmap for the level of PWWP-1 domain-containing proteins in RPMI-8402 cells treated with DMSO or 2 μM **LLC0424**; (D) Immunoblotting of NSD2, GSPT1, and H3 in RPMI-8402 cells treated with 2 μM **LLC0424** for various timepoints. H3 was used as a loading control. (E) Immunoblotting of NSD2, GSPT1 and H3 in VCaP cells treated with 2 μM **LLC0424** for various timepoints. H3 was used as a loading control. (F) Immunoblotting of NSD2, GSPT1 and H3 in SEM cells treated with 2 μM **LLC0424** for various timepoints. H3 was used as a loading control.

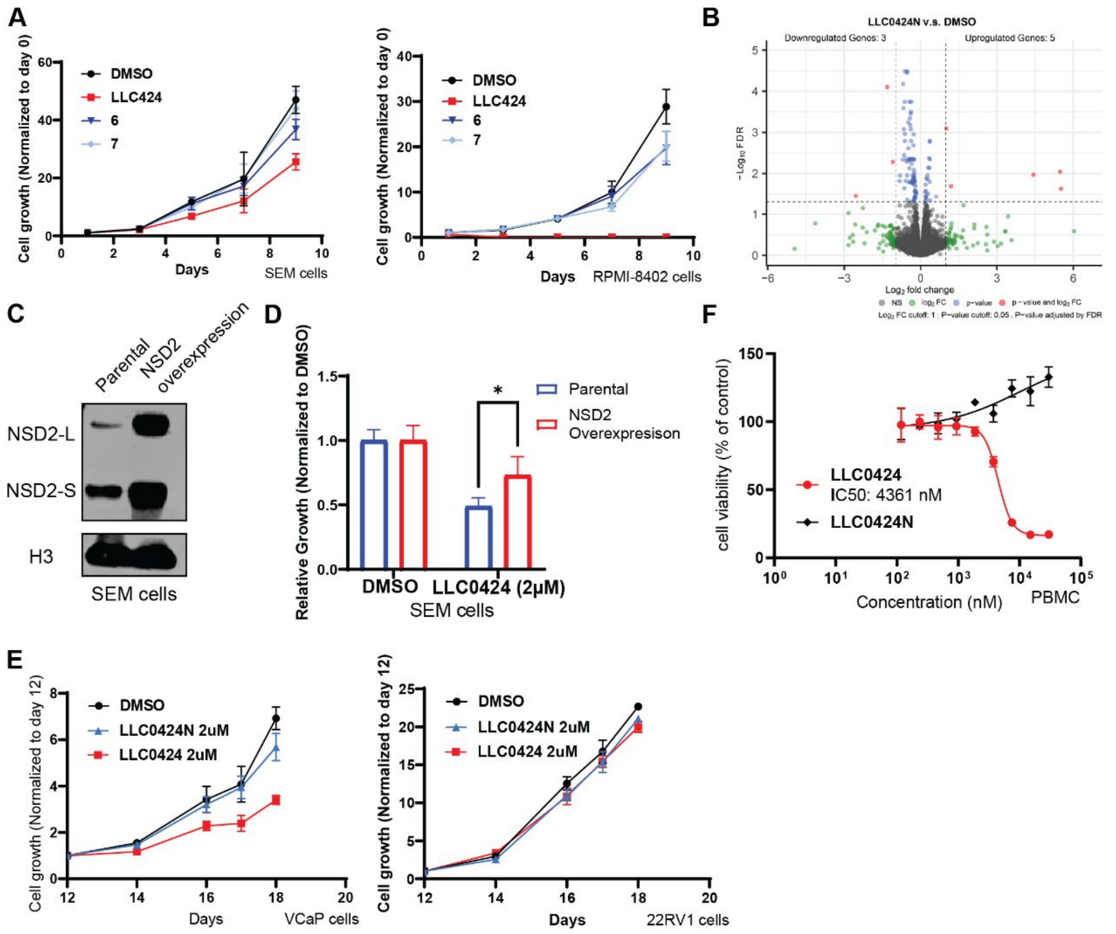

Figure S4. **LLC0424** impeded cell proliferation. (A) SEM or RPMI-8402 cells were treated with 2 μM **LLC0424**, degrader **6**, degrader **7**, or DMSO for 9 days and quantified for cell viability by CellTiter-Glo (CTG) assay at different days. Data are reported as the mean of five independent experiments ± SD; (B) RNA sequencing of SEM cells treated with **LLC0424N** or DMSO; (C) Immunoblotting of NSD2, and H3 in parental and NSD2-overexpression SEM cell lines; (D) parental or NSD2 overexpression SEM cells were treated with DMSO or 2 μM **LLC0424** for 9 days and quantified for cell viability by CTG assay. Data are reported as the mean of four independent experiments ± SD; (E) VCaP cells and 22RV1 cells were treated with DMSO, 2 μM **LLC0424**, or 2 μM **LLC0424N** for 18 days and quantified for cell viability by CTG assay at different days. Data are reported as the mean of four independent experiments ± SD; (F) human peripheral blood mononuclear cells were treated with 2μM **LLC0424** or **LLC0424N** for 7 days and quantified for cell viability by CellTiter-Glo assay at the seventh day. Data are reported as the mean of five independent experiments ± SD.

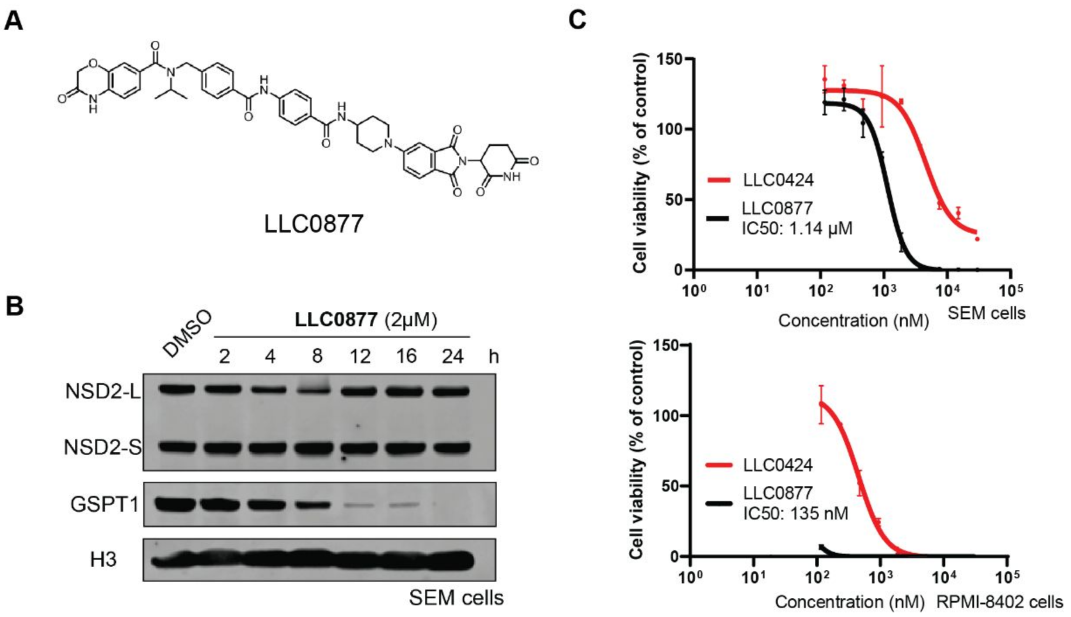

Figure S5. Distinct degradation and cytotoxicity profile of **LLC0877**. (A) Chemical structure of **LLC0877**; (B) Immunoblotting of NSD2, GSPT1 and H3 in SEM cells treated with 2 µM **LLC0424** for various timepoints. H3 was used as a loading control; (C) SEM or RPMI-8402 cells were treated with **LLC0424** or **LLC0877** at varying concentrations for 7 days and quantified for cell viability by CellTiter-Glo assay. Data are reported as the mean of four independent experiments ± SD.

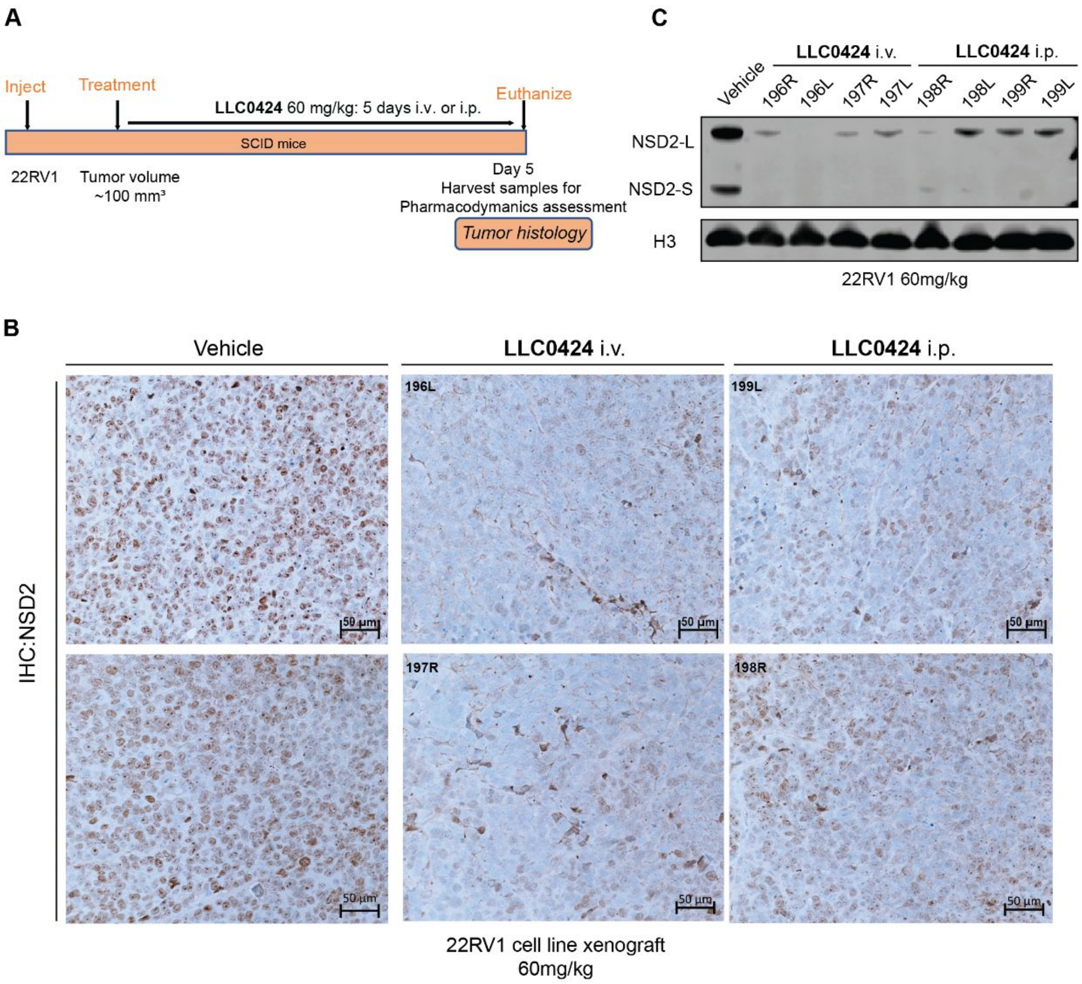

Figure S6. **LLC0424** degraded NSD2 protein *in vivo*. (A) Study design of pharmacodynamics assessment on target engagement of **LLC0424** in the 22RV1 xenograft model; (B) Representative immunohistochemistry images from the 22RV1 xenograft study for NSD2; (C) Immunoblotting of NSD2 and H3 on tissue lysate of 22RV1 xenografts model after **LLC0424** treatment.

Table S1. Sources of all antibodies.

Primary antibodies used				
**Antigen**	**Vendor**	**Catalog number**	**Application**	**Note**
NSD2	Abcam	ab75359	Western Blot	1:1000
NSD1	Antibodies Incorporated	75-280	Western Blot	1:1000
WHSC1L1	Cell Signaling Technology	92056	Western Blot	1:1000
H3	Cell Signaling Technology	3638	Western Blot	1:2500
eRF3	Cell Signaling Technology	14980	Western Blot	1:1000
Ikaros	Cell Signaling Technology	14859	Western Blot	1:1000
Aiolos	Cell Signaling Technology	15103	Western Blot	1:1000
CSNK1A1 + CSNK1A1L	Abcam	ab108296	Western Blot	1:1000
Histone H3 (di methyl K36)	Abcam	ab176921	Western Blot	1:1000
NSD2	Abcam	ab75359	IHC	1:1000
**Secondary antibodies used**				
**Product**	**Vendor**	**Catalog number**	**Application**	**Note**
Goat Anti-Rabbit IgG (H + L)-HRP Conjugate	Bio-Rad	1706515	Western Blot	1:20000
Goat Anti-Mouse IgG (H + L)-HRP Conjugate	Bio-Rad	1706516	Western Blot	1:20000

Table S2. Sources of compounds.

Name	Vendor	Catalog number
Thalidomide	Selleck Chemicals	S1193
VL-285	Selleck Chemicals	S0095
Bortezomib	Selleck Chemicals	S1013
MLN4924	Selleck Chemicals	S7109

Table S4. PK parameters of **LLC0424** in rats[a]

Route/dose	$T_{1/2}$ (h)	T_{max} (h)	C_{max} (ng/mL)	AUC(0-t) (h*ng/mL)	CL (mL/h/kg)	MRT (0-t)	V_{ss} (mL/kg)	F(%)
i.v. (2 mg/kg)	2.10	0.083	7322.67	4323.67	454.73	1.28	762.94	/
Oral (10 mg/kg)	1.52	2.00	162.34	776.60	/	3.18	/	3.67

[a]Determined using LC/MS/MS. $T_{1/2}$: elimination half-life; T_{max}: time for maximum concentration; C_{max}: maximum concentration; AUC: the area under the curve; CL: clearance; MRT: mean residence time; Vss: volume of distribution; F: oral bioavailability

Scheme S1. Synthesis of **LLC0424N (10lN) and LLC0877**

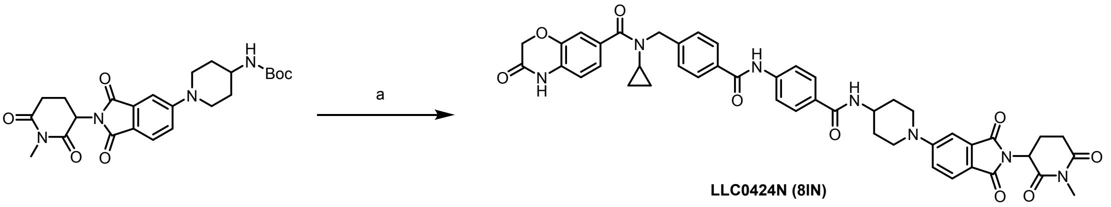

Reagents and conditions: (a) 1) trifluoroacetic acid (TFA), dichloromethane (CH_2Cl_2), room temperature (rt), 2 h; 2) 4-(4-((*N*-cyclopropyl-3-oxo-3,4-dihydro-2*H*-benzo[*b*][1,4]oxazine-7-carboxamido)methyl)benzamido)benzoic acid, *N*-[(dimethylamino)-3-oxo-1*H*-1,2,3-triazolo[4,5-*b*]pyridin-1-yl-methylene]-*N*-methylmethanaminium hexafluorophosphate (HATU), triethylamine (Et_3N), *N, N*-dimethylformamide (DMF), rt, 3 h, 80% (two steps).

N-cyclopropyl-N-(4-((4-((1-(2-(1-methyl-2,6-dioxopiperidin-3-yl)-1,3-dioxoisoindolin-5-yl)piperidin-4-yl)carbamoyl)phenyl)carbamoyl)benzyl)-3-oxo-3,4-dihydro-2H-benzo[b][1,4]oxazine-7-carboxamide ***LLC0424N (10lN)****.* *tert*-butyl (1-(2-(1-methyl-2,6-dioxopiperidin-3-yl)-1,3-dioxoisoindolin-5-yl)piperidin-4-yl)carbamate (250.0 mg, 0.531 mmol) was dissolved in CH_2Cl_2 (2.0 mL). To above solution was added TFA (2.0 mL) by syringe. The mixture was stirred at room temperature for 2 h. After the reaction was complete, the solvent was removed by vacuum. The crude product was dissolved in a small amount of DMF (2.0 mL) and were added 4-(4-((N-cyclopropyl-3-oxo-3,4-dihydro-2H-benzo[*b*][1,4]oxazine-7-carboxamido)methyl)benzamido)benzoic acid (284 mg, 0.584 mmol), triethylamine (161 mg, 0.22 mL, 1.594 mmol), and HATU (242 mg, 0.638 mmol). The mixture was stirred at room temperature for 3 h. The resulting mixture was purified by column chromatography to afford the title compound as a yellow solid (356 mg, 80% yield). ^1H NMR (600 MHz, DMSO-d_6) δ 10.88 (s, 1H), 10.42 (s, 1H), 8.19 (d, J = 7.8 Hz, 1H), 7.96 (d, J = 8.2 Hz, 2H), 7.86 (s, 4H), 7.68 (d, J = 8.5 Hz, 1H), 7.46 (d, J = 7.7 Hz, 2H), 7.38 (d, J = 2.1 Hz, 1H), 7.30 (dd, J = 8.7, 2.3 Hz, 1H), 7.19 (d, J = 7.9 Hz, 1H), 7.15 (s, 1H), 6.93 (d, J = 8.1 Hz, 1H), 5.15 (dd, J = 13.1, 5.4 Hz, 1H), 4.72 (s, 2H), 4.62 (s, 2H), 4.18 – 4.04 (m, 3H), 3.18 – 3.12 (m, 2H), 3.02 (s, 3H), 2.99 – 2.92 (m, 1H), 2.85 – 2.73 (m, 2H), 2.62 – 2.52 (m, 1H), 2.08 – 2.01 (m, 1H), 1.94 – 1.86 (m, 2H), 1.68 – 1.57 (m, 2H), 0.60 – 0.51 (m, 2H), 0.50 – 0.42 (m, 2H). ^{13}C NMR (151 MHz, DMSO-d_6) δ 171.80, 169.88, 167.63, 166.96, 165.56, 165.04, 164.85, 154.77, 142.48, 142.35,

141.78, 134.09, 133.40, 131.78, 129.38, 128.38, 128.05, 127.99, 127.20, 125.08, 121.87, 119.27, 117.87, 117.59, 115.36, 115.15, 107.99, 66.74, 64.92, 49.35, 46.55, 46.49, 45.75, 31.15, 30.47, 26.61, 21.39, 15.17, 8.64. HRMS (m/z): $[M + H]^+$ calcd for $C_{46}H_{44}N_7O_9^+$ 838.3195, found 838.3191. HPLC purity: 97.93%.

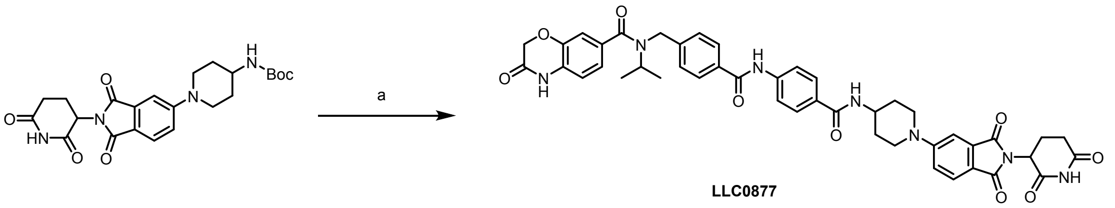

Reagents and conditions: (a) 1) TFA, CH_2Cl_2, rt, 2 h; 2) 4-(4-((N-isopropyl-3-oxo-3,4-dihydro-2H-benzo[b][1,4]oxazine-7-carboxamido)methyl)benzamido)benzoic acid, HATU, Et_3N, DMF, rt, 3 h, 82% (two steps).

N-(4-((4-((1-(2-(2,6-dioxopiperidin-3-yl)-1,3-dioxoisoindolin-5-yl)piperidin-4-yl)carbamoyl)phenyl)carbamoyl)benzyl)-N-isopropyl-3-oxo-3,4-dihydro-2H-benzo[b][1,4]oxazine-7-carboxamide ***LLC0877.*** *tert-butyl (1-(2-(2,6-dioxopiperidin-3-yl)-1,3-dioxoisoindolin-5-yl)piperidin-4-yl)carbamate* (50.0 mg, 0.110 mmol) was dissolved in CH_2Cl_2 (1.0 mL). To above solution was added TFA (1.0 mL) by syringe. The mixture was stirred at room temperature for 2 h. After the reaction was complete, the solvent was removed by vacuum. The crude product was dissolved in a small amount of DMF (1.0 mL) and were added 4-(4-((N-isopropyl-3-oxo-3,4-dihydro-2H-benzo[b][1,4]oxazine-7-carboxamido)methyl)benzamido)benzoic acid (53.4 mg, 0.110 mmol), triethylamine (33.3 mg, 46 μL, 0.330 mmol), and HATU (50.0 mg, 0.131 mmol). The mixture was stirred at room temperature for 1 h. The resulting mixture was purified by column chromatography to afford the title compound as a yellow solid (74.5 mg, 82% yield). ^1H NMR (600 MHz, DMSO-d_6) δ 11.08 (s, 1H), 10.86 (s, 1H), 10.40 (s, 1H), 8.19 (d, J = 7.8 Hz, 1H), 7.91 (d, J = 8.3 Hz, 2H), 7.88 – 7.82 (m, 4H), 7.68 (d,

J = 8.5 Hz, 1H), 7.51 – 7.42 (m, 2H), 7.38 (d, J = 1.9 Hz, 1H), 7.29 (dd, J = 8.7, 2.2 Hz, 1H), 7.10 – 7.00 (m, 2H), 7.00 – 6.91 (m, 1H), 5.08 (dd, J = 12.8, 5.4 Hz, 1H), 4.73 – 4.52 (m, 4H), 4.22 – 4.00 (m, 4H), 3.20 – 3.09 (m, 2H), 2.94 – 2.83 (m, 1H), 2.63 – 2.53 (m, 2H), 2.07 – 1.98 (m, 1H), 1.94 – 1.83 (m, 2H), 1.67 – 1.55 (m, 2H), 1.17 – 1.04 (m, 6H). ^{13}C NMR (151 MHz, DMSO-d_6) δ 172.82, 170.17, 170.13, 167.63, 166.98, 165.04, 164.74, 154.77, 141.82, 134.10, 129.33, 128.13, 127.99, 127.76, 126.56, 125.07, 120.50, 119.25, 117.85, 117.62, 114.22, 107.99, 66.73, 48.77, 46.57, 46.49, 30.99, 30.47, 22.19, 20.78. HRMS (m/z): [M + Na]$^+$ calcd for $C_{45}H_{43}N_7O_9Na^+$ 848.3014, found 848.3011. HPLC purity: 97.89%.

^{1}H and ^{13}C NMR Spectra for final compounds

^{1}H NMR and ^{13}C NMR spectra for **10a**

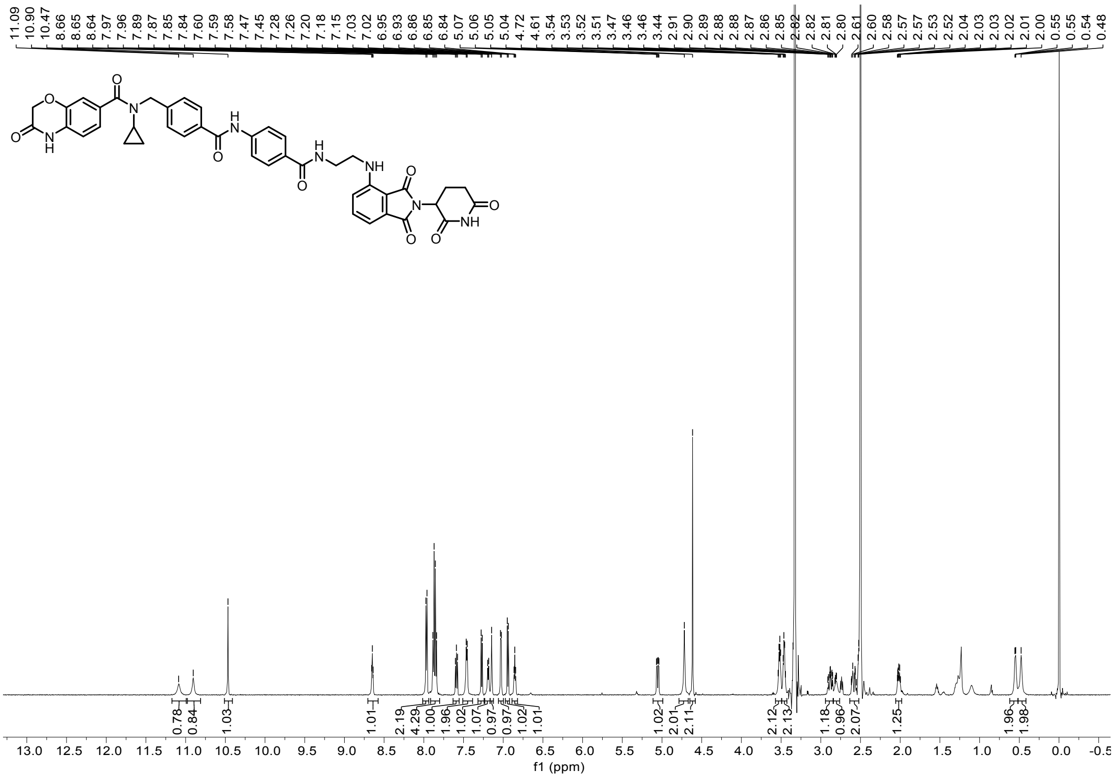

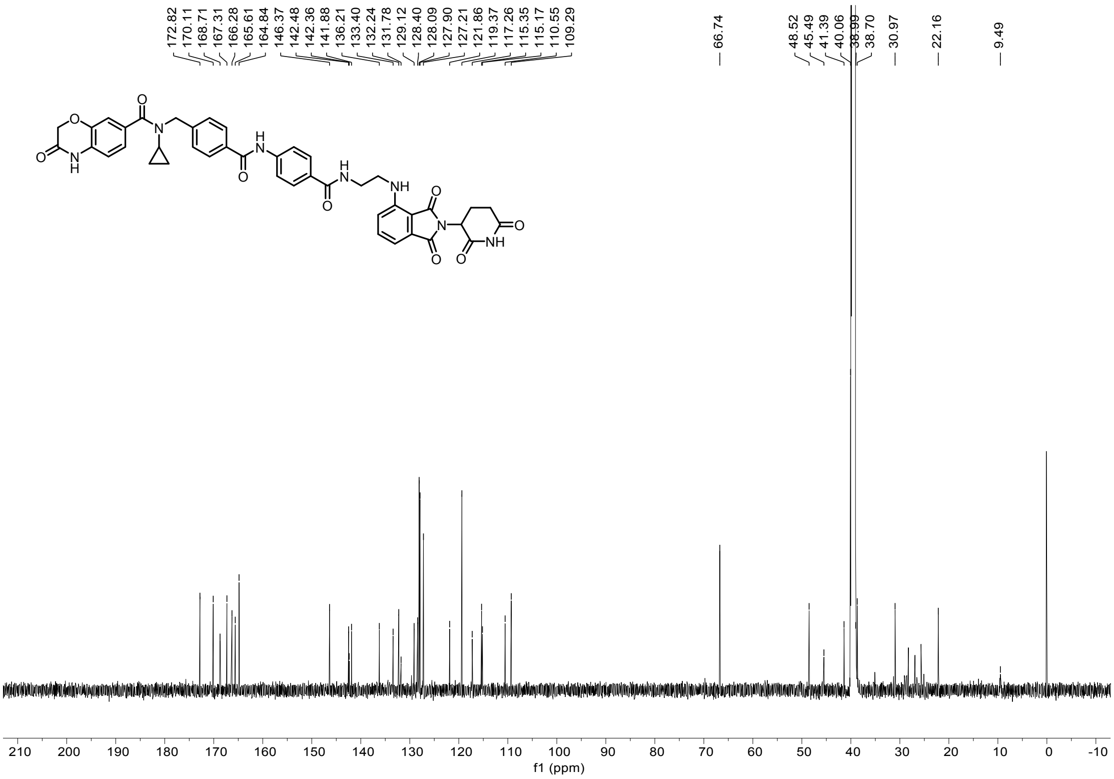

^{1}H NMR and ^{13}C NMR spectra for **10b**

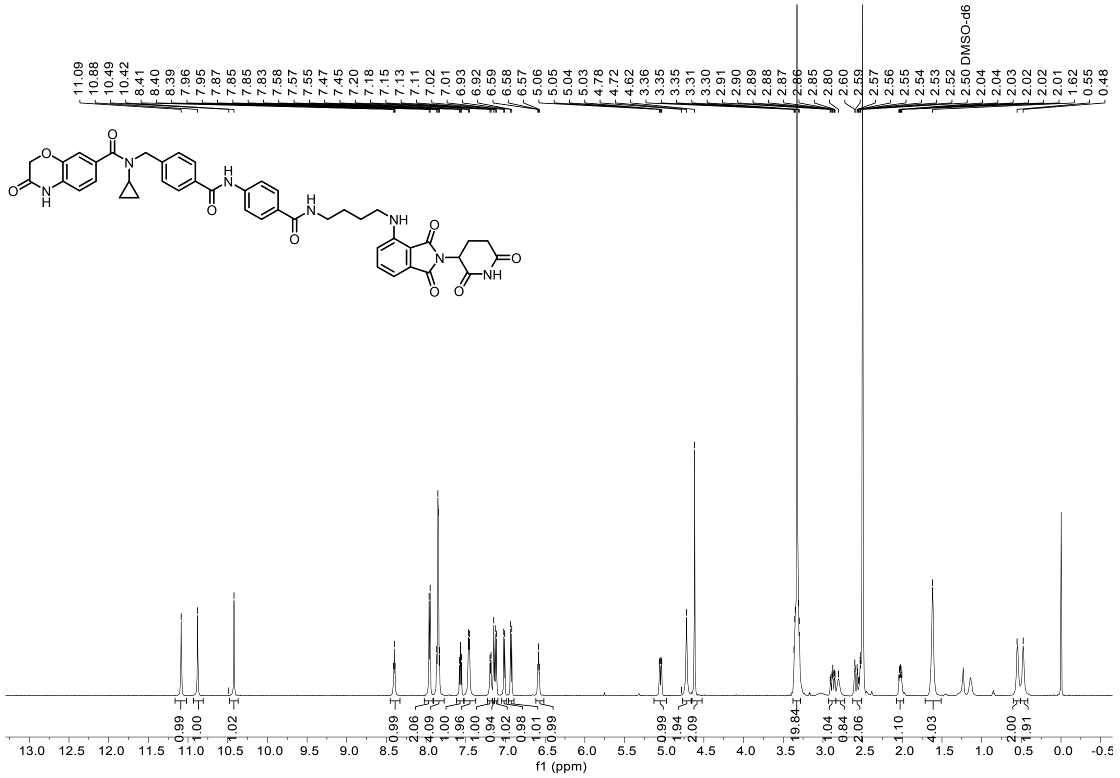

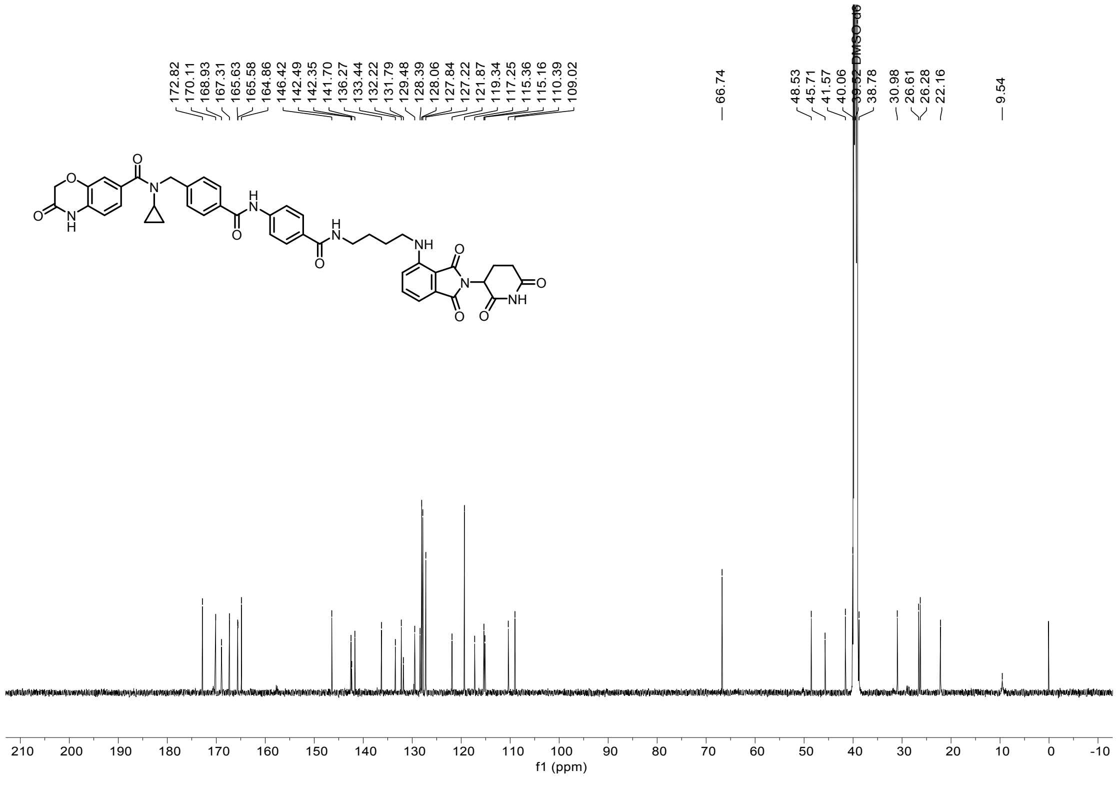

^{1}H NMR and ^{13}C NMR spectra for **10c**

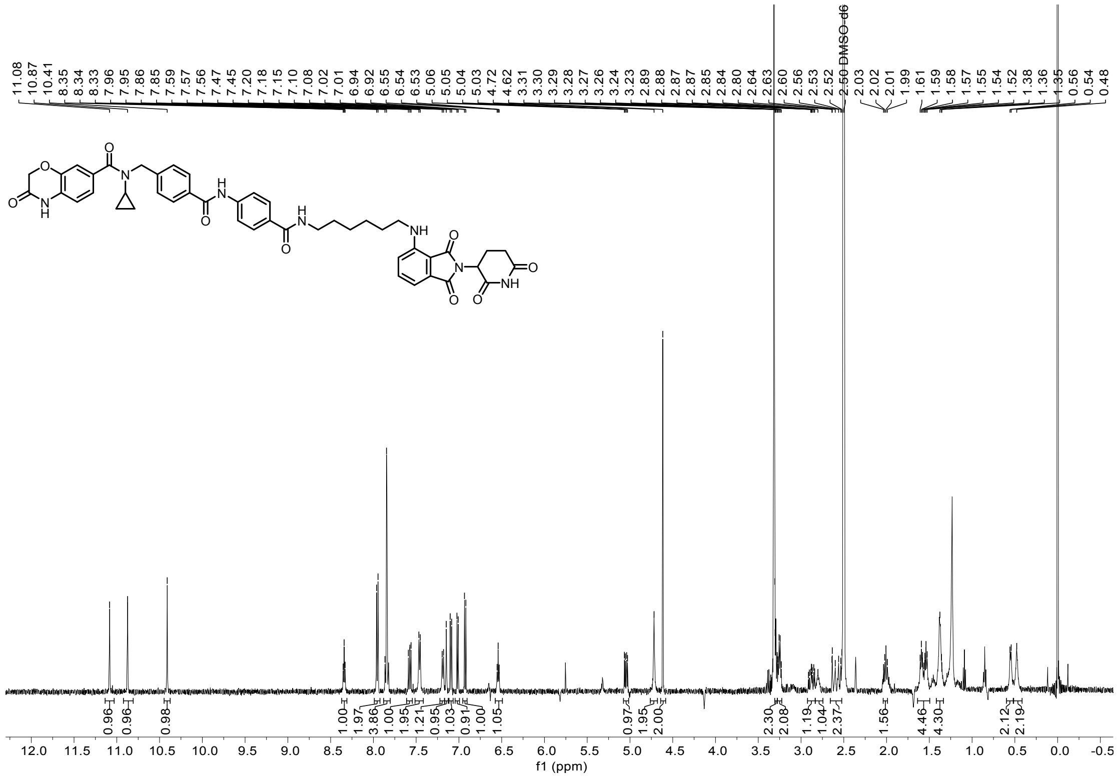

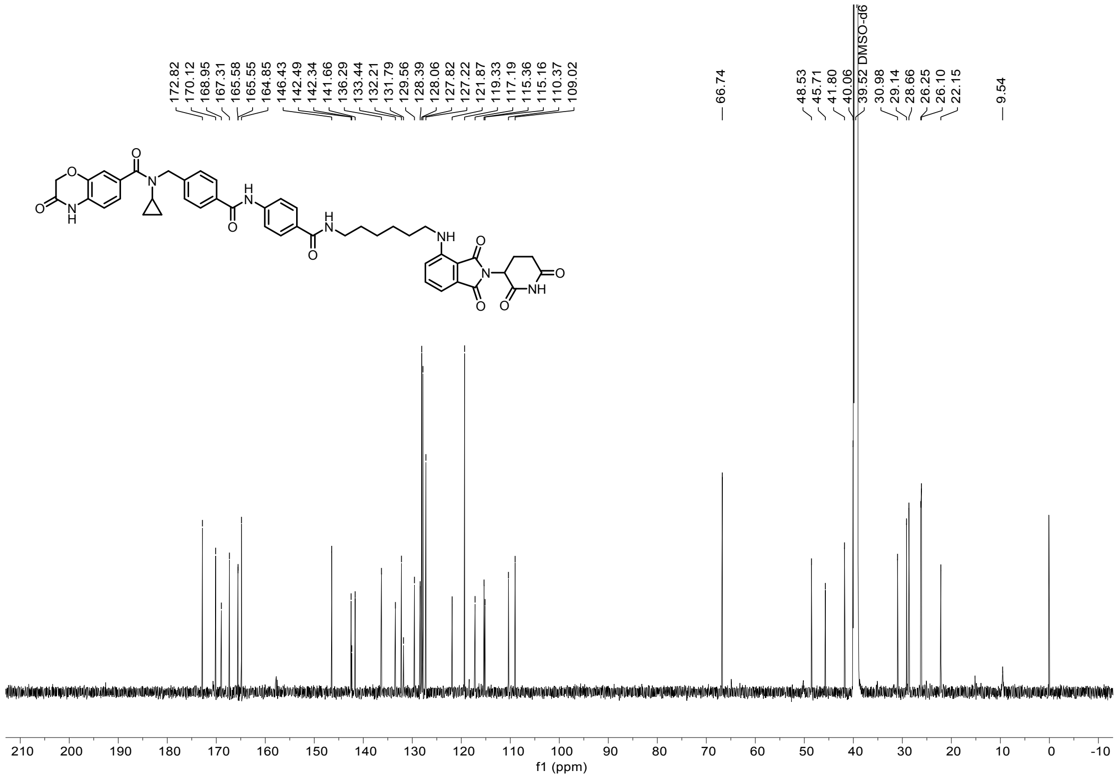

^{1}H NMR and ^{13}C NMR spectra for **10d**

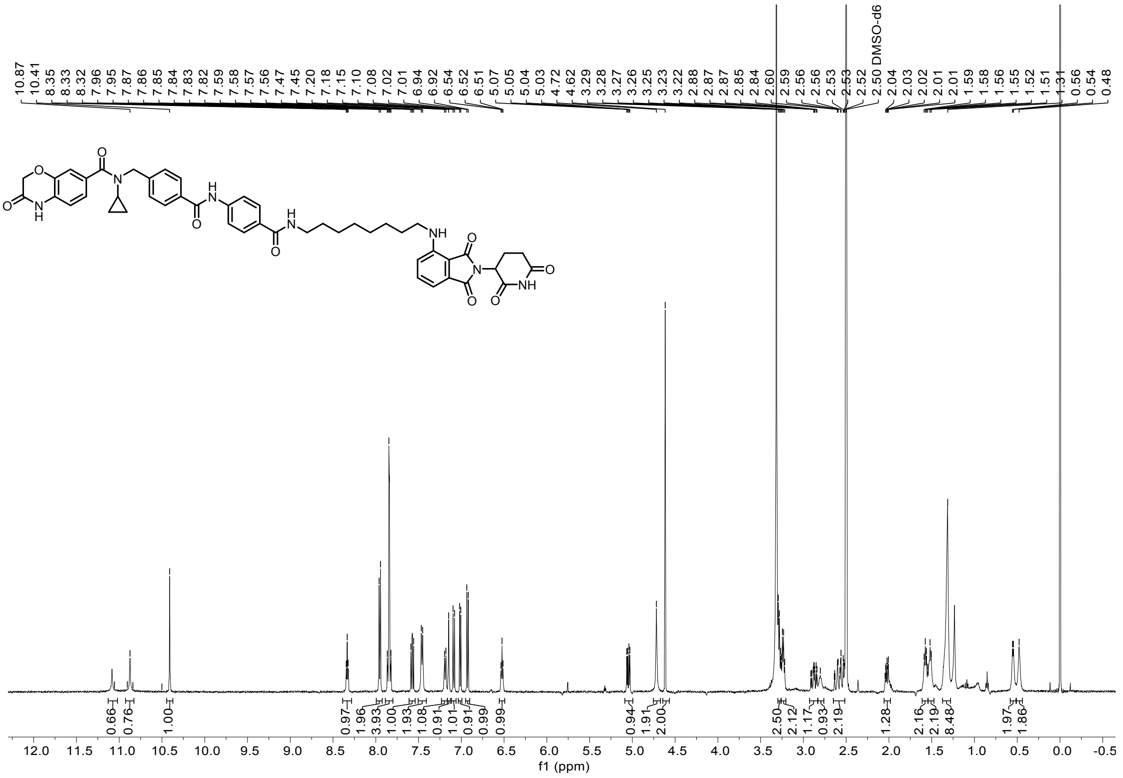

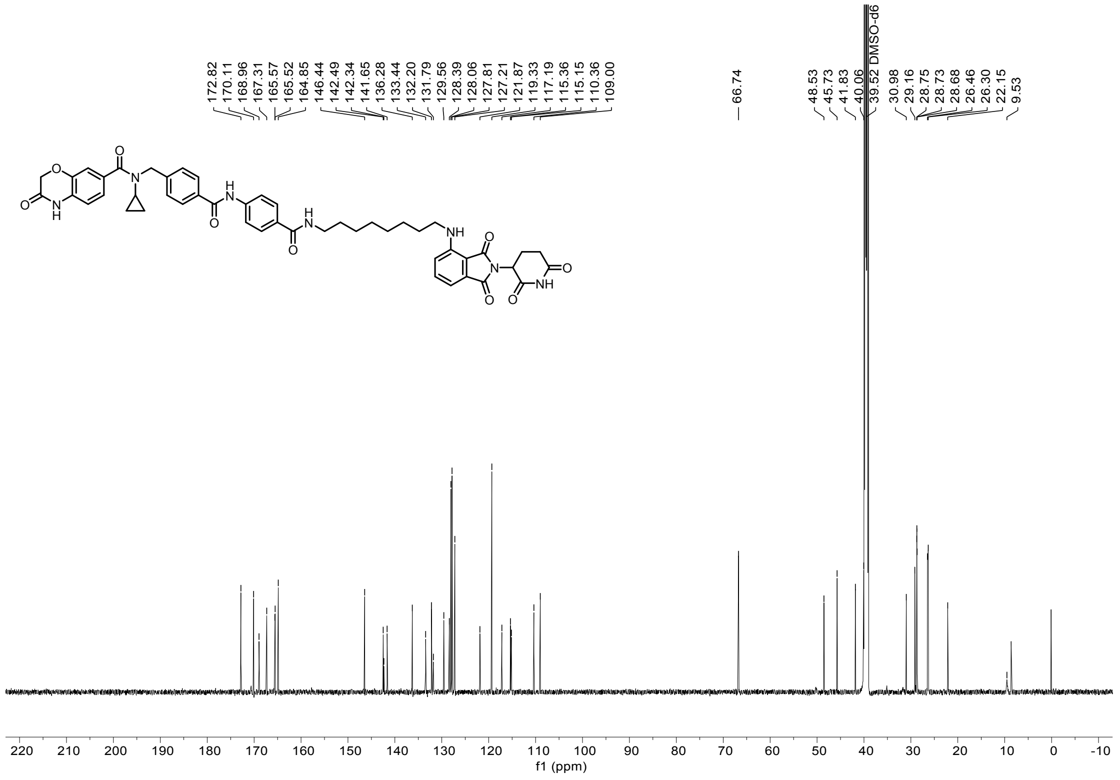

^{1}H NMR and ^{13}C NMR spectra for **10e**

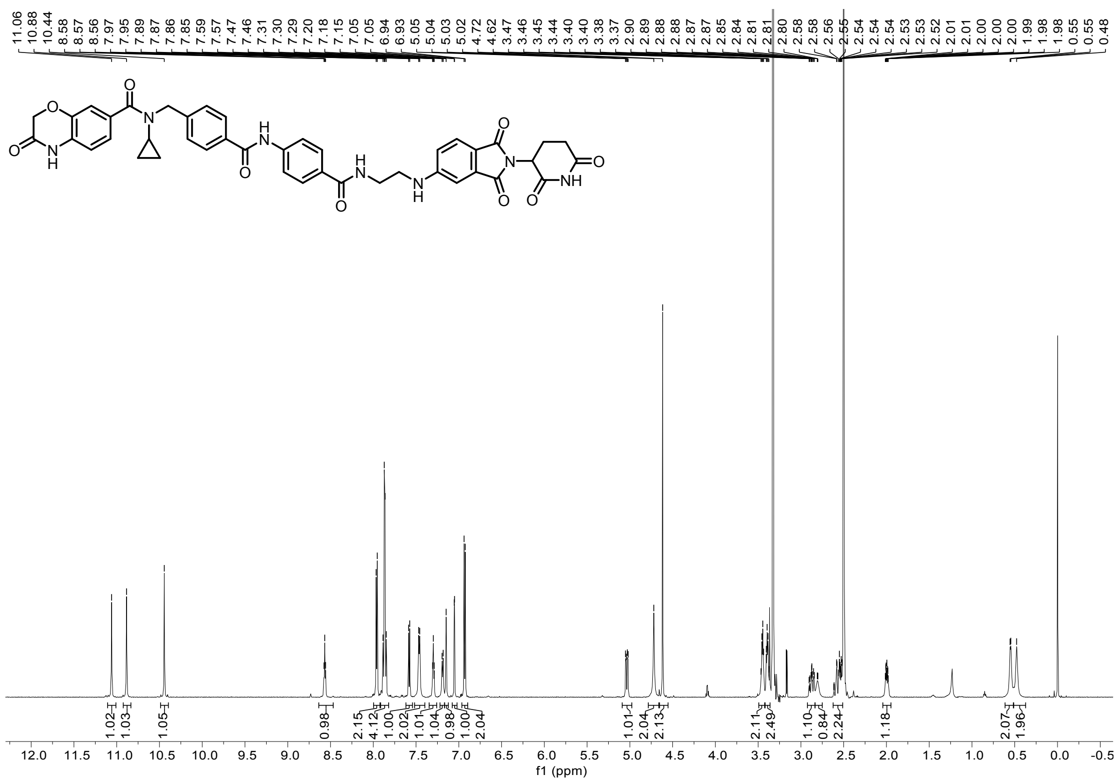

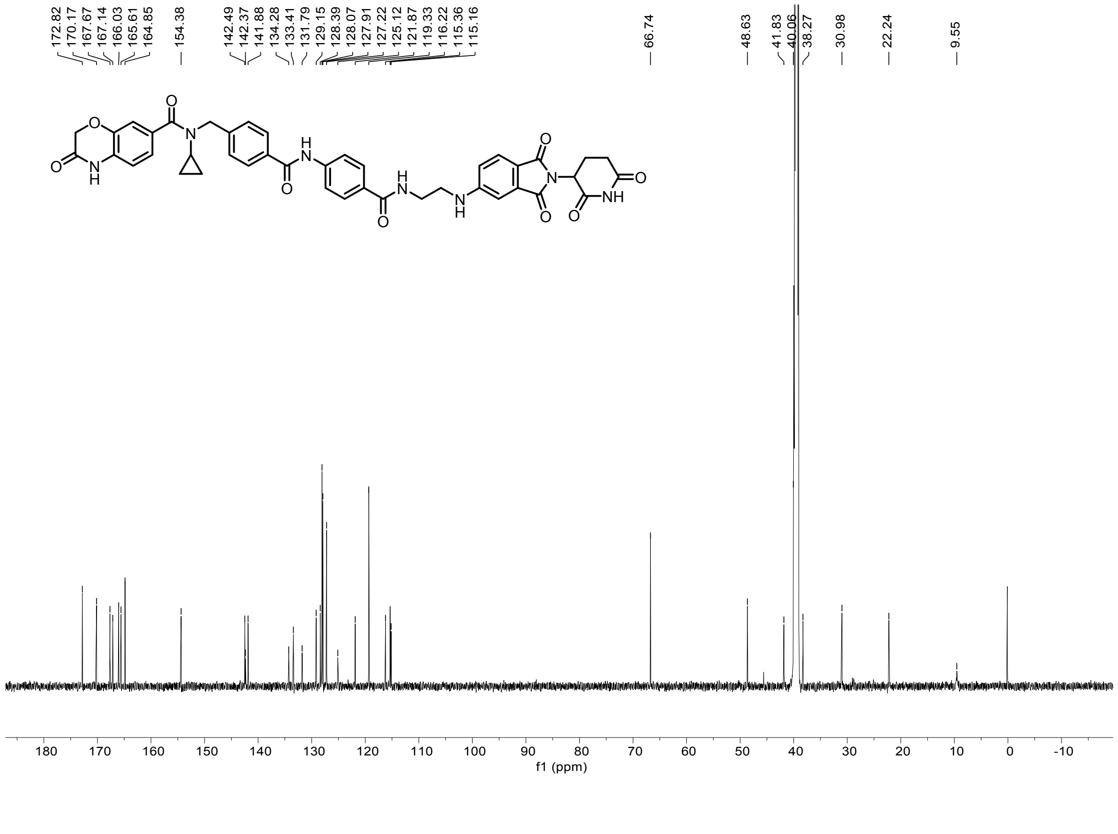

^{1}H NMR and ^{13}C NMR spectra for **10f**

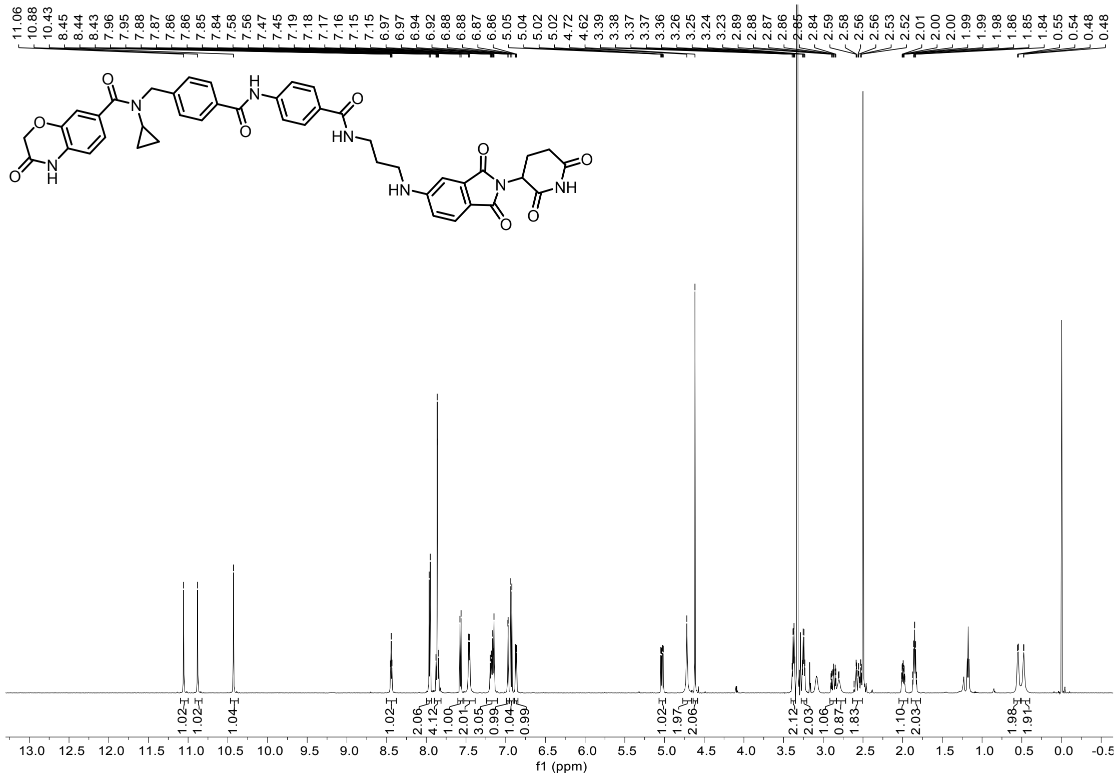

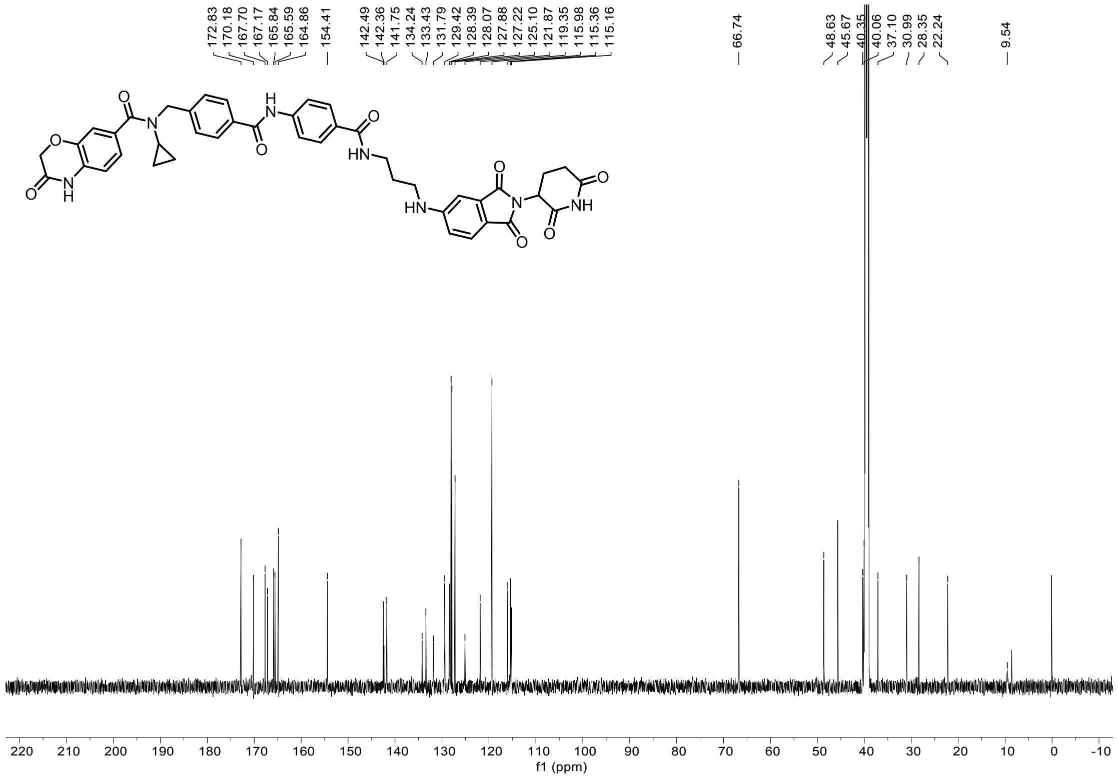

^{1}H NMR and ^{13}C NMR spectra for **10g**

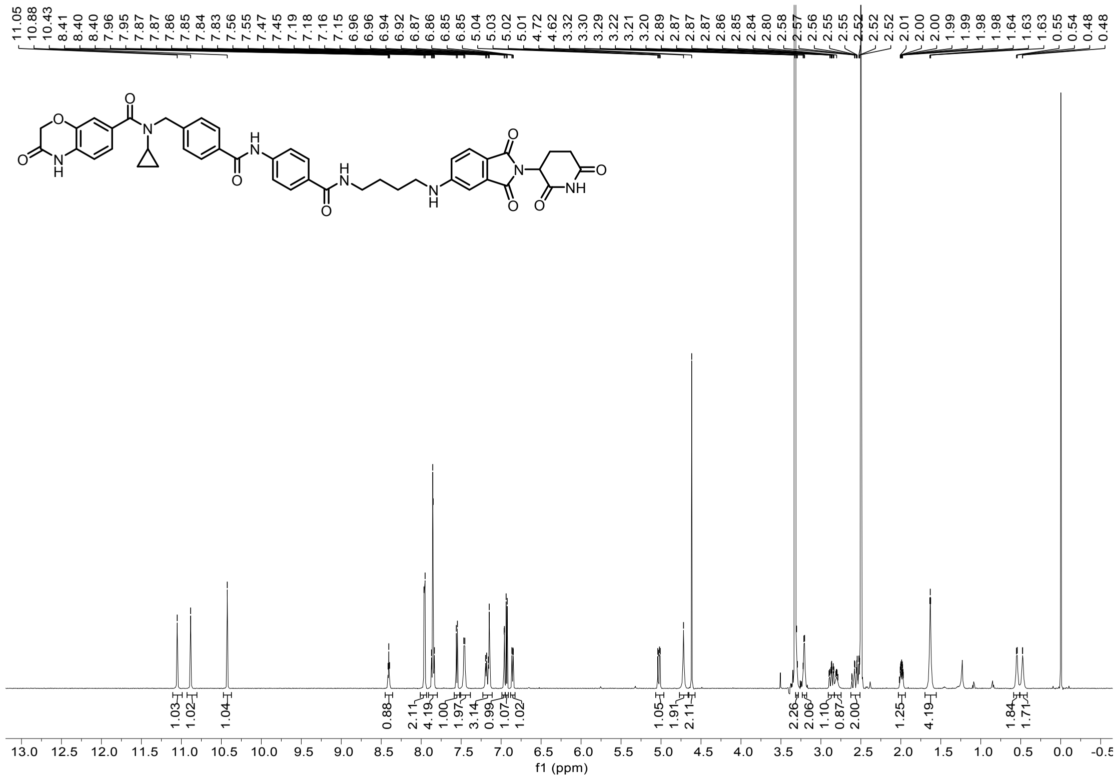

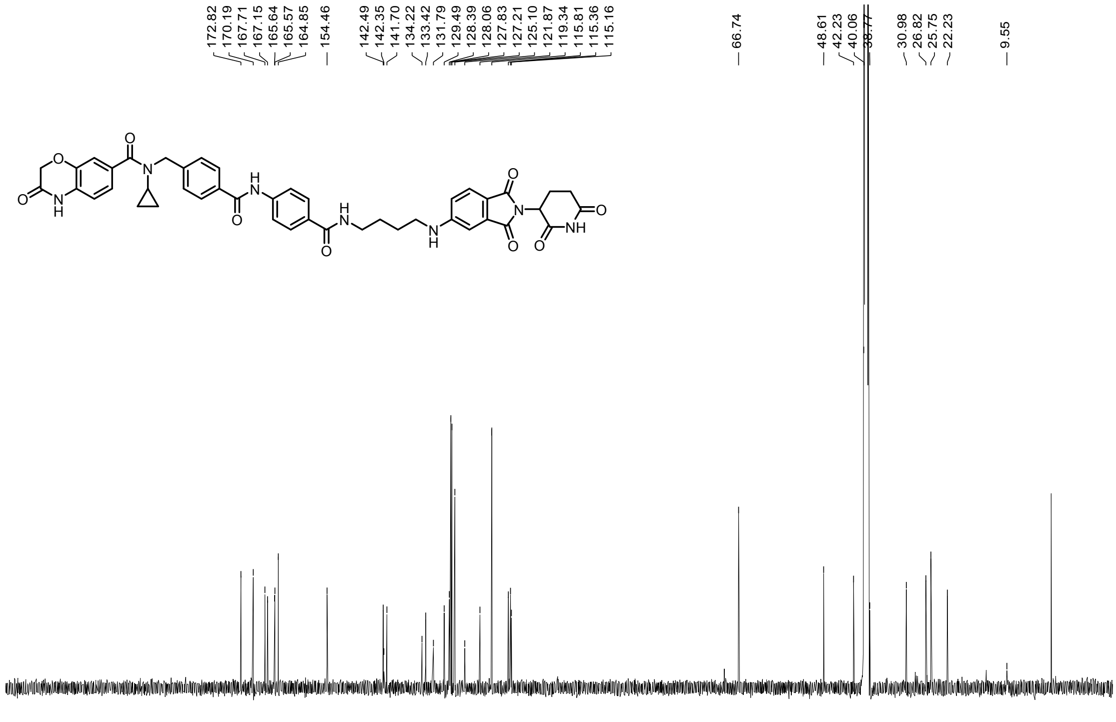

^{1}H NMR and ^{13}C NMR spectra for **10h**

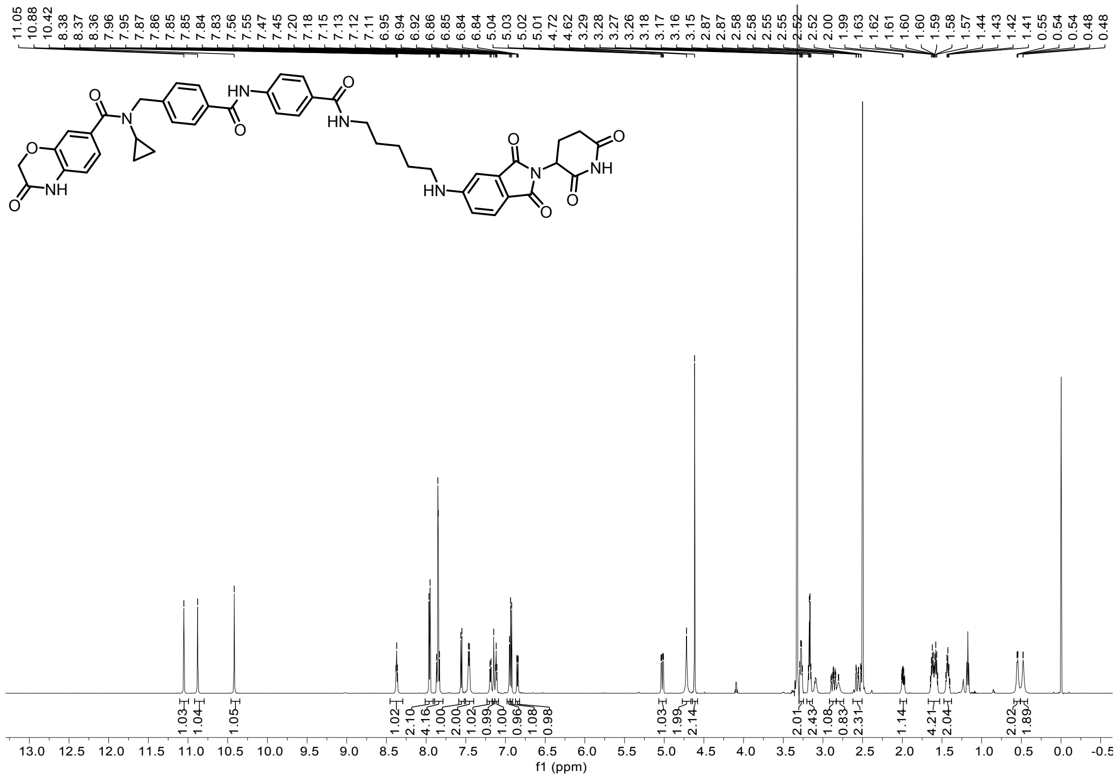

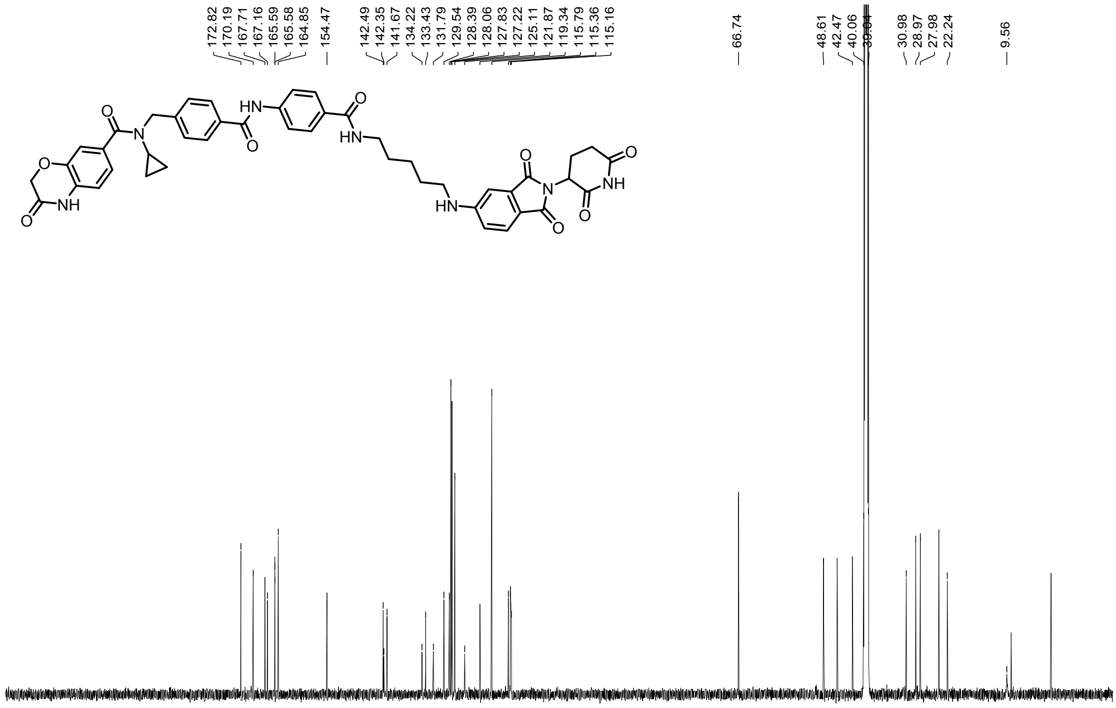

^{1}H NMR and ^{13}C NMR spectra for **10i**

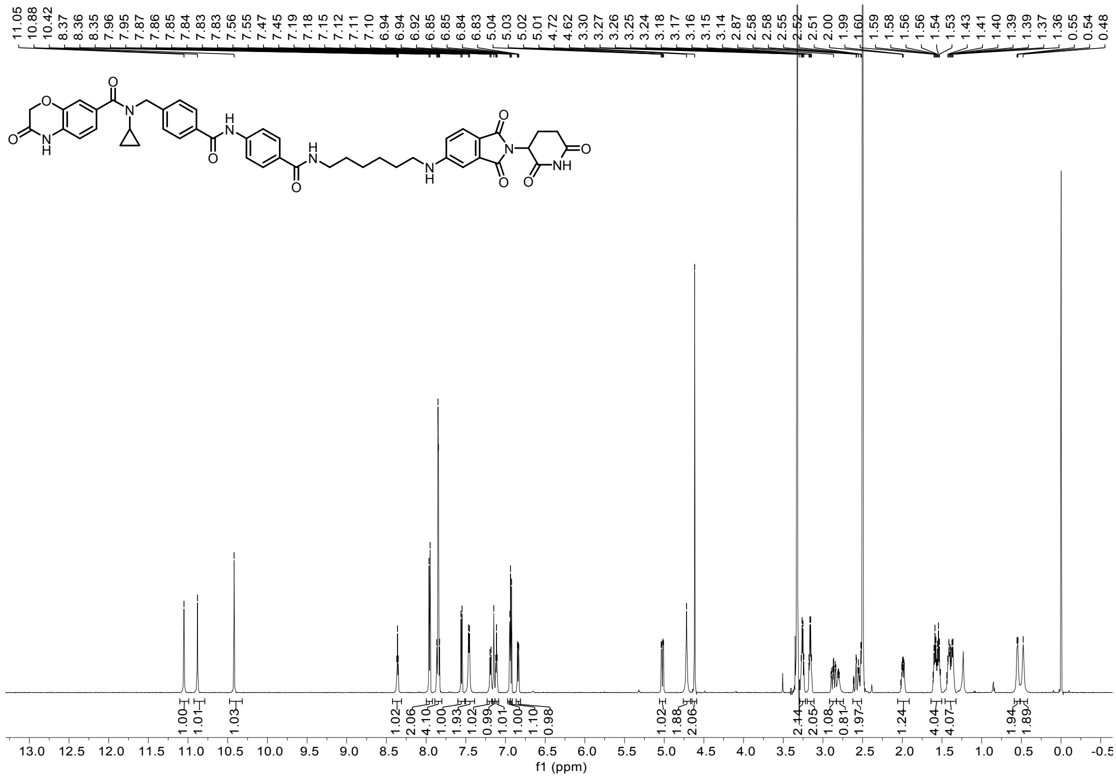

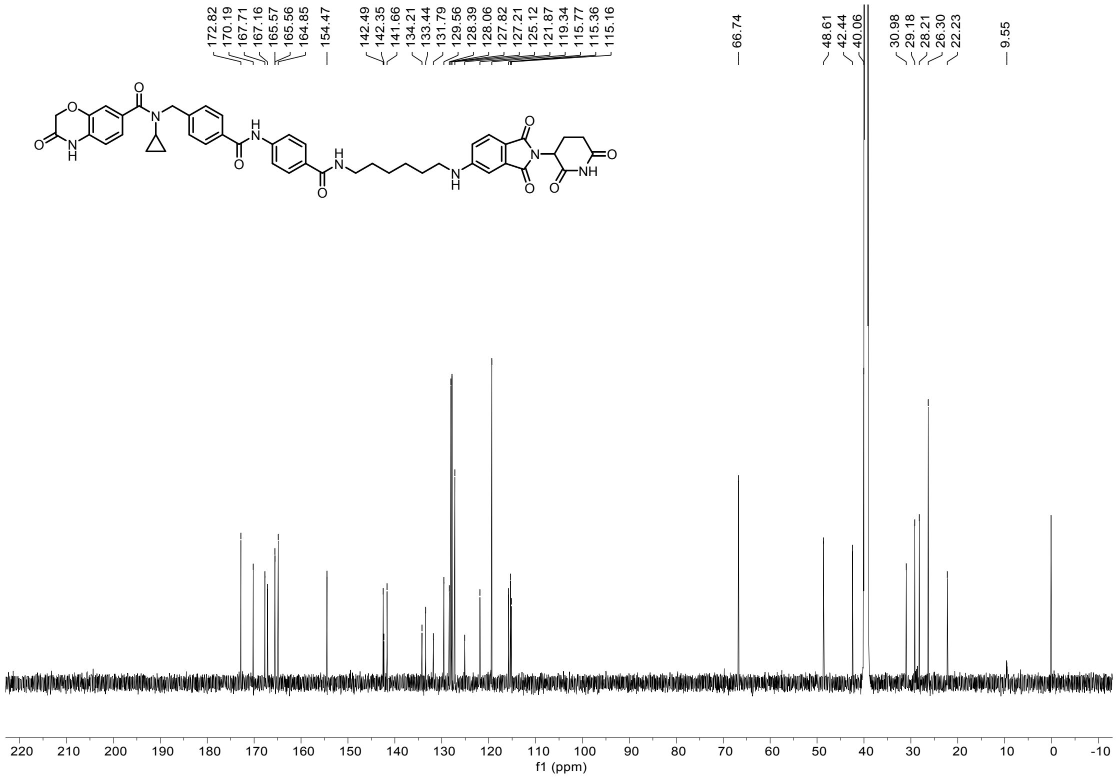

^{1}H NMR and ^{13}C NMR spectra for **10j**

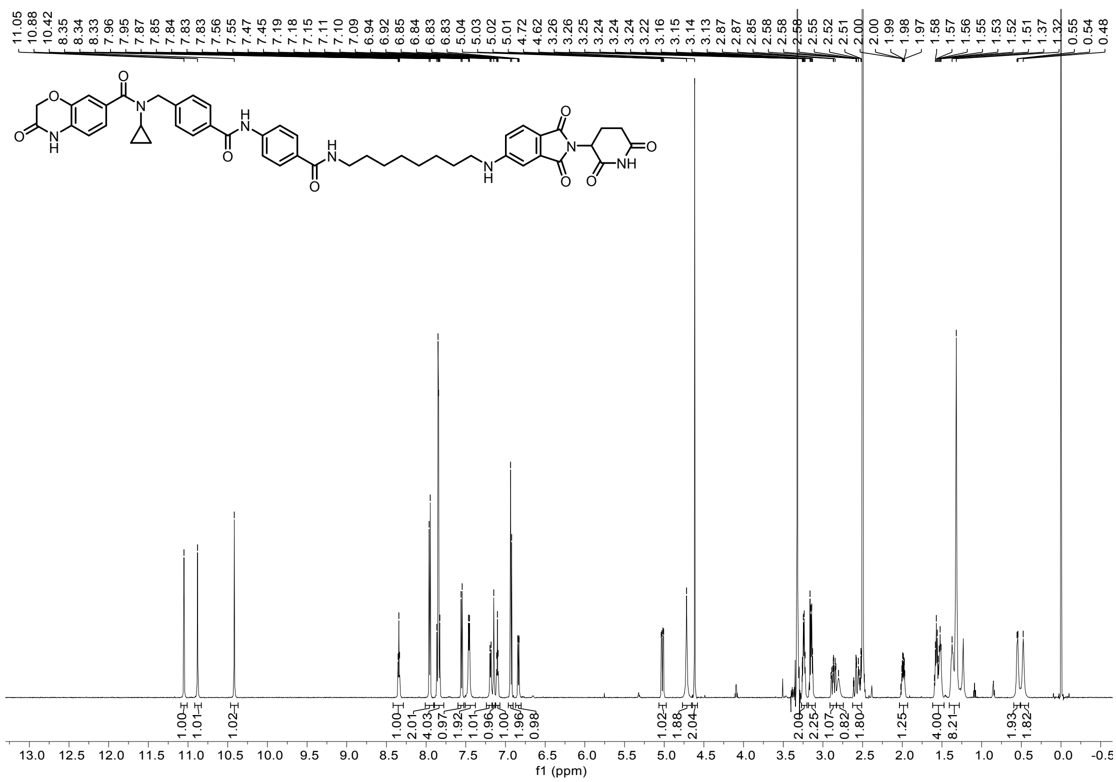

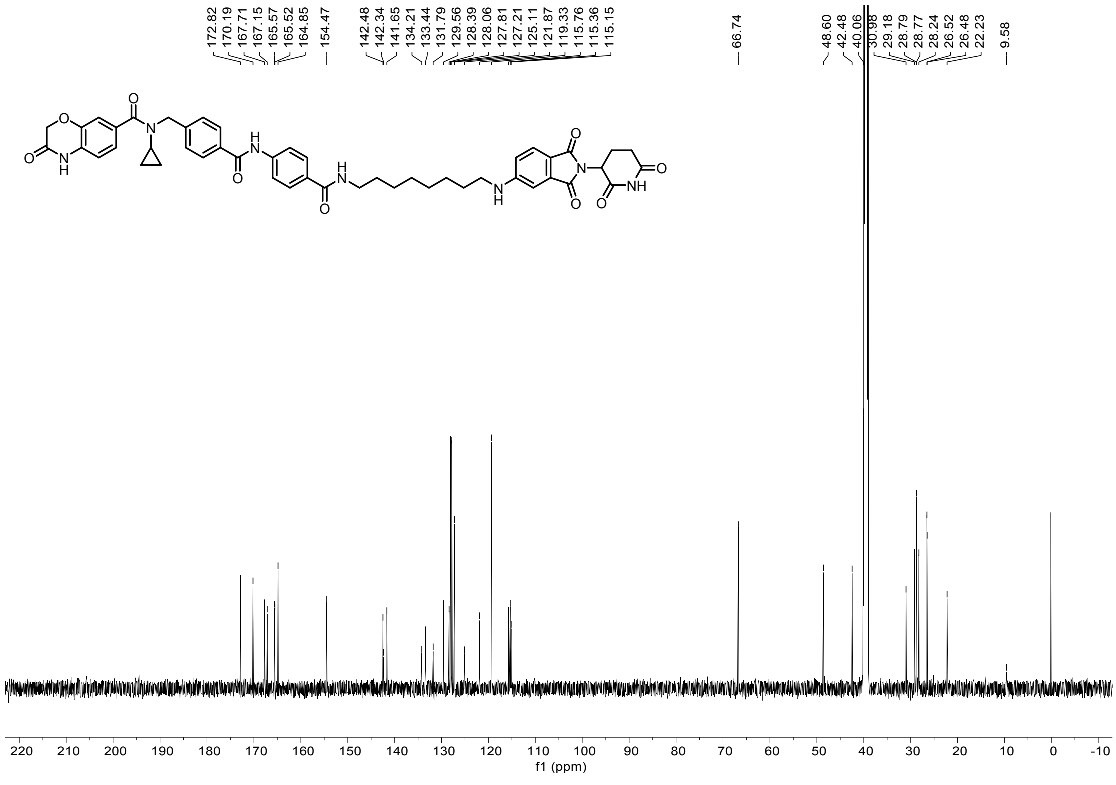

^{1}H NMR and ^{13}C NMR spectra for **10k**

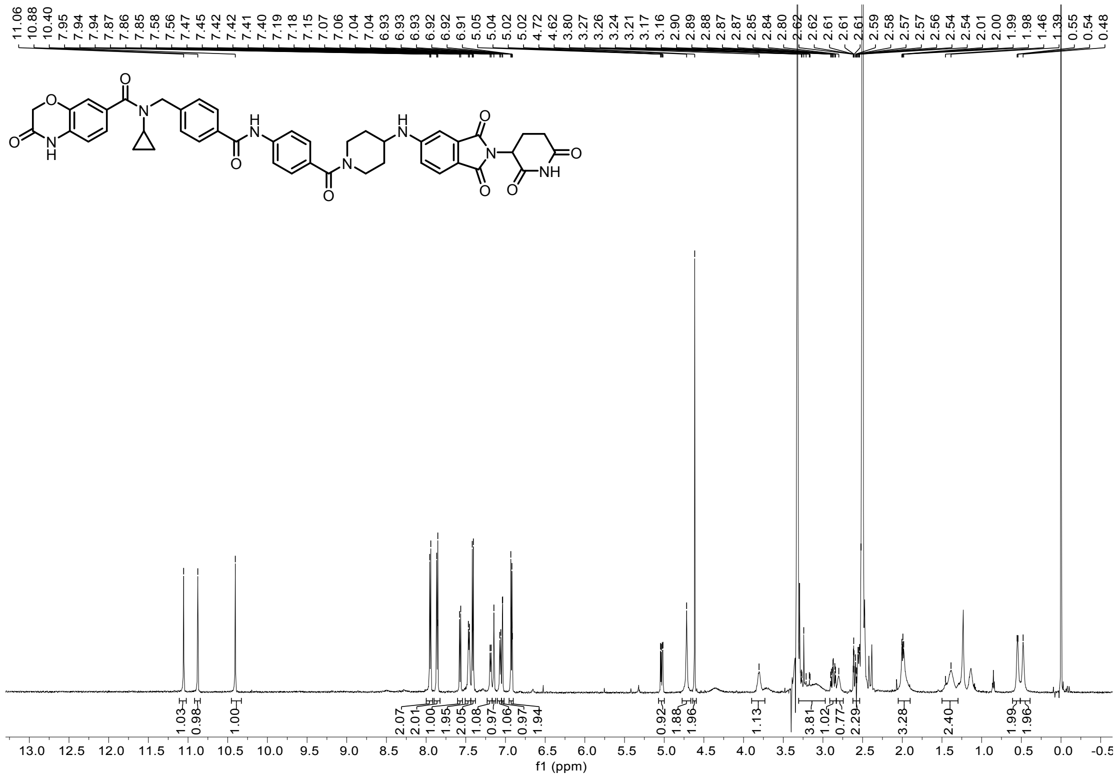

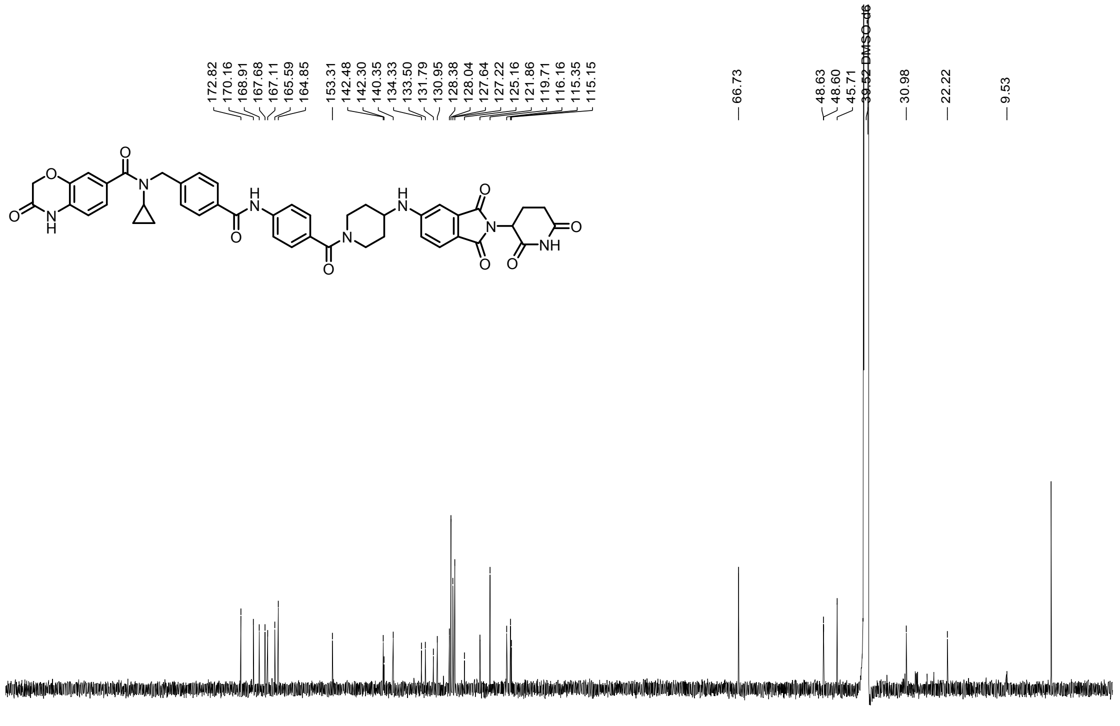

^{1}H NMR and ^{13}C NMR spectra for **10l**

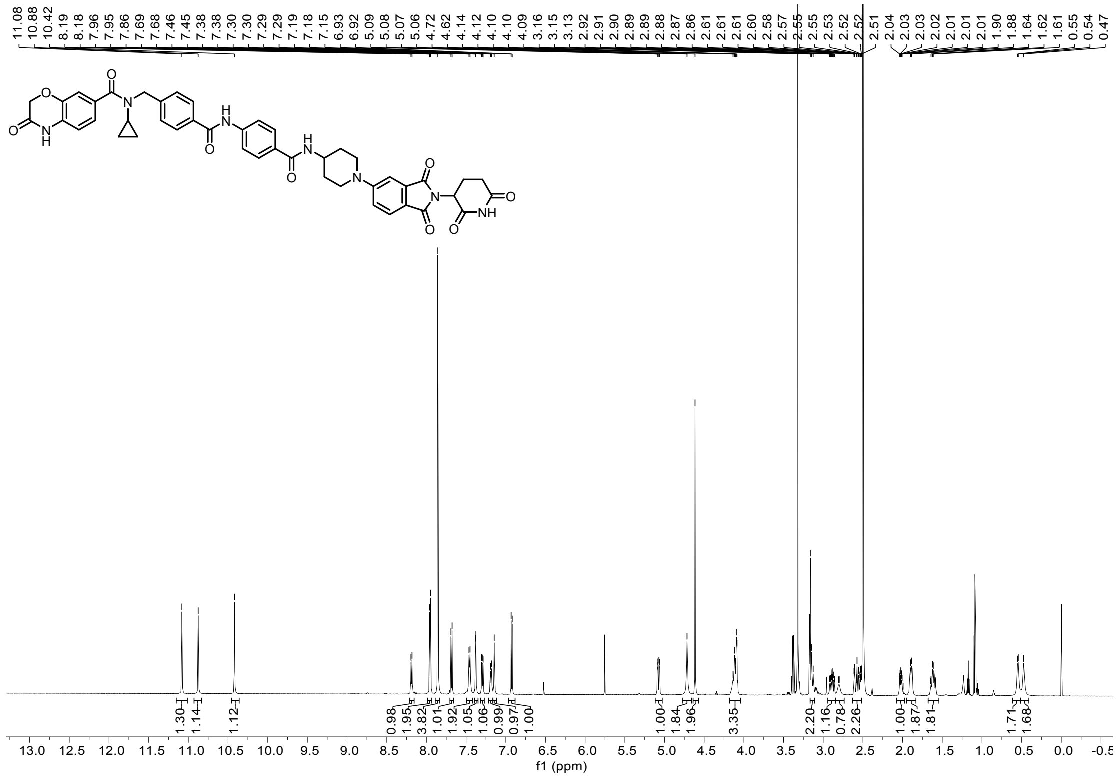

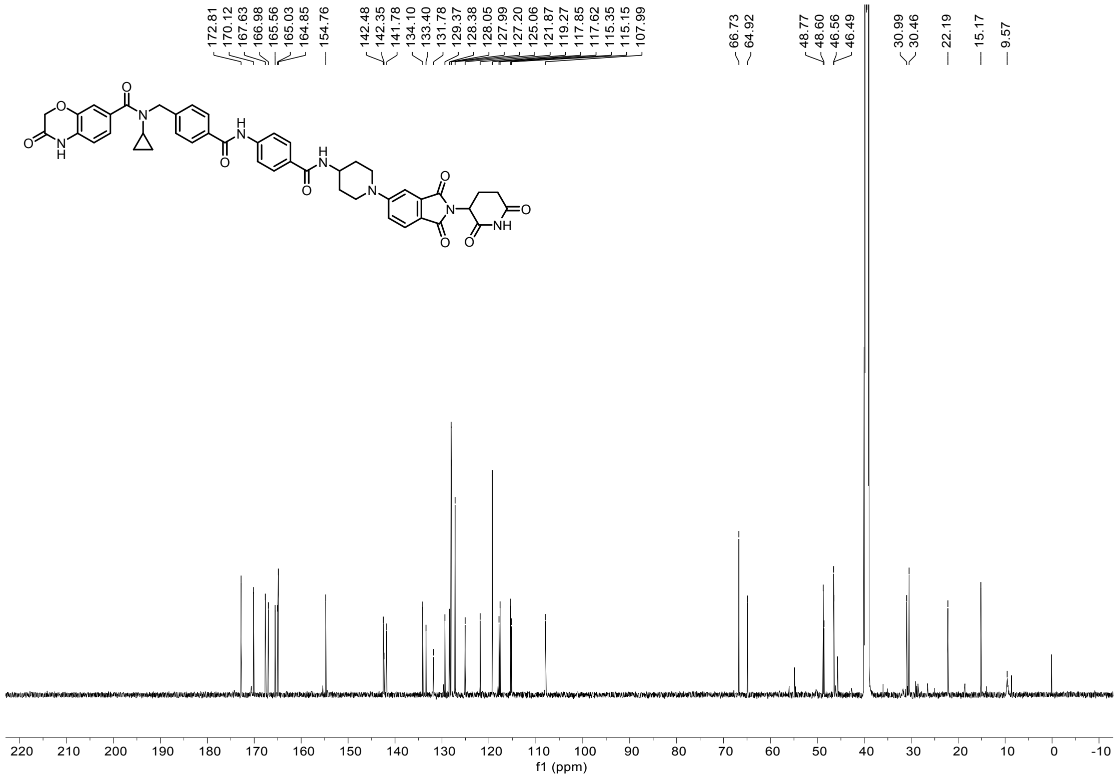

^{1}H NMR and ^{13}C NMR spectra for **10m**

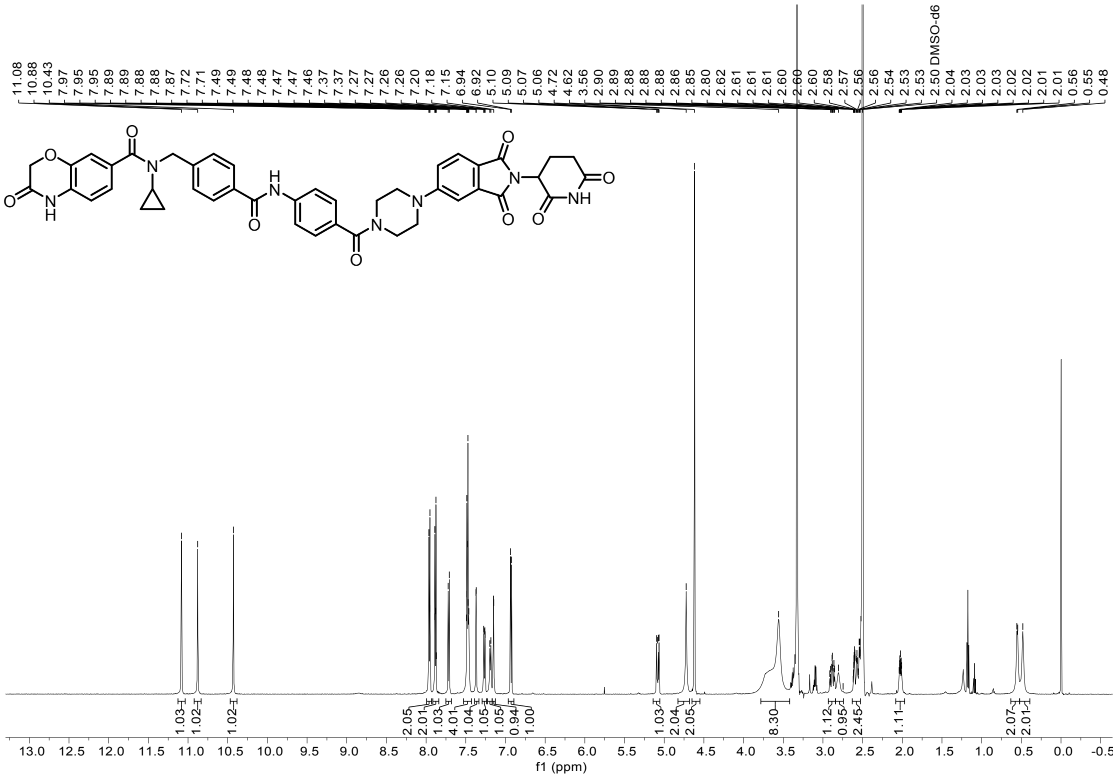

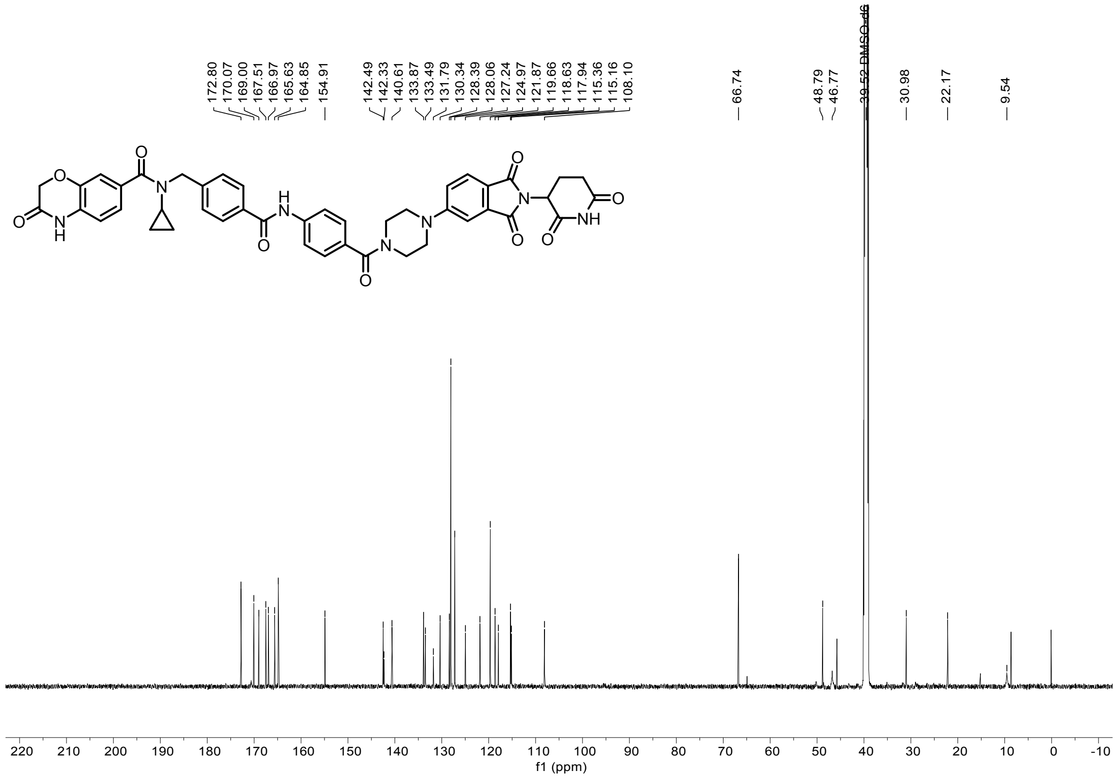

^{1}H NMR and ^{13}C NMR spectra for **10n**

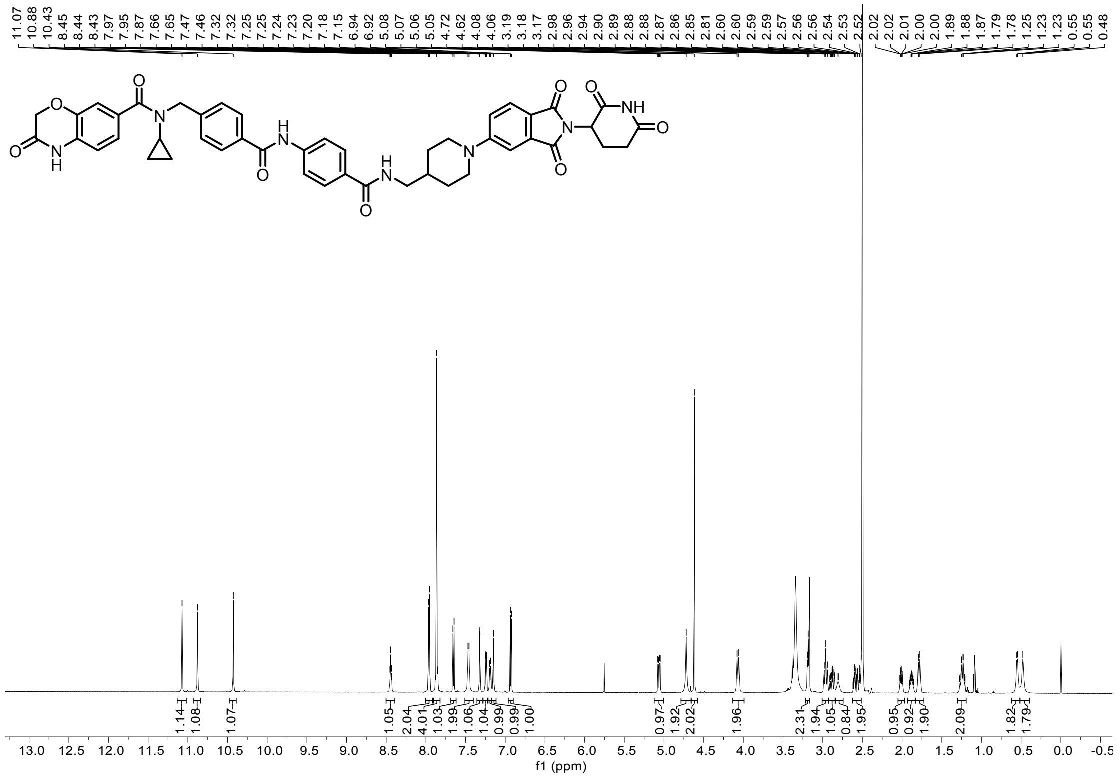

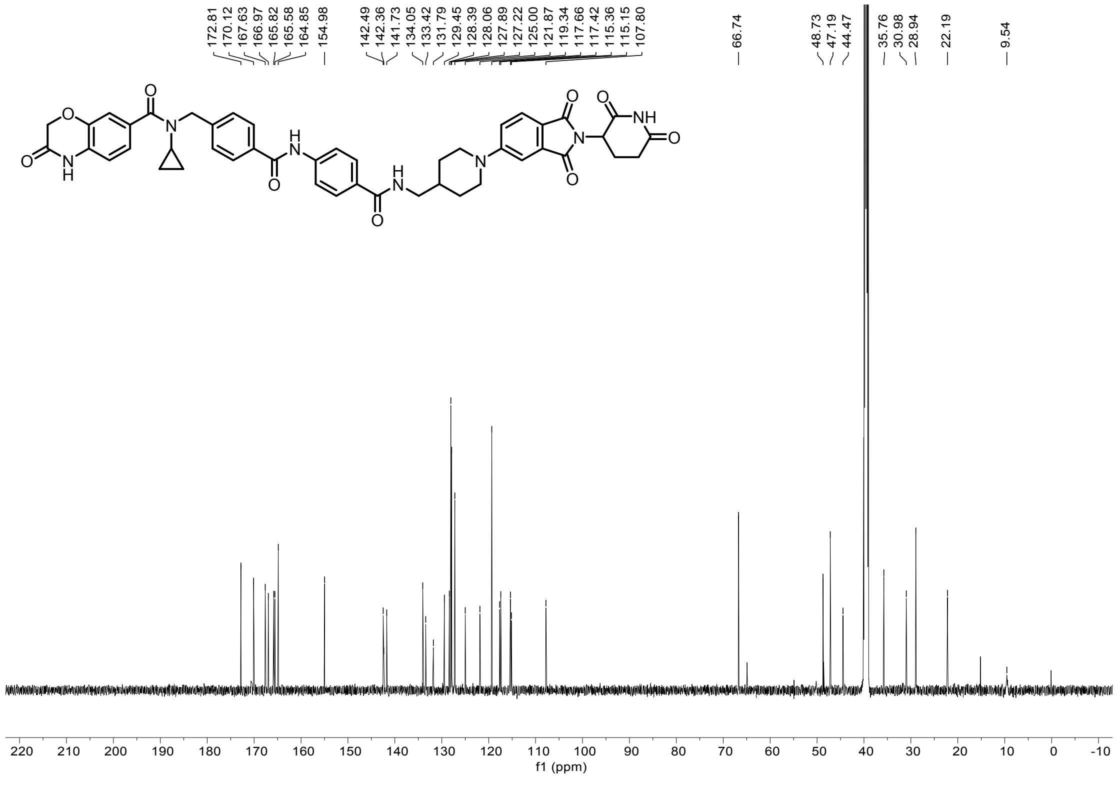

^{1}H NMR and ^{13}C NMR spectra for **10o**

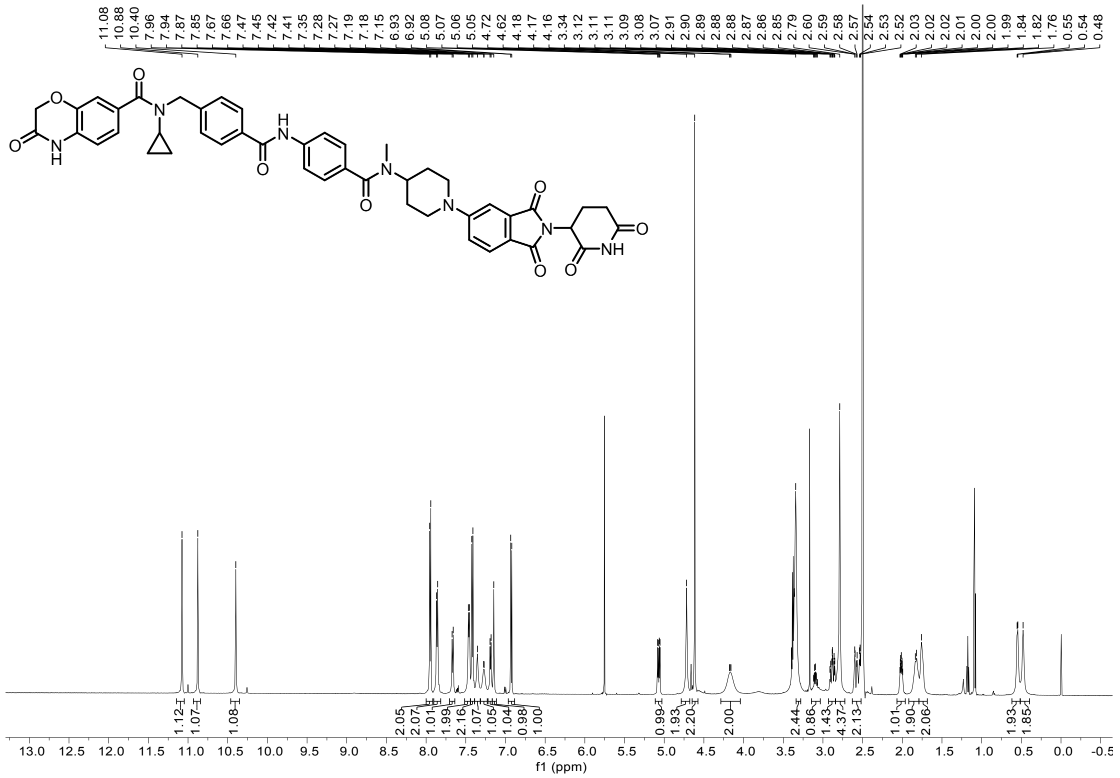

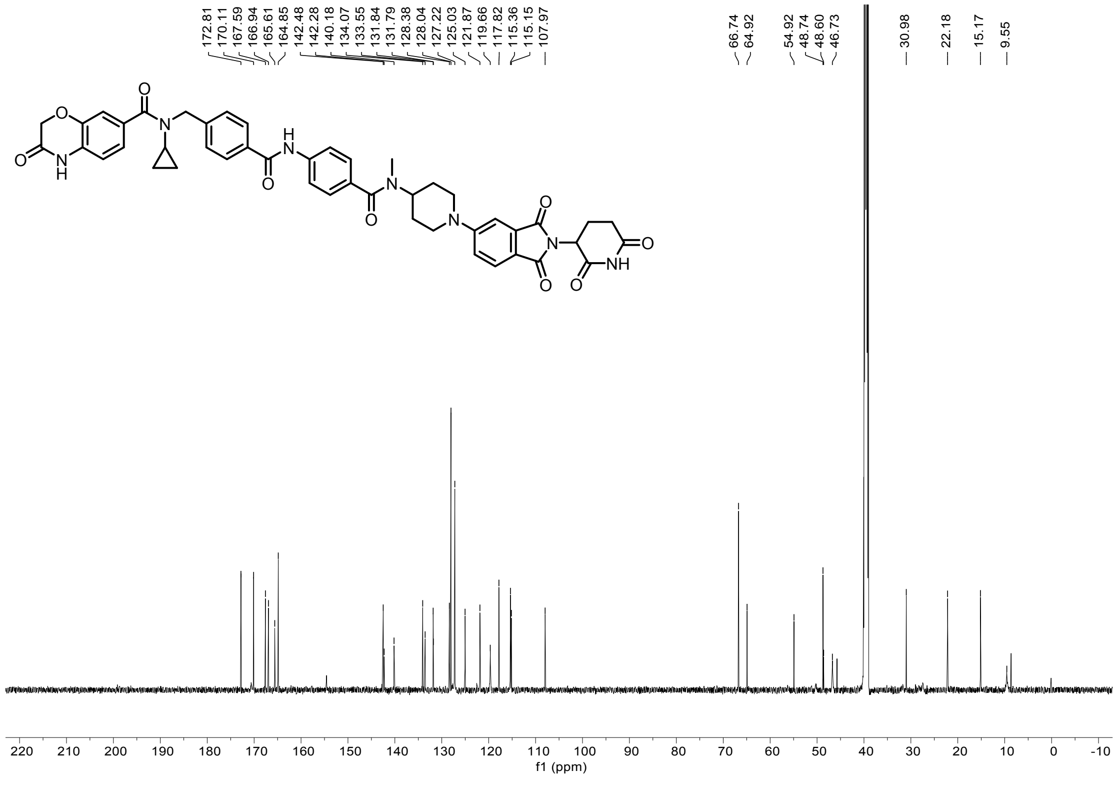

^{1}H NMR and ^{13}C NMR spectra for **10p**

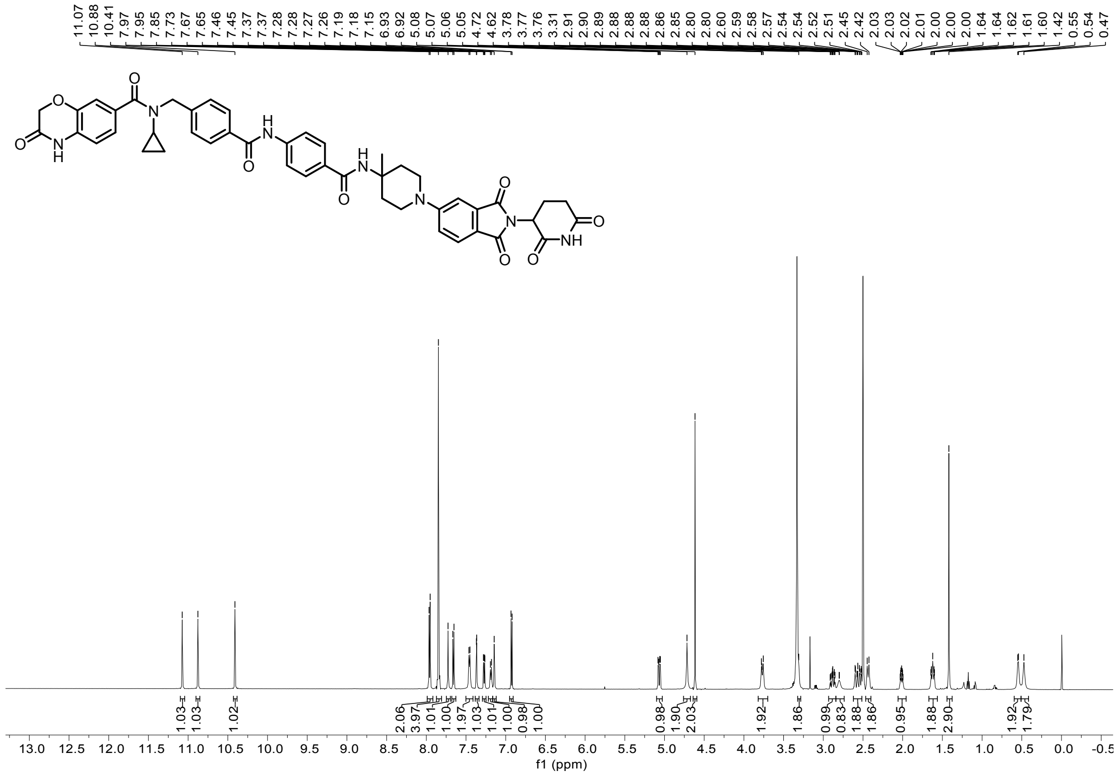

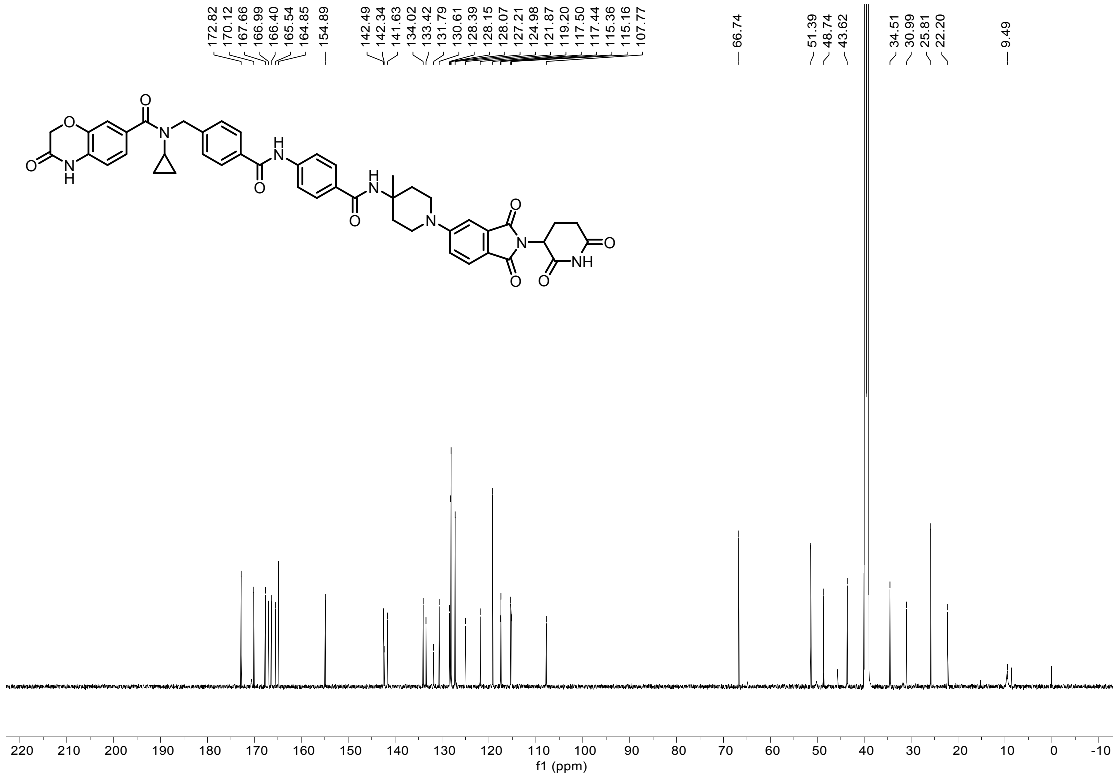

^{1}H NMR and ^{13}C NMR spectra for **10q**

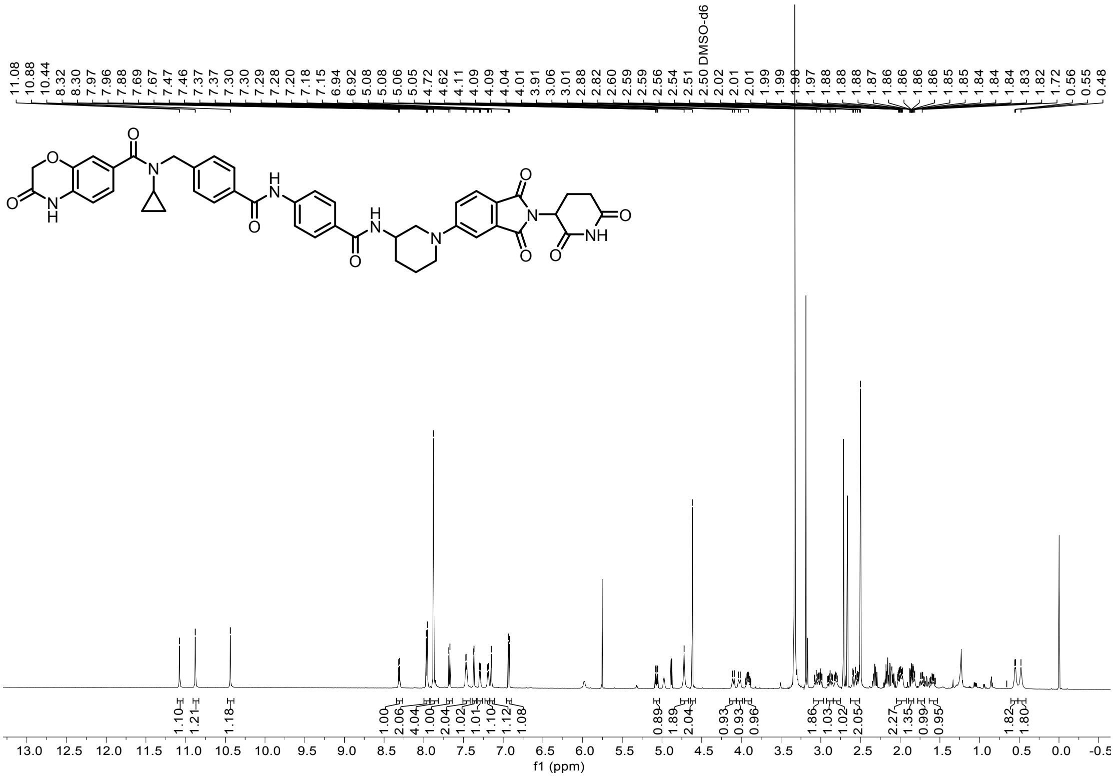

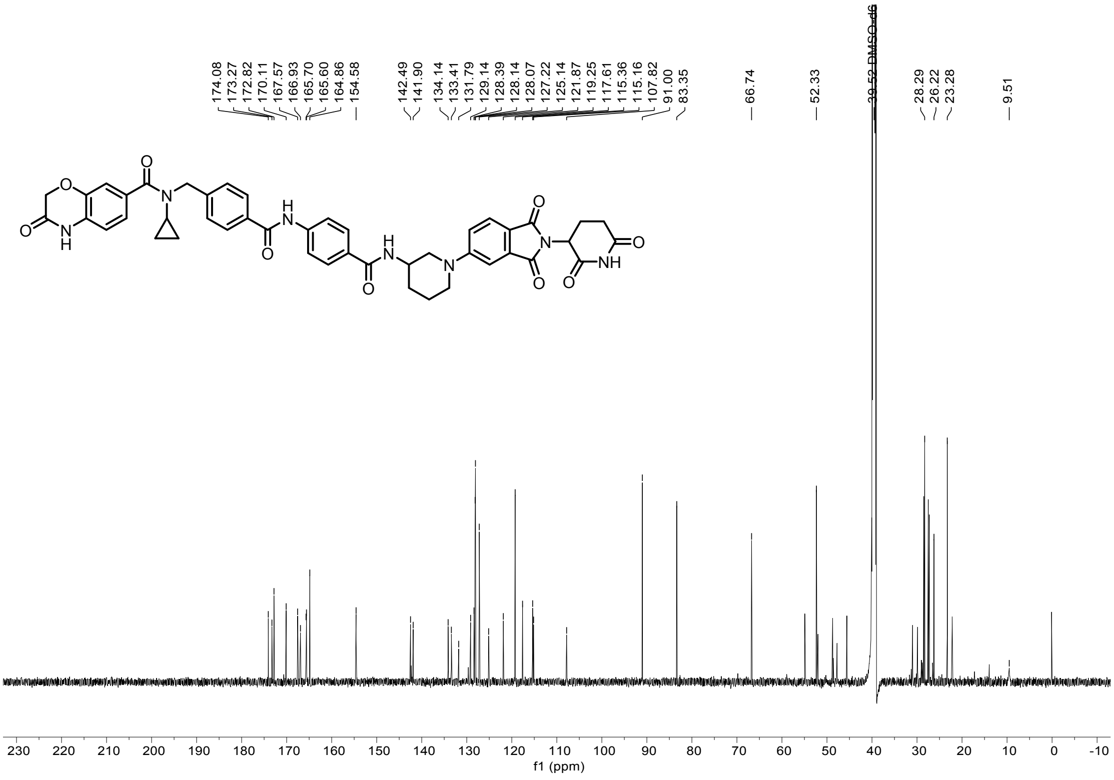

HPLC spectra of final compounds

HPLC Purity Analysis of **10a**

**Data file:**	llc-1-39-hplc.dx		
**Sequence Name:**	llc-1-39-hplc	**Project Name:**	1260
**Sample name:**	llc-1-39-hplc	**Operator:**	SYSTEM
**Instrument:**	1260	**Injection date:**	2022-03-11 09:14:27+08:00
**Inj. volume:**	10.000 µL	**Location:**	P1-A1
**Acq. method:**	normal.amx	**Type:**	Sample
**Processing method:**	GC_LC 面积百分比_DefaultMethod.pmx	**Sample amount:**	0.00
**Manually modified:**	Manual Integration		

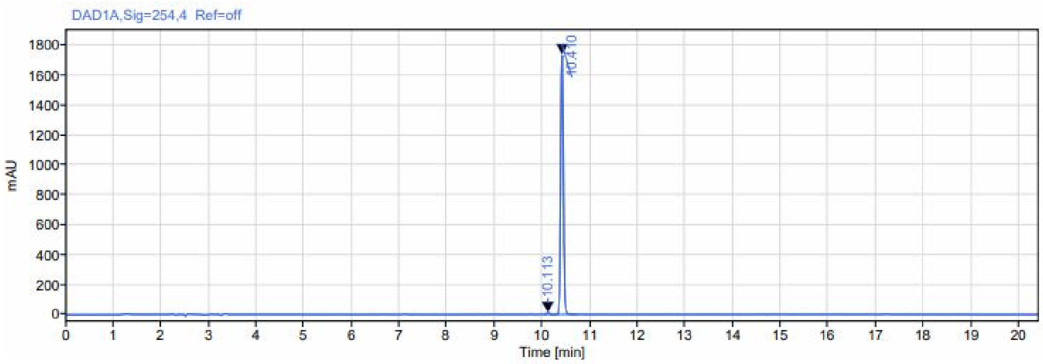

Signal: DAD1A,Sig=254,4 Ref=off

RT [min]	Type	Width [min]	Area	Height	Area%	Name
10.113	VV	0.19	48.74	12.04	0.71	
10.410	VV	0.49	6835.81	1736.69	99.29	
		**Sum**	6884.55		100.00	

HPLC Purity Analysis of **10b**

**Data file:**	llc-1-44-hplc.dx		
**Sequence Name:**	llc-1-44-45-46-47-hplc	**Project Name:**	1260
**Sample name:**	llc-1-44-hplc	**Operator:**	SYSTEM
**Instrument:**	1260	**Injection date:**	2022-03-10 11:16:41+08:00
**Inj. volume:**	1.000 µL	**Location:**	P1-A2
**Acq. method:**	normal.amx	**Type:**	Sample
**Processing method:**	GC_LC 面积百分比_DefaultMethod.pmx	**Sample amount:**	0.00
**Manually modified:**	Manual Integration		

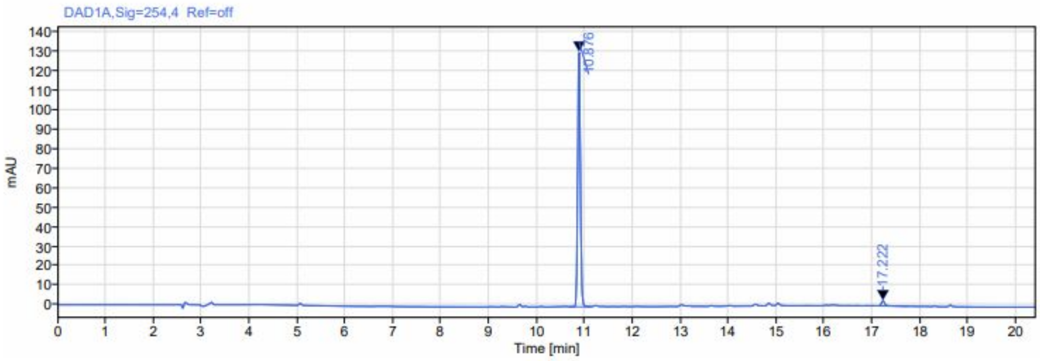

Signal: DAD1A,Sig=254,4 Ref=off

RT [min]	Type	Width [min]	Area	Height	Area%	Name
10.876	VV	0.45	518.22	130.80	97.36	
17.222	BB	0.64	14.03	2.65	2.64	
		**Sum**	532.25		100.00	

HPLC Purity Analysis of **10c**

**Data file:**	llc-1-45-hplc.dx		
**Sequence Name:**	llc-1-44-45-46-47-hplc	**Project Name:**	1260
**Sample name:**	llc-1-45-hplc	**Operator:**	SYSTEM
**Instrument:**	1260	**Injection date:**	2022-03-10 11:41:37+08:00
**Inj. volume:**	1.000 µL	**Location:**	P1-A3
**Acq. method:**	normal.amx	**Type:**	Sample
**Processing method:**	GC_LC 面积百分比_DefaultMethod.pmx	**Sample amount:**	0.00
**Manually modified:**	Manual Integration		

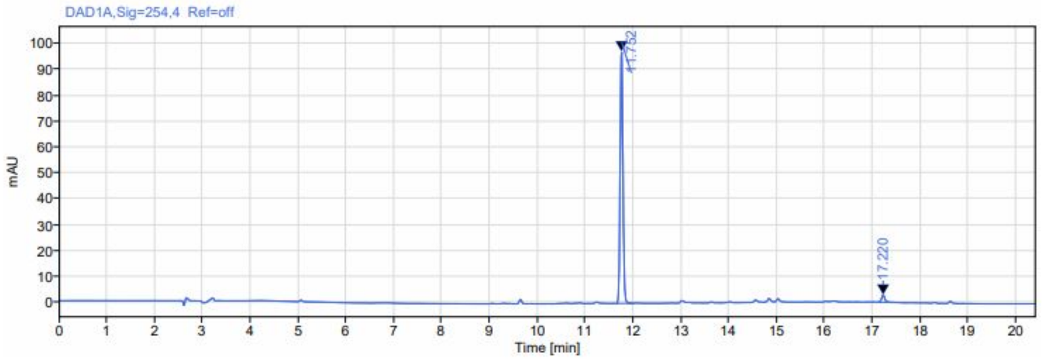

Signal: DAD1A,Sig=254,4 Ref=off

RT [min]	Type	Width [min]	Area	Height	Area%	Name
11.752	BV	0.37	398.03	97.31	96.47	
17.220	BB	0.63	14.54	2.79	3.53	
		**Sum**	412.57		100.00	

HPLC Purity Analysis of **10d**

**Data file:**	llc-1-46-hplc.dx		
**Sequence Name:**	llc-1-44-45-46-47-hplc	**Project Name:**	1260
**Sample name:**	llc-1-46-hplc	**Operator:**	SYSTEM
**Instrument:**	1260	**Injection date:**	2022-03-10 12:06:32+08:00
**Inj. volume:**	1.000 µL	**Location:**	P1-A4
**Acq. method:**	normal.amx	**Type:**	Sample
**Processing method:**	GC_LC 面积百分比_DefaultMethod.pmx	**Sample amount:**	0.00
**Manually modified:**	Manual Integration		

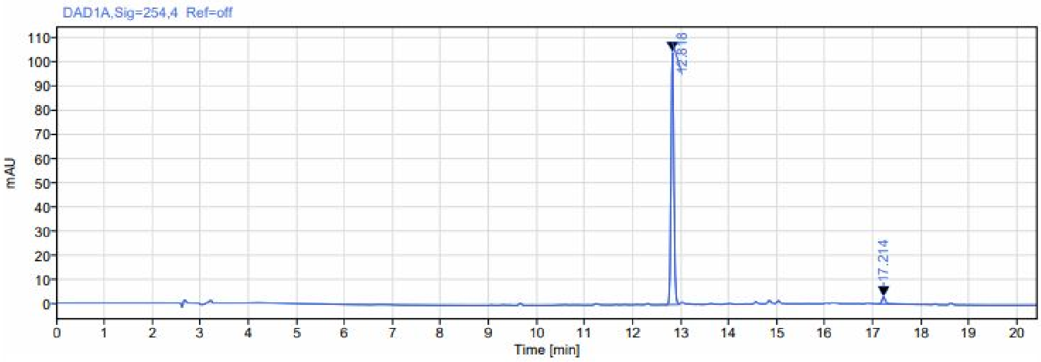

Signal: DAD1A,Sig=254,4 Ref=off

RT [min]	Type	Width [min]	Area	Height	Area%	Name
12.818	BV	0.29	446.41	104.48	96.50	
17.214	VB	0.64	16.18	2.93	3.50	
		**Sum**	462.59		100.00	

HPLC Purity Analysis of **10e**

**Data file:**	llc-1-49-1.dx		
**Sequence Name:**	llc-1-49-52-53	**Project Name:**	1260
**Sample name:**	llc-1-49-1	**Operator:**	SYSTEM
**Instrument:**	1260	**Injection date:**	2022-03-18 14:53:04+08:00
**Inj. volume:**	10.000 µL	**Location:**	P1-A2
**Acq. method:**	normal.amx	**Type:**	Sample
**Processing method:**	GC_LC 面积百分比_DefaultMethod.pmx	**Sample amount:**	0.00
**Manually modified:**	Manual Integration		

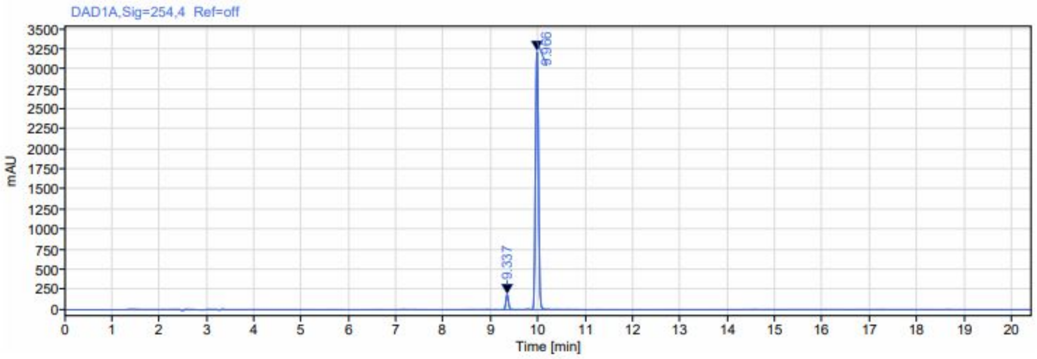

Signal: DAD1A,Sig=254,4 Ref=off

RT [min]	Type	Width [min]	Area	Height	Area%	Name
9.337	BB	0.38	712.12	187.50	4.75	
9.966	BV	0.30	14274.31	3222.99	95.25	
		**Sum**	14986.43		100.00	

HPLC Purity Analysis of **10f**

**Data file:**	llc-1-81.dx		
**Sequence Name:**	LLC-1-81-83	**Project Name:**	1260
**Sample name:**	llc-1-81	**Operator:**	SYSTEM
**Instrument:**	1260	**Injection date:**	2022-06-13 21:37:06+08:00
**Inj. volume:**	10.000 µL	**Location:**	P1-A1
**Acq. method:**	normal.amx	**Type:**	Sample
**Processing method:**	GC_LC Area Percent_DefaultMethod.pmx	**Sample amount:**	0.00
**Manually modified:**	Manual Integration		

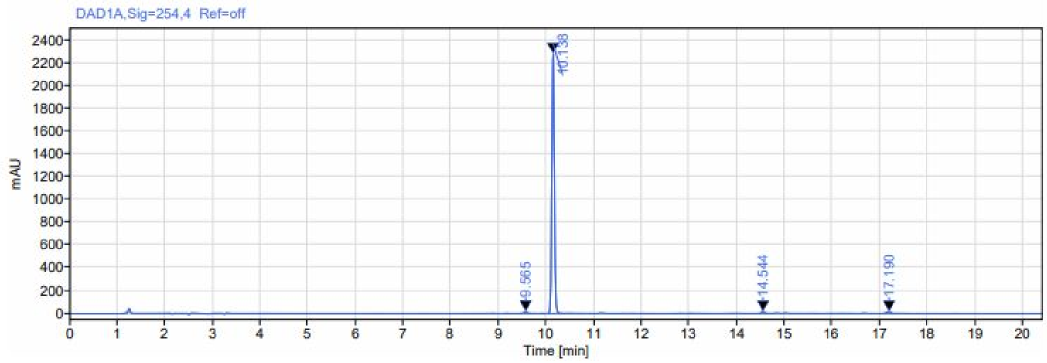

Signal: DAD1A,Sig=254,4 Ref=off

RT [min]	Type	Width [min]	Area	Height	Area%	Name
9.565	BV	0.32	82.93	19.10	0.92	
10.138	BV	0.35	8763.43	2284.61	97.03	
14.544	BB	0.25	79.61	17.70	0.88	
17.190	VB	0.55	105.51	17.81	1.17	
		**Sum**	9031.49		100.00	

HPLC Purity Analysis of **10g**

**Data file:**	llc-1-50.dx		
**Sequence Name:**	llc-1-49-50-51	**Project Name:**	1260
**Sample name:**	llc-1-50	**Operator:**	SYSTEM
**Instrument:**	1260	**Injection date:**	2022-03-17 11:21:56+08:00
**Inj. volume:**	10.000 µL	**Location:**	P1-A3
**Acq. method:**	normal.amx	**Type:**	Sample
**Processing method:**	GC_LC Area Percent_DefaultMethod.pmx	**Sample amount:**	0.00
**Manually modified:**	Manual Integration		

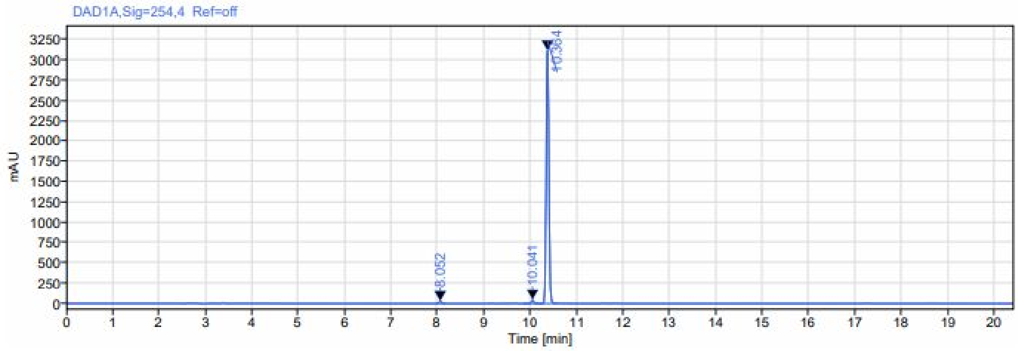

Signal: DAD1A,Sig=254,4 Ref=off

RT [min]	Type	Width [min]	Area	Height	Area%	Name
8.052	VM m	0.21	100.08	26.53	0.79	
10.041	BB	0.39	165.01	40.46	1.29	
10.364	BV	0.32	12482.30	3117.47	97.92	
		**Sum**	12747.39		100.00	

HPLC Purity Analysis of **10h**

**Data file:**	llc-1-83.dx		
**Sequence Name:**	LLC-1-81-83	**Project Name:**	1260
**Sample name:**	llc-1-83	**Operator:**	SYSTEM
**Instrument:**	1260	**Injection date:**	2022-06-13 22:02:08+08:00
**Inj. volume:**	10.000 µL	**Location:**	P1-A2
**Acq. method:**	normal.amx	**Type:**	Sample
**Processing method:**	GC_LC Area Percent_DefaultMethod.pmx	**Sample amount:**	0.00
**Manually modified:**	Manual Integration		

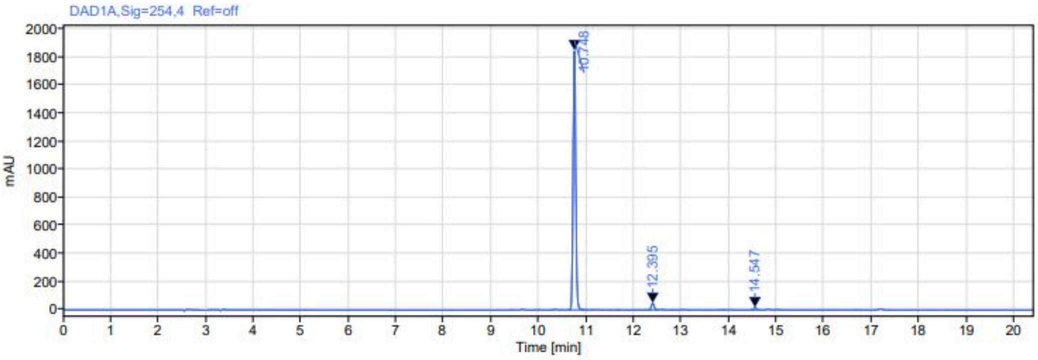

Signal: DAD1A,Sig=254,4 Ref=off

RT [min]	Type	Width [min]	Area	Height	Area%	Name
10.748	VB	0.40	7227.07	1845.43	96.29	
12.395	BV	0.29	203.46	47.61	2.71	
14.547	BV	0.23	75.24	17.01	1.00	
		**Sum**	7505.77		100.00	

HPLC Purity Analysis of **10i**

**Data file:**	llc-1-51.dx		
**Sequence Name:**	llc-1-49-50-51	**Project Name:**	1260
**Sample name:**	llc-1-51	**Operator:**	SYSTEM
**Instrument:**	1260	**Injection date:**	2022-03-17 11:46:58+08:00
**Inj. volume:**	10.000 µL	**Location:**	P1-A4
**Acq. method:**	normal.amx	**Type:**	Sample
**Processing method:**	GC_LC Area Percent_DefaultMethod.pmx	**Sample amount:**	0.00
**Manually modified:**	Manual Integration		

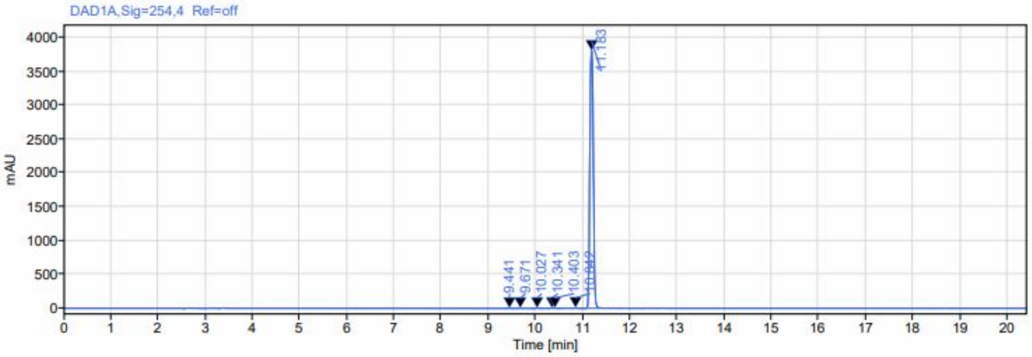

Signal: DAD1A,Sig=254,4 Ref=off

RT [min]	Type	Width [min]	Area	Height	Area%	Name
9.441	BB	0.17	12.10	3.30	0.06	
9.671	BV	0.15	14.29	3.86	0.07	
10.027	BV	0.26	23.70	4.19	0.12	
10.341	BV	0.13	18.77	4.78	0.09	
10.403	VB	0.15	17.00	4.47	0.09	
10.842	BB	0.28	27.33	6.94	0.14	
11.183	BV	0.41	19669.67	3805.62	99.43	
		**Sum**	19782.86		100.00	

HPLC Purity Analysis of **10j**

**Data file:**	llc-1-52.dx		
**Sequence Name:**	llc-1-52	**Project Name:**	1260
**Sample name:**	llc-1-52	**Operator:**	SYSTEM
**Instrument:**	1260	**Injection date:**	2022-03-21 19:42:25+08:00
**Inj. volume:**	10.000 µL	**Location:**	P1-A1
**Acq. method:**	normal.amx	**Type:**	Sample
**Processing method:**	GC_LC 面积百分比_DefaultMethod.pmx	**Sample amount:**	0.00
**Manually modified:**	Manual Integration		

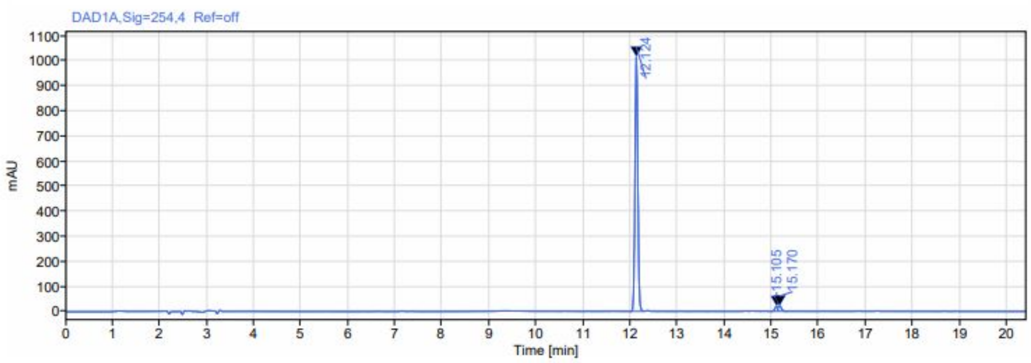

Signal: DAD1A,Sig=254,4 Ref=off

RT [min]	Type	Width [min]	Area	Height	Area%	Name
12.124	BV	0.33	4229.69	1014.15	95.94	
15.105	BV	0.16	78.56	19.17	1.78	
15.170	VV	0.19	100.46	21.04	2.28	
		**Sum**	4408.70		100.00	

HPLC Purity Analysis of **10k**

**Data file:**	LLC-4-22-2.dx		
**Sequence Name:**	SingleSample	**Project Name:**	1260
**Sample name:**	LLC-4-22-2	**Operator:**	SYSTEM
**Instrument:**	1260	**Injection date:**	2023-08-29 16:44:52+08:00
**Inj. volume:**	15.000 µL	**Location:**	P1-A2
**Acq. method:**	normal.amx	**Type:**	Sample
**Processing method:**	INM.pmx	**Sample amount:**	0.00
**Manually modified:**	Manual Integration		

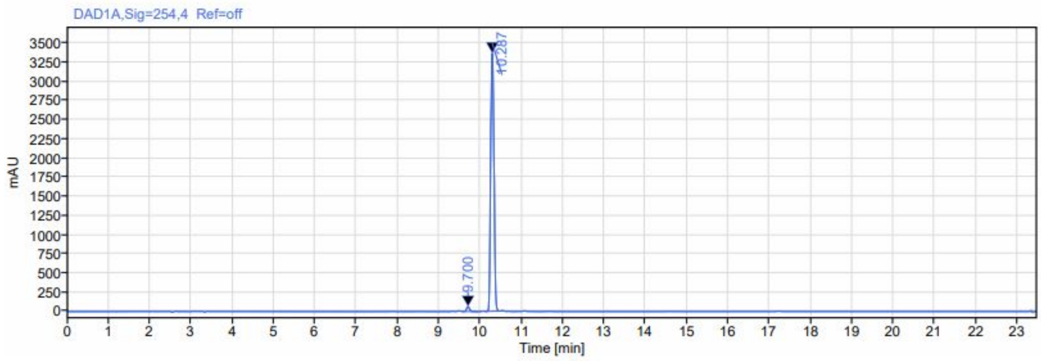

Signal: DAD1A,Sig=254,4 Ref=off

RT [min]	Type	Width [min]	Area	Height	Area%	Name
9.700	VB	0.38	341.48	65.88	1.77	
10.287	MM m	0.35	18988.12	3369.99	98.23	
		**Sum**	19329.59		100.00	

HPLC Purity Analysis of **10l**

**Data file:**	LLC-4-24.dx		
**Sequence Name:**	LLC-4-21-22-24	**Project Name:**	1260
**Sample name:**	LLC-4-24	**Operator:**	SYSTEM
**Instrument:**	1260	**Injection date:**	2022-11-06 17:22:46+08:00
**Inj. volume:**	10.000 µL	**Location:**	P1-D3
**Acq. method:**	normal.amx	**Type:**	Sample
**Processing method:**	GC_LC Area Percent_DefaultMethod.pmx	**Sample amount:**	0.00
**Manually modified:**	Manual Integration		

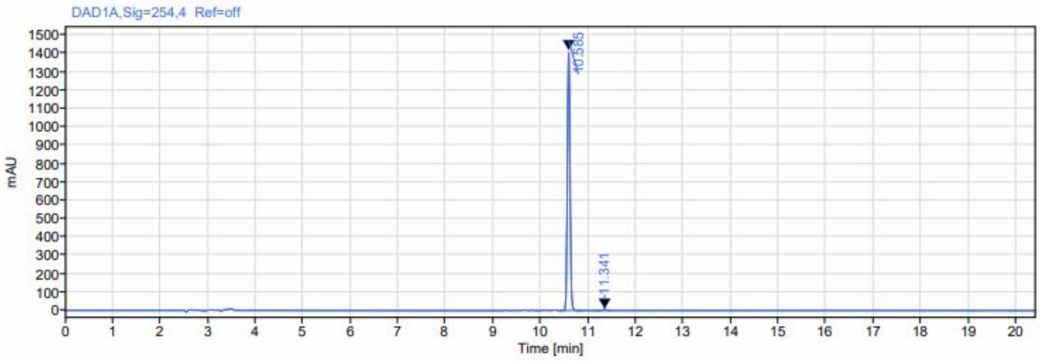

Signal: DAD1A,Sig=254,4 Ref=off

RT [min]	Type	Width [min]	Area	Height	Area%	Name
10.585	BV	0.44	5558.09	1416.37	99.41	
11.341	BV	0.31	33.16	7.45	0.59	
		**Sum**	5591.25		100.00	

HPLC Purity Analysis of **10m**

**Data file:**	LLC-4-26.dx		
**Sequence Name:**	SingleSample	**Project Name:**	1260
**Sample name:**	LLC-4-26	**Operator:**	SYSTEM
**Instrument:**	1260	**Injection date:**	2022-11-07 18:04:32+08:00
**Inj. volume:**	10.000 µL	**Location:**	P1-A1
**Acq. method:**	normal.amx	**Type:**	Sample
**Processing method:**	GC_LC 面积百分比_DefaultMethod.pmx	**Sample amount:**	0.00
**Manually modified:**	Manual Integration		

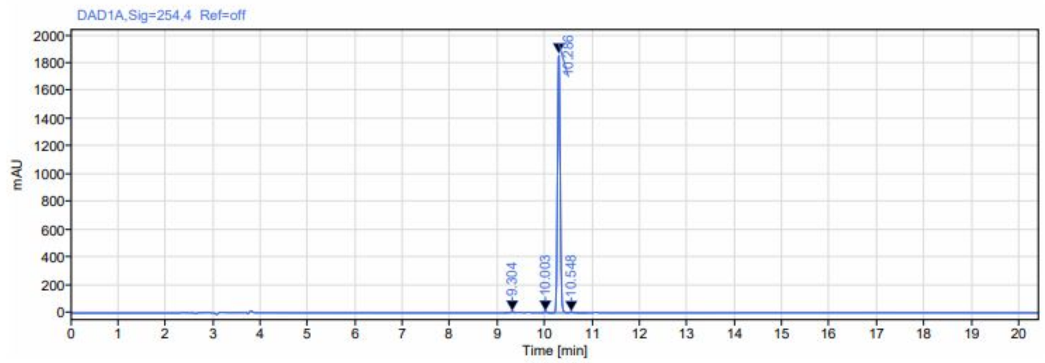

Signal: DAD1A,Sig=254,4 Ref=off

RT [min]	Type	Width [min]	Area	Height	Area%	Name
9.304	BB	0.24	48.71	10.96	0.66	
10.003	VB	0.24	51.59	11.95	0.70	
10.286	BV	0.31	7267.05	1866.05	97.99	
10.548	VB	0.41	48.68	8.49	0.66	
		**Sum**	7416.03		100.00	

HPLC Purity Analysis of **10n**

**Data file:**	LLC-5-51.dx		
**Sequence Name:**	SingleSample	**Project Name:**	1260
**Sample name:**	LLC-5-51	**Operator:**	SYSTEM
**Instrument:**	1260	**Injection date:**	2023-03-12 19:52:44+08:00
**Inj. volume:**	10.000 µL	**Location:**	P1-A1
**Acq. method:**	normal.amx	**Type:**	Sample
**Processing method:**	GC_LC 面积百分比_DefaultMethod.pmx	**Sample amount:**	0.00
**Manually modified:**	Manual Integration		

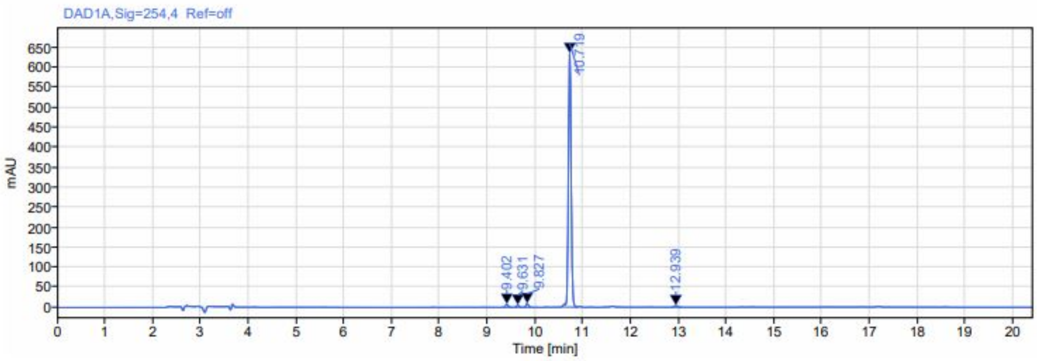

Signal: DAD1A,Sig=254,4 Ref=off

RT [min]	Type	Width [min]	Area	Height	Area%	Name
9.402	BV	0.24	29.73	7.57	1.12	
9.631	VV	0.19	22.21	5.60	0.84	
9.827	VV	0.18	37.24	9.43	1.41	
10.719	MV m	0.33	2537.28	634.52	95.95	
12.939	BB	0.23	17.92	4.11	0.68	
		**Sum**	2644.38		100.00	

HPLC Purity Analysis of **10o**

**Data file:**	LLC-5-63.dx		
**Sequence Name:**	SingleSample	**Project Name:**	1260
**Sample name:**	LLC-5-63	**Operator:**	SYSTEM
**Instrument:**	1260	**Injection date:**	2023-03-19 16:40:05+08:00
**Inj. volume:**	15.000 µL	**Location:**	P1-A1
**Acq. method:**	normal.amx	**Type:**	Sample
**Processing method:**	GC_LC 面积百分比_DefaultMethod.pmx	**Sample amount:**	0.00
**Manually modified:**	Manual Integration		

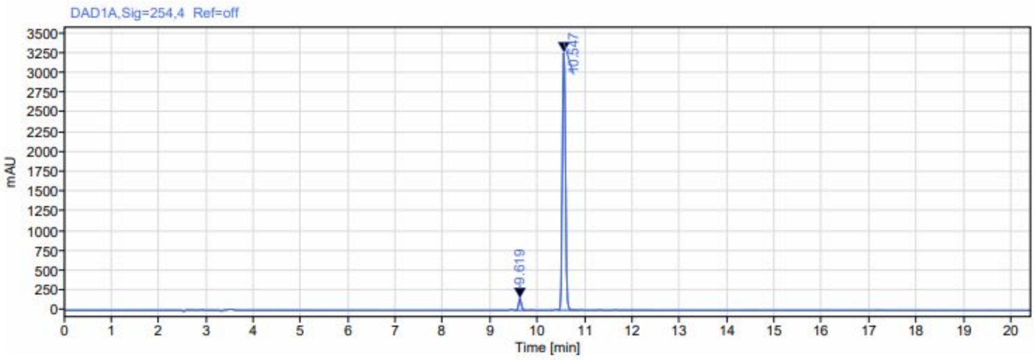

Signal: DAD1A,Sig=254,4 Ref=off

RT [min]	Type	Width [min]	Area	Height	Area%	Name
9.619	VV	0.23	609.86	150.31	3.94	
10.547	VB	0.41	14874.62	3253.69	96.06	
		**Sum**	15484.48		100.00	

HPLC Purity Analysis of **10p**

**Data file:**	LLC-6-35.dx		
**Sequence Name:**	SingleSample	**Project Name:**	1260
**Sample name:**	LLC-6-35	**Operator:**	SYSTEM
**Instrument:**	1260	**Injection date:**	2023-04-22 11:18:46+08:00
**Inj. volume:**	10.000 µL	**Location:**	P1-E1
**Acq. method:**	normal.amx	**Type:**	Sample
**Processing method:**	INM.pmx	**Sample amount:**	0.00
**Manually modified:**	Manual Integration		

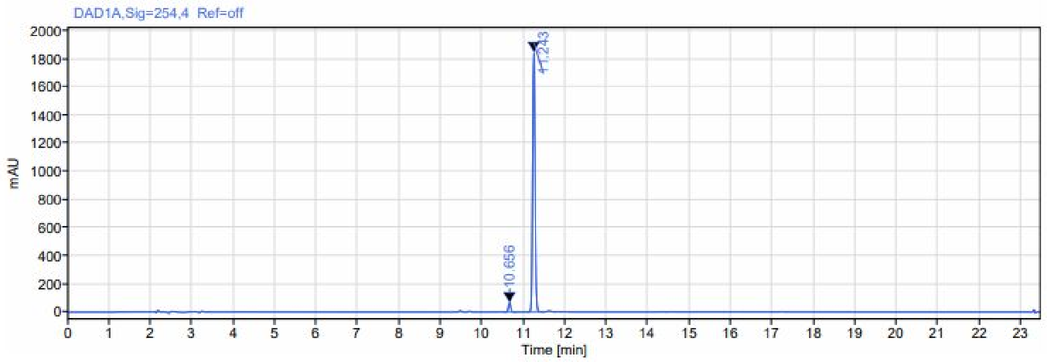

Signal: DAD1A,Sig=254,4 Ref=off

RT [min]	Type	Width [min]	Area	Height	Area%	Name
10.656	VV	0.29	257.75	64.16	3.31	
11.243	VV	0.41	7522.78	1845.17	96.69	
		**Sum**	7780.53		100.00	

HPLC Purity Analysis of **10q**

**Data file:**	LLC-7-8.dx		
**Sequence Name:**	SingleSample	**Project Name:**	1260
**Sample name:**	LLC-7-8	**Operator:**	SYSTEM
**Instrument:**	1260	**Injection date:**	2023-07-24 18:25:28+08:00
**Inj. volume:**	10.000 µL	**Location:**	P1-D1
**Acq. method:**	normal.amx	**Type:**	Sample
**Processing method:**	INM.pmx	**Sample amount:**	0.00
**Manually modified:**	Manual Integration		

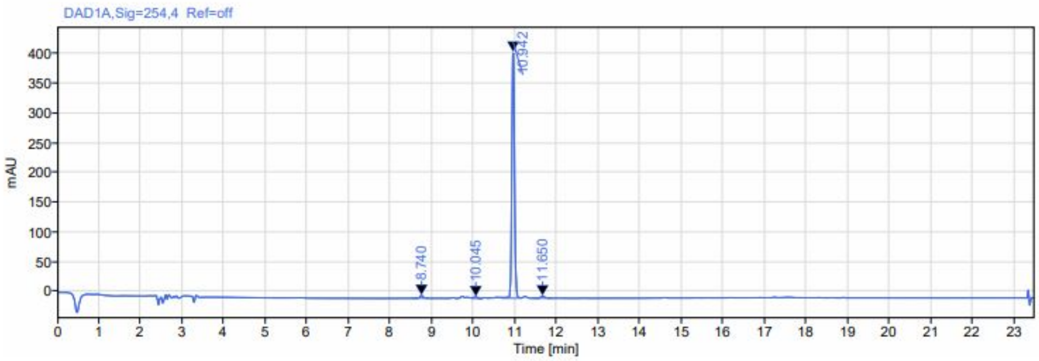

Signal: DAD1A,Sig=254,4 Ref=off

RT [min]	Type	Width [min]	Area	Height	Area%	Name
8.740	MM m	0.38	25.39	4.82	1.33	
10.045	VB	0.24	18.65	2.95	0.98	
10.942	VV	0.47	1843.76	414.53	96.45	
11.650	BB	0.43	23.74	3.81	1.24	
		**Sum**	1911.54		100.00	

^{1}H and ^{13}C NMR Spectra and HPLC spectra of LLC0424N (10lN) and LLC0877

^{1}H NMR and ^{13}C NMR spectra for **10lN**

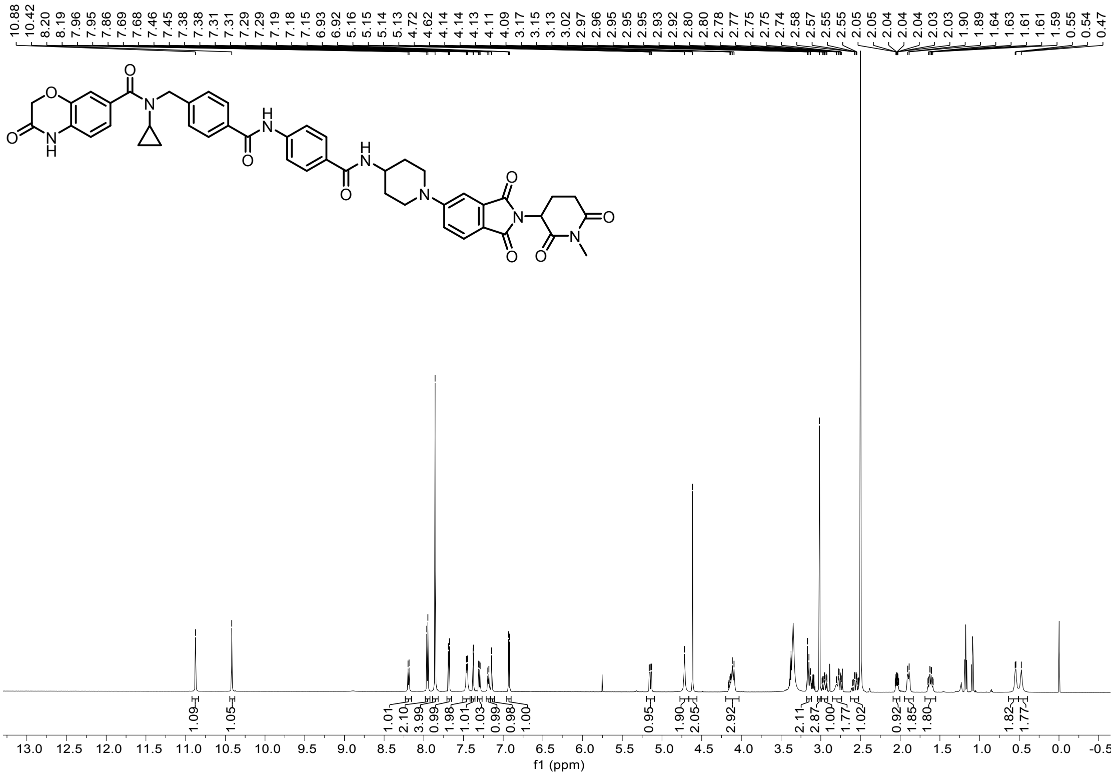

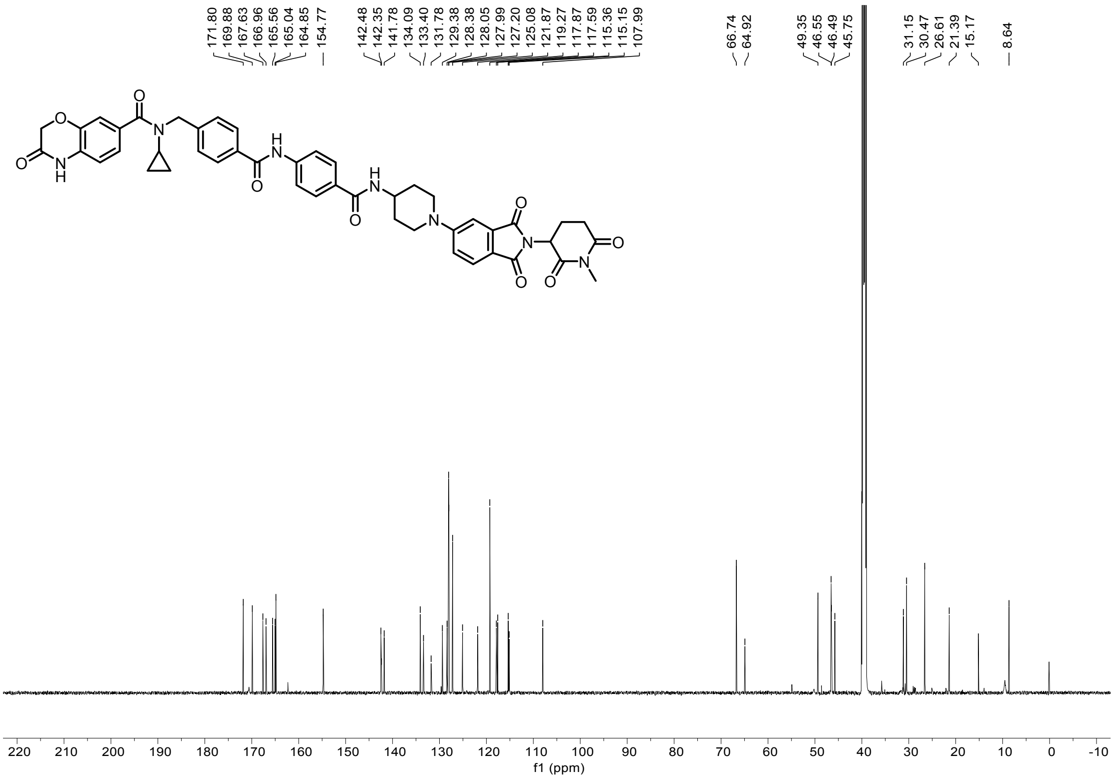

HPLC Purity Analysis of **10lN**

**Data file:**	LLC-5-43.dx		
**Sequence Name:**	SingleSample	**Project Name:**	1260
**Sample name:**	LLC-5-43	**Operator:**	SYSTEM
**Instrument:**	1260	**Injection date:**	2023-03-07 21:12:46+08:00
**Inj. volume:**	15.000 µL	**Location:**	P1-C1
**Acq. method:**	normal.amx	**Type:**	Sample
**Processing method:**	GC_LC 面积百分比_DefaultMethod.pmx	**Sample amount:**	0.00
**Manually modified:**	Manual Integration		

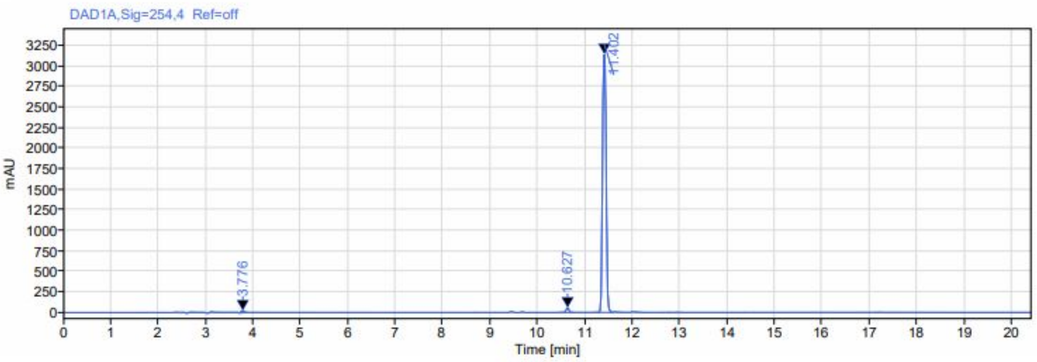

Signal: DAD1A,Sig=254,4 Ref=off

RT [min]	Type	Width [min]	Area	Height	Area%	Name
3.776	MM m	0.17	117.15	24.56	0.69	
10.627	VV	0.20	232.55	55.50	1.37	
11.402	BV	0.34	16571.30	3145.47	97.93	
		**Sum**	16921.00		100.00	

^{1}H NMR and ^{13}C NMR spectra for **LLC0877**

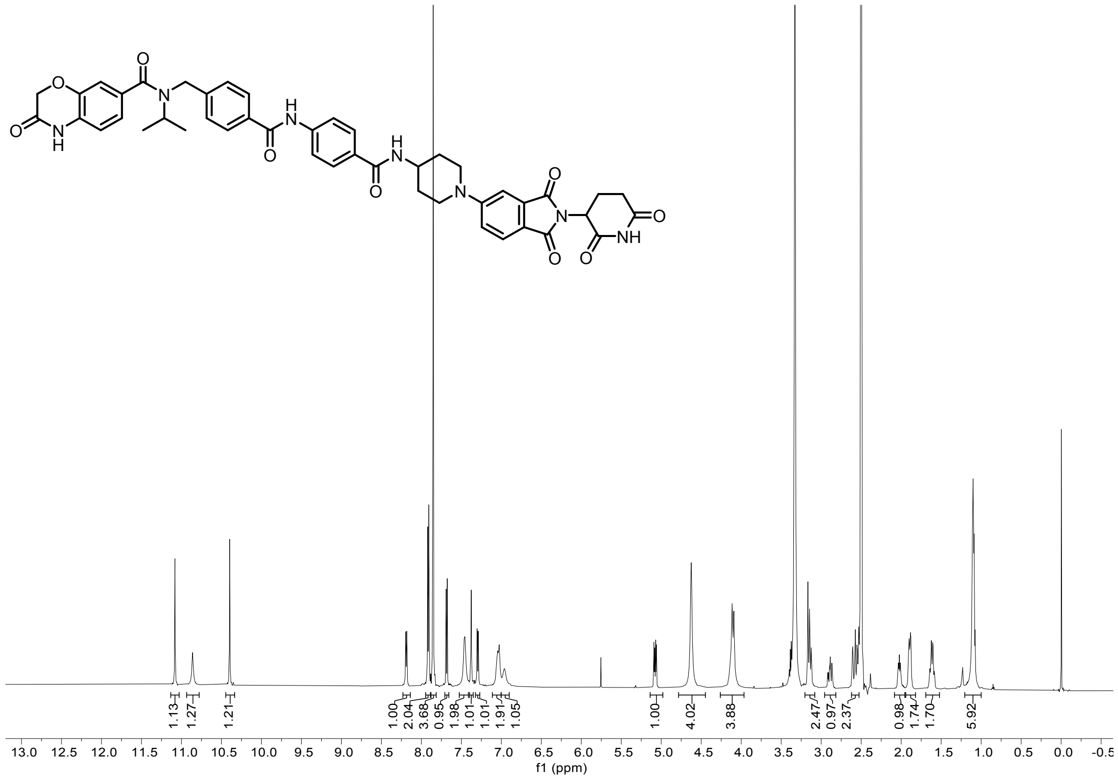

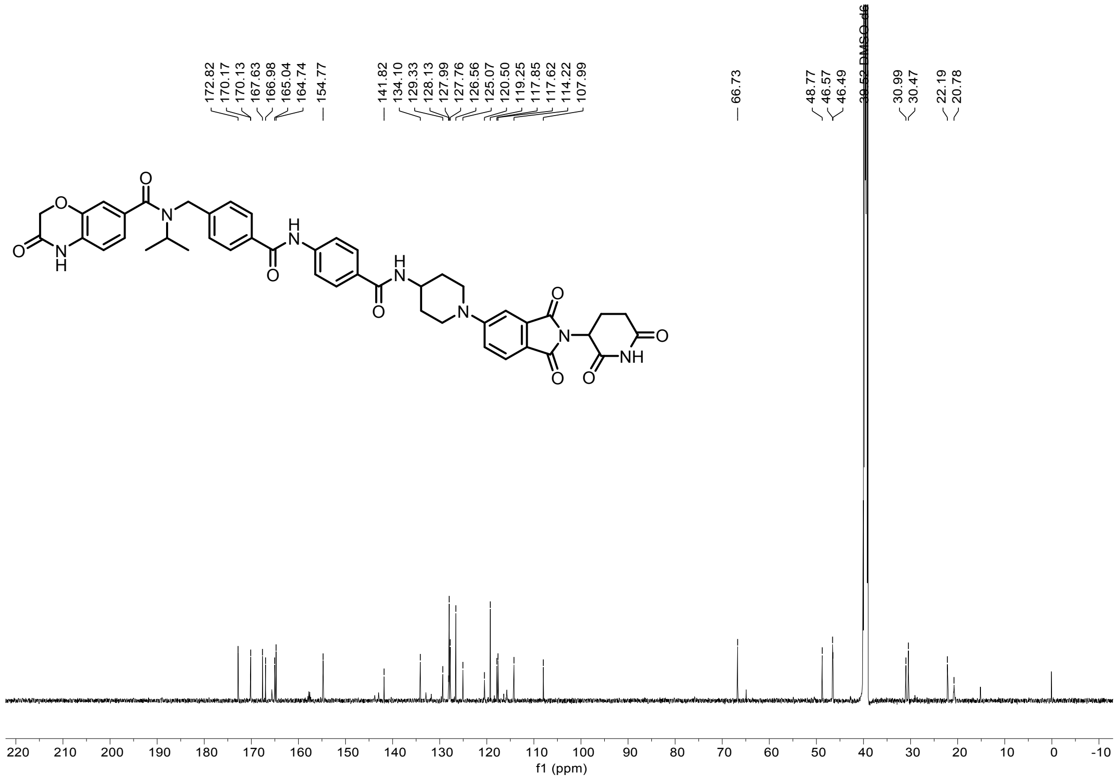

HPLC Purity Analysis of **LLC0877**

**Data file:**	LLC-8-77-2.dx		
**Sequence Name:**	SingleSample	**Project Name:**	1260
**Sample name:**	LLC-8-77-2	**Operator:**	SYSTEM
**Instrument:**	1260	**Injection date:**	2023-11-10 15:56:45+08:00
**Inj. volume:**	8.000 µL	**Location:**	P1-C1
**Acq. method:**	normal.amx	**Type:**	Sample
**Processing method:**	INM.pmx	**Sample amount:**	0.00
**Manually modified:**	Manual Integration		

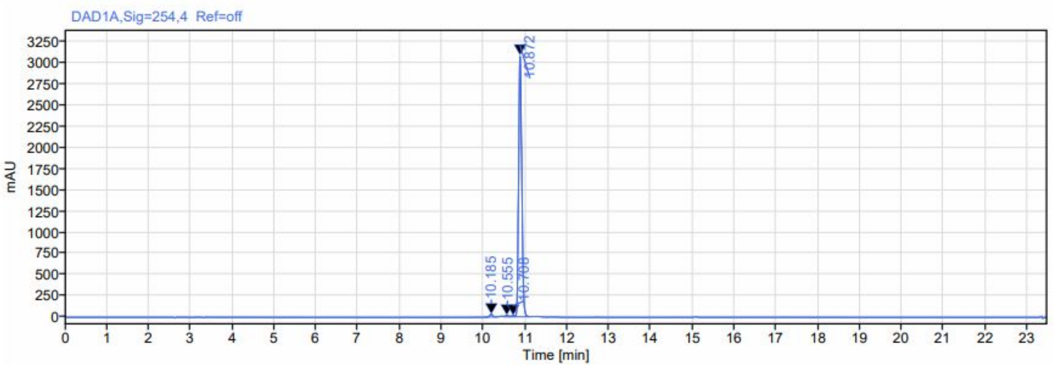

Signal: DAD1A,Sig=254,4 Ref=off

RT [min]	Type	Width [min]	Area	Height	Area%	Name
10.185	MM m	0.25	172.40	33.83	1.04	
10.555	VV	0.16	107.08	17.98	0.65	
10.708	VV	0.11	70.05	14.77	0.42	
10.872	VB	0.36	16211.68	3083.81	97.89	
		**Sum**	16561.21		100.00	
